# Stereoselective synthesis of hernandulcin, peroxylippidulcine A, lippidulcines A, B and C and taste evaluation

**DOI:** 10.3762/bjoc.11.228

**Published:** 2015-11-05

**Authors:** Marco Giulio Rigamonti, Francesco Gilberto Gatti

**Affiliations:** 1 Chemistry Department “G. Natta”, Politecnico di Milano, Piazza Leonardo da Vinci 1, 20133 Milano, Italy

**Keywords:** ADH ketone reduction, cooling agent, Corey–Bakshi–Shibata reduction, dehydrogenation, hernandulcin, Kornblum–DeLaMare rearrangement, lippidulcines, Saegusa–Larock

## Abstract

The first stereoselective synthesis of lippidulcines A, B and C has been accomplished starting from (+)-hernandulcin, which has been prepared on a multigram scale. The previously assigned absolute configurations have been confirmed. The key steps of this synthesis are based on a modified version of the Kornblum–DeLaMare rearrangement, and on a highly regioselective and stereoselective ketone reduction with the MeCBS reagent. The taste evaluations indicate that none of these sesquiterpenes are sweet, instead the lippidulcine A is a cooling agent with a mint after taste.

## Introduction

It is a matter of fact that a large consumption of sucrose is strongly associated to a considerable number of undesirable health effects, among which cardiovascular diseases and dental caries are the most relevant. Moreover, the increasing number, especially in the western countries, of people with obesity and type-II diabetes has pushed the food industry to develop new low calorie sweeteners, better known as sugar substituents. Among all artificial sweeteners so far developed: aspartame, saccharin, acesulfame K and sucralose are undoubtedly the most popular. However, questions regarding the safety of these sweeteners are still largely argued from the scientific community [1]. Thus, the discovery of new sugar substituents has become a target of food industry, to this regard new sweet-tasting natural products might offer a valid alternative to the artificial ones [2–4].

**Figure 1:**
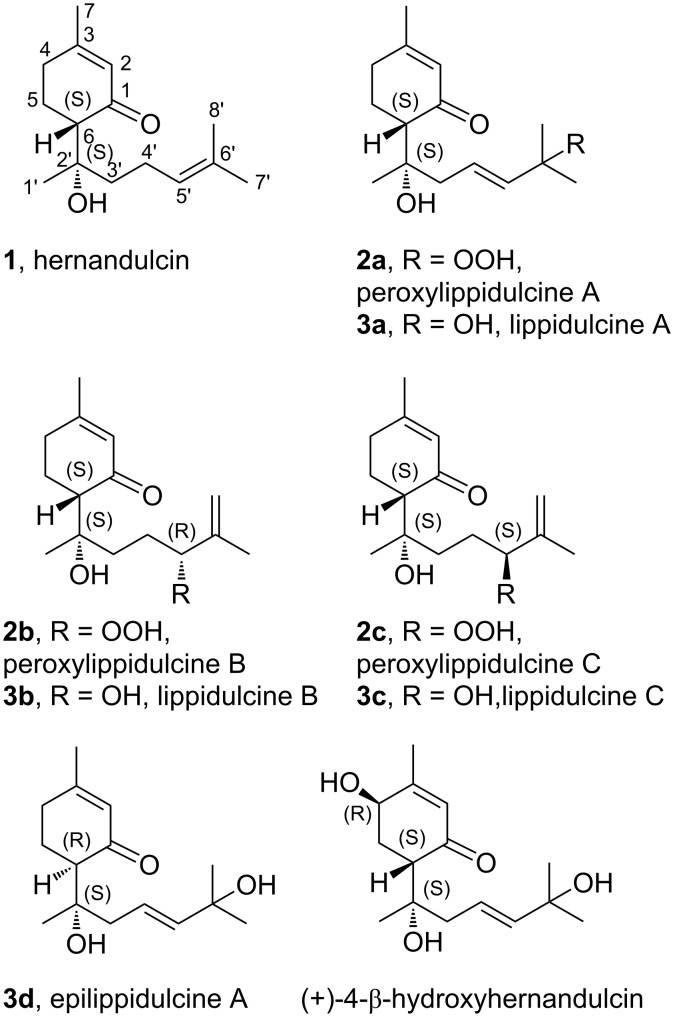
Hernandulcin and other bisabolanic derivatives extracted from *Lippia dulcis*.

At the beginning of 1980s Kinghorn et al. came across with an ancient botanical report describing the existence of a New World plant with comestible leaves having a very intense sweet taste. The Aztecs used to call this plant “*Tzonpelic xihuitl*”, which means sweet herb. However, for several centuries its unusual property has been incredibly forgotten until 1985; when the sesquiterpene **1** (Figure 1), isolated from the leaves and flowers of the Mexican plant *Lippia dulcis*, resulted the main vector of sweetness that Hernández described four hundred years before in his treatise [5]. Thus, in honor of the Spanish scientist, this natural product was called hernandulcin [6]. Hernandulcin is the first strongly sweet sesquiterpene, making it a breakthrough discovery in the field of natural sugar substitutes. It turned out that **1**, which belongs to the family of bisabolanes, is more than 1000 times as sweet as sucrose.

More recently other derivatives of **1** have been discovered, i.e., the peroxylippidulcines **2a–c** and the lippidulcines **3a–c** (Figure 1) [7–8]. However, these sesquiterpenes have been isolated in a so small amount that it has not been possible to assess their taste. In principle these bisabolanes could represent a very interesting structural variation of **1**, especially the lippidulcines, since the presence of a second hydroxy group should increase significantly their solubility in water, which is a key property for the sweeteners of beverages. In the following we report on the synthesis of hernadulcin and its hydroxy derivatives, i.e., the lippidulcines A, B and C and their taste evaluation.

## Results and Discussion

Since our synthetic strategy for the preparation of lippidulcines **3a**–**c** is based on the photooxygenation of **1**, it was mandatory to develop and optimize the synthesis of the latter on a multigram scale. Few years ago we have reported a new stereospecific synthesis of (+)-hernandulcin [9] starting from the very cheap commercially available (+)-isopulegol.

Mori [10–11] and Cheon [12–13] reported some syntheses of **1**. They epoxidized the side-chain olefinic double bonds of (+)-limonene or (−)-isopulegol with *m*-chloroperbenzoic acid (MCPBA) to introduce the stereogenic center at the C(2’) position. Unfortunately both methods were only modestly stereoselective and resulted in mixtures of the respective epoxides. These results implied a not simple column chromatographic separation of the diastereomeric mixtures. In contrast, we have shown that the Katsuki–Sharpless epoxidation of (+)-neoisopulegol (**4**) is much more selective [9].

**Scheme 1:**
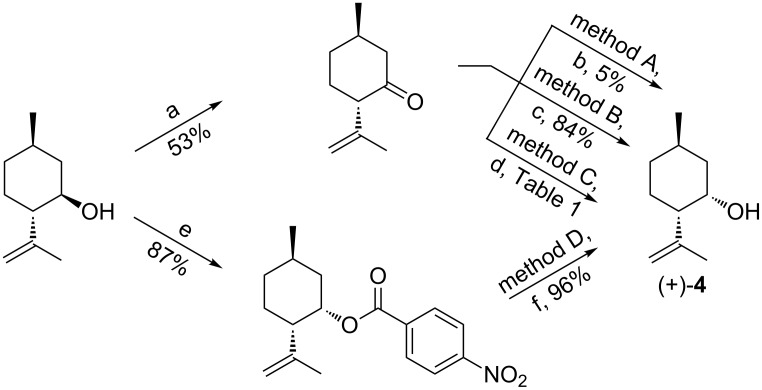
Synthesis of (+)-neoisopulegol. Reagents and conditions: (a) Jones reagent, acetone, 0 °C, 3 h; (b) DIBALH, 2-propanol, toluene, 0 °C/rt, 6 h; (c) *L*-selectride, THF, −78 °C/rt, 16 h; (d) ADH, cofactor NAD(P)H^+^, GDH, glucose, water, 30 °C, 24 h; (e) *p*-nitrobenzoic acid, PPh_3_, DIAD, toluene, 0 °C, 24 h; (f) MeONa, MeOH, rt, 5 h.

In the following we describe an improved version of our previously reported synthesis of **1**. First we have focused our initial efforts on the synthesis optimisation of the key intermediate **4** that can be prepared starting from (−)-isopulegol by two different approaches: i) oxidation of the hydroxy group to give (*S*)-isopulegone, which in turn is reduced stereospecifically into the desired *cis* diastereoisomer; or ii) by inversion of C(1) stereogenic center by means of a Mitsunobu reaction, followed by transesterification of the ester to give **4** (Scheme 1).

(*S*)-Isopulegone was prepared by Jones oxidation of (−)-isopulegol following a reported procedure [14]; but on a large scale we have observed that a partial loss of the optical purity of the product might easily occur during the reaction. Then, we tested three different reducing agents (methods A, B and C). Indeed, even if the reduction with an over stoichiometric amount of *L*-selectride at −78 °C in THF gave excellent results (method A) [9], since **4** was isolated in an 84% yield and with a de of 97% [9], this reagent is quite expensive hampering its utilization on a large scale. For this reason we tried the more convenient Meerwein–Ponndorf–Verley reduction [15] (DIBAL-H, iPrOH in toluene at room temperature), but both the yield and the selectivity did not result satisfactory (10% conversion, 0% de, method B). Undoubtedly, a catalytic and a more operationally simple procedure would be highly desirable, to this regard the enzymatic reduction of isopulegone was tested as well (method C). Thus, the biocatalysed reduction [16] of this ketone with a panel of commercially available alcohol dehydrogenases (ADHs) was screened [17], the regeneration of the NAD(P)^+^ cofactor was carried out using a glucose dehydrogenase (GDH from *Bacillus megaterium*), with glucose as co-substrate [18]. The product distributions are reported in Table 1. However, most of these ADHs gave low conversions and selectivity. The best performances were obtained with the *Thermoanaerobium brokii* ADH; but even if the de was excellent (>99%) the conversion was still too low (about 36% by GC) to be really exploited on a preparative scale.

**Table 1:** Alcohol dehydrogenase screening results, method C.

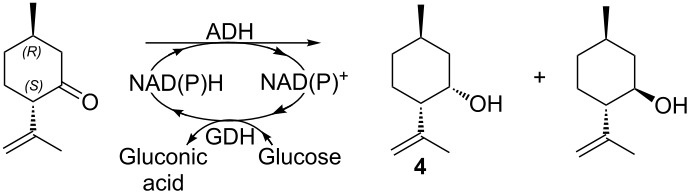					

ADH source	(*S*)-isopulegone [%]^a^	(−)-isopulegol [%]^a^	**4** [%]^a^	cofactor	de [%]^a^

*Candida parapsilosis*	100.0	0	0	NAD^+^	–
*Rhodococcus erythropolis*	98.5	0.5	1.0	NAD^+^	29.3
*Baker's yeast*	100	0	0	NAD^+^	–
*Horse liver recombinant*	100	0	0	NAD^+^	–
*Thermoanaerobium brockii*	63.7	0.1	**36.2**	NADP^+^	**99.7**
*Lactobacillus kefir*	90.5	9.5	0	NADP^+^	−99.9
*Parvibaculum lavamentivorans*	94.4	0.5	5.2	NADP^+^	82.5
*Deinococcus radiodurans*	100	0	0	NADP^+^	–
*Ketoreductase*	73.7	15.5	10.8	NADP^+^	−17.9

^a^By GC–MS.

Finally, we tried the Mitsunobu reaction, which gave the best results (method D) [19]. Indeed, the treatment of (–)-isopulegol with diisopropyl azodicarboxylate (DIAD) and *p*-nitrobenzoic acid in the presence of triphenylphosphine gave the corresponding ester in an almost quantitative yield. The latter was easily purified by crystallization from *n*-hexane, and then transesterificated with MeONa giving **4** in an overall yield of 84% and with an excellent de of >99%, by GC–MS ([α]_D_ +25° (*c* 2.0, CHCl_3_) vs lit. distomer (−)-neoisopulegol [19] [α]_D_ −22.2° (*c* 2.0, CHCl_3_) or (+)-neoisopulegol [20] [α]_D_ +28.7° (*c* 17.2, CHCl_3_)).

**Scheme 2:**
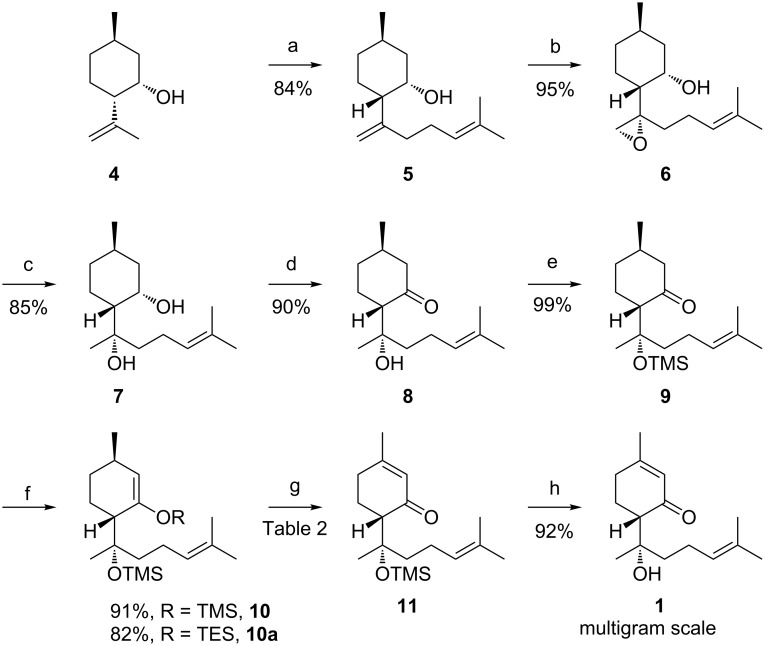
Reagents and conditions: (a) (i) *t*-BuOK, BuLi, hexane, −10 °C/rt; 2 h; (ii) BrCH_2_CH=C(CH_3_)_2_; −10°C/rt, 1 h; (b) TBHP, cat. VO(acac)_2_, toluene, rt, 13 h; (c) LiAlH_4_, THF, 0 °C, 5 h; (d) DMP, CH_2_Cl_2_, 0 °C/rt, 14 h; (e) TMSCl, CH_2_Cl_2_/pyridine (2:1), rt, 13 h; (f) LDA, TMSCl or TESCl, −78 °C/rt, THF; (g) see Table 2; (h) TBAF, MeCN, rt, 2 h.

The subsequent synthesis of **1** shown Scheme 2 is a slightly adapted version of the synthesis published in reference [9].

First, the metallation [21] of **4** with *t-*BuOK and *n*-BuLi in hexane followed by addition of prenyl bromide (3-methyl-2-butenyl bromide) at −10 °C afforded the sesquiterpene derivative **5** ([α]_D_ +19.5° (*c* 1.2, CHCl_3_)) in 84% yield. The VO(acac)_2_-catalyzed epoxidation of **5** with *tert*-butylhydroperoxide (TBHP) in toluene gave **6** ([α]_D_ +34.2° (*c* 1.3, CHCl_3_)) in 95% yield [9,22]. Then, the diol **7** was obtained in 85% yield from the reduction of epoxide **6** with LiAlH_4_ in THF at 0 °C. The diol **7** was easily purified by crystallization (85% yield, *n*-hexane at −50 °C up to −30 °C). Treatment of **7** with Dess–Martin periodinane (DMP) [23] in CH_2_Cl_2_ at room temperature gave ketone **8** ([α]_D_ +11.0° (*c* 1.0, CHCl_3_)) in 90% yield.

The most difficult step of this synthetic route is the dehydrogenation of ketone **8**. Since our main interest is focused on the taste evaluation of the final products, we deliberately avoided all toxic selenium based reagents [12–13]. We tested several methodologies and the results are summarized in Table 2.

**Table 2:** Investigated methodologies for preparation of **1**.

Substance	Conversion [%]^a^	Protocol

**8**	–	IBX, DMSO, 80 °C, 12 h^b^
**10**	trace	IBX, MPO, DMSO, rt, 12 h^b^
**10**	54	Pd(OAc)_2_, O_2_, DMSO, 58 °C, 3 d^b^
**10**	21	Pd(TFA)_2_, O_2_, Na_2_HPO_4_, MeCN, rt, 12 h
**10**	6	Pd(OAc)_2_, Oxone, Na_2_HPO_4_, MeCN, 50 °C, 38 h
**10a**	trace	Pd(TFA)_2_, O_2_, Na_2_HPO_4_, DMSO/CH_2_Cl_2_ 2:1, rt, 1 d
**10a**	4	Pd(OAc)_2_, O_2_, MeCN/CH_2_Cl_2_ 2:1, 50 °C, 7 d
**10a**	–	Pd(OAc)_2_, dppe, diallyl carbonate, MeCN/CH_2_Cl_2_ 2:1, 50 °C, 12 h

^a^By GC–MS; ^b^These methods have been previously tested [9].

In our initial synthesis [9] we applied the hyperiodine chemistry, developed by Nicolaou. However, the *o*-iodoxybenzoic acid (IBX) mediated oxidation of **8** to give the enone **1** in DMSO at high temperature (80 °C) resulted unsuccessful [24]. The hydrogenation of silyl enol ether derivatives in the presence of the IBX-*N*-oxide complex gives the corresponding enones, usually with better conversion and under milder conditions (room temperature) [25]. Thus, first the tertiary alcohol of **8** was protected as trimethylsilyl ether giving **9** ([α]_D_ −11.5° (*c* 1.3, CHCl_3_), vs lit. [13] [α]_D_ −16.3° (*c* 0.12, EtOH)), which, after treatment with lithium diisopropylamide (LDA) followed by addition of trimethylsilyl chloride (TMSCl) at –78 °C in THF afforded the kinetic enol ether **10** in 91% yield ([α]_D_ +19.7° (*c* 1.4, CHCl_3_)). The latter was submitted to the oxidative step with the IBX-*N*-oxide, but even in this case the results were unsatisfactory since only traces of enone **11** were detected. In contrast, the Pd based Saegusa–Larock methodology resulted successful [26–27], indeed when **10** was treated with a substoichiometric amount of Pd(OAc)_2_ in DMSO at 58 °C under an oxygen atmosphere, it was possible to isolate **11** ([α]_D_ +11.1° (*c* 1.4, CHCl_3_), vs lit. [13] [α]_D_ +9.7° (*c* 0.14, EtOH)) in a maximum yield of 54%, as no starting material was present anymore in the reaction mixture.

Very recently, Stahl et al. have proved that by replacing Pd(OAc)_2_ with Pd(TFA)_2_ it is possible to dehydrogenate directly the ketones at room temperature [28], but without the possibility of controlling the regioselectivity. Since, in our case this issue is critical we tried to apply the Pd(TFA)_2_ catalyzed dehydrogenation on the silyl enol ether **10** and in the presence of Na_2_HPO_4_ buffer, in order to mitigate the detrimental acidity of TFA. However, **11** was produced in a modest yield of 21%, because, even at these mild conditions, **10** reconverted to the initial ketone **9** faster than its oxidative dehydrogenation. Then we tested another procedure, in which the co-oxidant O_2_ was replaced with Oxone [29], but even in this case the conversion (6% by GC) was worse than that achieved using bubbling O_2_. Further attempts of optimizing the oxidative dehydrogenative step of the Stahl protocol were carried out by changing the trimethylsilyl enol ether group with the more robust triethylsilyl enol ether, **10a**. In principle this enolether should be more compatible with the Pd(TFA)_2_ catalyst, but unfortunately **10a** is insoluble in the typical solvents in which are carried out these Pd-catalyzed dehydrogenations (mainly DMSO or MeCN); even when using mixed co-solvent systems, the results were still very poor. Next, we tested the Tsuji variant [30] (Pd(OAc)_2_, dppe, diallyl carbonate, MeCN) but both **10** and **10a** decomposed during the reaction.

Finally, (+)-hernandulcin **1** was obtained in a 92% yield by cleavage of the silyl protective group of **11** with tetra-*n*-butylammonium fluoride (TBAF) in MeCN and at room temperature, the spectroscopic data of **1** were in complete agreement with those reported in literature [6] ([α]_D_ +130° (*c* 1.6, CHCl_3_) vs lit. [31] ([α]_D_ +115° (*c* 0.64, CHCl_3_)).

**Scheme 3:**
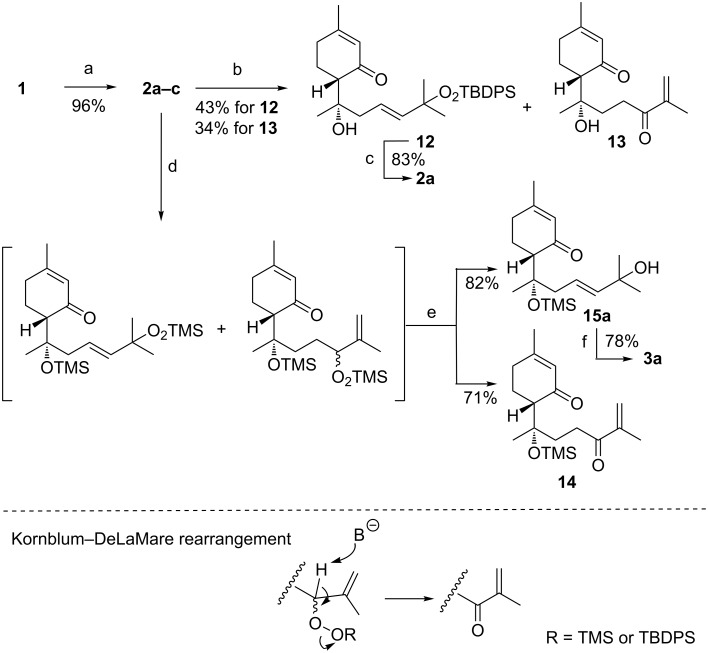
Reagents and conditions: (a) cat. methylene blue, light, bubbling O_2_, CH_2_Cl_2_/MeOH 4:1, rt, 15 h; (b) imidazole, TBDPSCl, cat. DMAP, DMF, rt, 24 h; (c) TBAF, THF, rt, 12 h; (d) TMSCl, cat. DMAP, CH_2_Cl_2_/pyridine 2:1, 0 °C/rt, 24 h; (e) acidic-aqueous work-up then PPh_3_, CH_2_Cl_2_, rt, 15 min, then MnO_2_, CH_2_Cl_2_, rt, 1 h, TBAF, 6.0 equiv H_2_O, MeCN, rt, 30 min.

The photooxygenation [32] of (+)-hernandulcin in a mixed solvent system (CH_2_Cl_2_/MeOH, 4:1) and in presence of a catalytic amount of the methylene blue photosensitizer (<0.1% in weight, λ = 280 nm) afforded a mixture of peroxylippidulcines A, B and C, i.e., **2a–c**, in the ratio of 47:21:32 (by ^1^H NMR) and in an almost quantitative yield (Scheme 3). However, any attempt of isolating each isomer of peroxylippidulcine by column chromatography technique failed. In addition, the singlet dark oxidation (H_2_O_2_ with a catalytic amount of Na_2_MoO_4_ at 55 °C) was tested, but without success [33].

Then, we tried to separate **2a–c** by means of a regioselective *O*-silylation of the secondary hydroperoxide with the *tert*-butyldiphenylsilyl chloride (TBDPSiCl), but with our surprise most of the starting material was consumed, and after column chromatography separation only the *O*-silylated derivative of peroxylippidulcine A, **12**, and a small amount of ketone **13** were isolated (Scheme 3). The latter is likely produced by a variant of the Kornblum–DeLaMare rearrangement [34–35] of the *O*-silyl precursor at room temperature, unfortunately **13** is very unstable. However, we envisaged the possibility of using this ketone, prior its tertiary alcohol protection, as a key-precursor for the preparation of lippidulcines B and C by means of a stereoselective and regioselective reduction of the exocyclic carbonyl group C(1). Indeed, the peroxy lippidulcines are not interesting from the sensorial point of view, since the taste of **2a** ([α]_D_ +43.5° (*c* 1.6, CHCl_3_) vs lit. [8] [α]_D_ +42.0° (*c* 3.2, CHCl_3_)), obtained by treatment of **12** with TBAF in MeCN, resulted very bitter, as usually are the hydroperoxides (Table 3).

Thus, the crude material of the photooxygenation was treated with an over stoichiometric amount of TMSCl (Scheme 3). In these conditions, after 24 hours most of peroxylippidulcine A was completely *O*-silylated, whereas the B and C ones rearranged partially to give the *O*-silyl protected ketone, **14** ([α]_D_ +13.6° (*c* 1.1, CHCl_3_). Since in this case, the Kornblum–DeLaMare rearrangement was not quantitative (around 68% by ^1^H NMR), after the aqueous work-up followed by treatment of the reaction mixture with triphenylphosphine, the unreacted lippidulcines B and C were oxidized with MnO_2_ to give ketone **14** together to the unreacted *O*-TMS protected lippidulcine A, **15a** ([α]_D_ −11.3° (*c* 1.0, CHCl_3_)). The latter were easily separated by column chromatography. Then, the *O*-silyl group of **15a** was cleaved with TBAF affording lippidulcine A ([α]_D_ +132° (*c* 1.3, CHCl_3_) vs lit. [8] [α]_D_ +123.6° (*c* 0.1, CHCl_3_)), which turned out to be a very pleasant cooling agent with a very light mint after taste (Table 3).

**Scheme 4:**
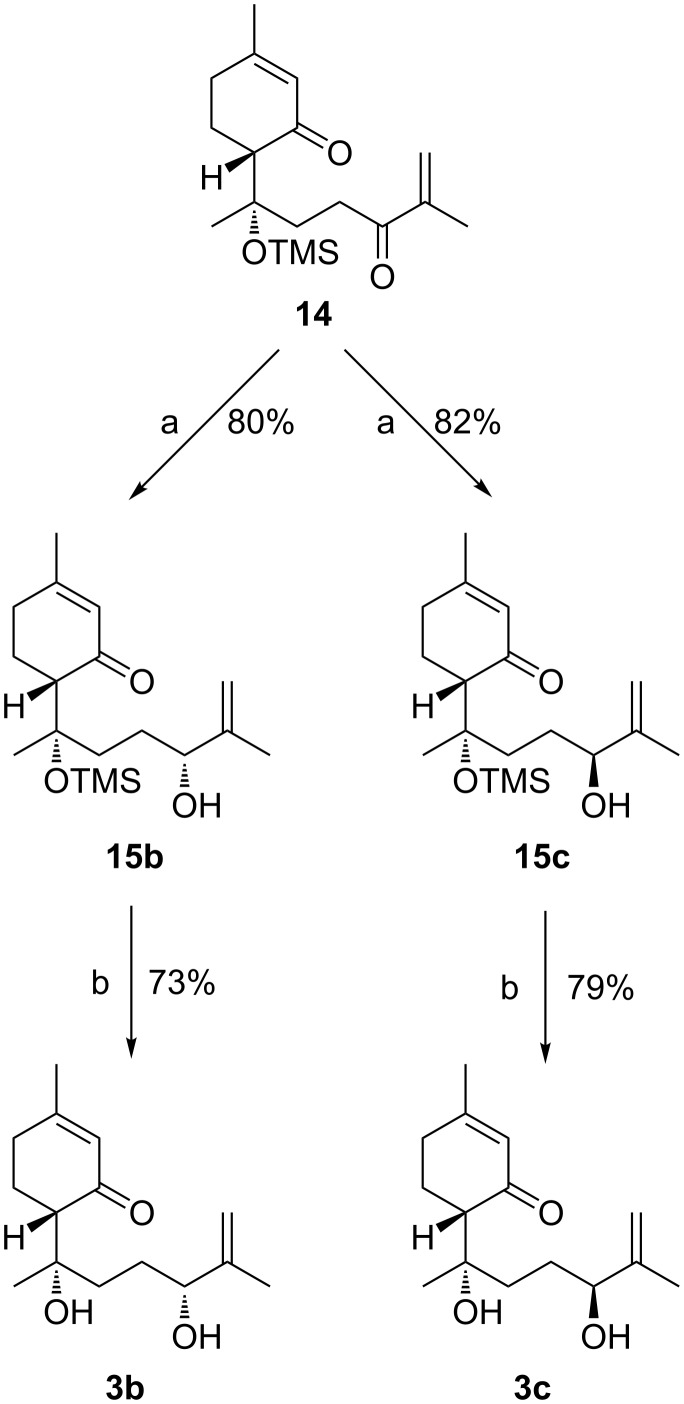
Reagents and conditions: (a) (*S*)-MeCBS or (*R*)-MeCBS for **15b** or **15c**, respectively, BH_3_·Me_2_S, −78 °C for 1 d then −60 °C for 2 d; (b) TBAF, MeCN, rt, 30 min.

Ketone **14** was submitted to the Corey–Bakshi–Shibata stereoselective carbonyl reduction protocol [36] using (*S*)-MeCBS as catalyst (1.2 equiv) in CH_2_Cl_2,_ and in the presence of an over stoichiometric amount of the Me_2_S^.^BH_3_ complex (Scheme 4). After column chromatography, the reduced product **15b** ([α]_D_ −1.6° (*c* 0.8, CHCl_3_)) was obtained in a good yield of 80%.

Thus, lippidulcine B (**3b**), after TBAF mediated cleavage of the silyl protecting group, was isolated in 73% yield and with an excellent de of 99% (by ^1^H NMR), ([α]_D_ +123° (*c* 1.5, CHCl_3_) vs lit. [8] [α]_D_ +113.3° (*c* 0.4, CHCl_3_)), confirming the absolute stereochemistry that it has been previously assigned. It is noteworthy that in this case the Corey asymmetric reduction of the carbonyl group is completely regioselective in favour of the exocyclic carbonyl group. To knowledge of the authors, just another example has been reported with a similar regioselectivity [37]. Then, lippidulcine C ([α]_D_ +92.1° (*c* 1.1, CHCl_3_) vs lit. [8] [α]_D_ +119.8° (*c* 0.7, CHCl_3_)) was prepared following the same route adopted for the synthesis of **3b**, but using the (*R*)-MeCBS.

Finally, the samples of lippidulcines were submitted to the taste evaluation, the results are summarized in Table 3.

**Table 3:** Sensorial evaluation of lippidulcines.

Substance	taste description^a^

**2a**	Very bitter with a light mint after taste
**3a**	Pleasant cooling agent, fresh sensation with a very slight mint after taste
**3b**	Fresh pungent then bitter
**3c**	Bitter
**3d**	Slightly fresh pungent, then very bitter

^a^The evaluation has been made on a panel of 4 people.

In summary, the introduction of an hydroxy group on the side chain of hernandulcin has changed drastically the taste of the latter, indeed the lippidulcines B and C are bitter, whereas, very surprisingly **3a** turned out to be a new natural cooling agent [38] with a light mint retro taste. These results are in contrast with the behavior of the (+)-β-hydroxyhernadulcin isomer (Figure 1), which has been described as a sweetener [39]. Intrigued by the behavior of **3a** we prepared the epimer **3d** ([α]_D_ −107° (c 1.2, CHCl_3_) vs lit. [8] [α]_D_ −118.4° (c 0.5, CHCl_3_)) by the acid catalyzed racemization of C(6) stereogenic center. Remarkably, the absolute configuration at this stereocenter plays in important role, since **3d** resulted pungent cool and bitter.

## Conclusion

We improved the synthesis of (+)-hernadulcin on a multigram scale, and we have accomplished the first total synthesis of peroxylippidulcine A and lippidulcines A, B and C, confirming their absolute stereochemical configurations that have been previously assigned. The key steps are: i) a modified version of the Kornblum–DeLaMare rearrangement, promoted by the *O*-silylation of the hydroperoxy group, and ii) an highly regioselective and stereoselective reduction of the exocyclic carbonyl group of ketone **14** with the Corey reagent. The taste evaluation of these bisabolanes has demonstrated that the insertion of a hydroxy group on the side chain of hernadulcin annuls its intense sweetness.

## Supporting Information

File 1 Experimental procedures, analytical data, and copies of ^1^H and ^13^C NMR spectra of all compounds.
