# Supplementary material for: Stereoselective synthesis of hernandulcin, peroxylippidulcine A, lippidulcines A, B and C and taste evaluation
Source: Beilstein J Org Chem. 2015 Nov 5;11:2117–24. doi: 10.3762/bjoc.11.228 (PMC4661015; doi:10.3762/bjoc.11.228)
Supplement: File 1 — Experimental procedures, analytical data, and copies of 1H and 13C NMR spectra of all compounds. [file Beilstein_J_Org_Chem-11-2117-s001.pdf]

Supporting Information

for

Stereoselective synthesis of hernandulcin, peroxylippidulcine A, lippidulcines A, B and C and taste evaluation

Marco G. Rigamonti and Francesco G. Gatti*

Chemistry Department "G. Natta", Politecnico di Milano, Piazza Leonardo da Vinci 1, 20133 Milano, Italy

Email: Francesco Gatti - Francesco.Gatti@polimi.it

*Corresponding author

Experimental procedures, analytical data, and copies of ^{1}H and ^{13}C NMR spectra of all compounds

Table of Contents

1. General information: S2

2. Experimental procedures and analytical data: S3 to S21

3. Sensorial evaluation criteria: S22

4. Notes and references: S22

5. Copies of ^{1}H and ^{13}C NMR spectra: S23 to S46

1. General Information

^{1}H and ^{13}C NMR spectra were recorded on a Bruker 400 or 500 DRX spectrometer in $CDCl_3$ solutions at room temperature using residual $CHCl_3$ or TMS as internal reference for ^{1}H and $CDCl_3$ for ^{13}C, chemical shifts are expressed in ppm and *J* values in Hz. GC–MS analyses were performed on an Agilent HP 6890 gas-chromatograph equipped with a 5973 mass detector and an Agilent HP-5 (30 m × 0.25 mm × 0.25 μm) column. Temperature program: 60 °C (1 min) / 6 °C min^{-1} / 150 °C (1 min) / 12 °C min^{-1} / 280 °C (5 min). Optical rotations were determined on a Dr. Kernchen Propol digital automatic polarimeter at 589 nm and are given in $°cm^3\ g^{-1}\ dm^{-1}$ at rt. Thin layer chromatography (TLC) analyses were performed on Merck Kieselgel 60 F_{254} plates, and column chromatographic separations were carried out on silica gel. All reagents and solvents were purchased from Sigma-Aldrich. The reagents were used without further purification, while where required the solvents were anhydrificated with molecular sieves. All the ADHs employed were purchased from Sigma-Aldrich except those from *C. parapsilosis* and *R. erythropolis* which were purchased from Jülich.

2. Experimental procedures and analytical data

(*S*)-Isopulegone

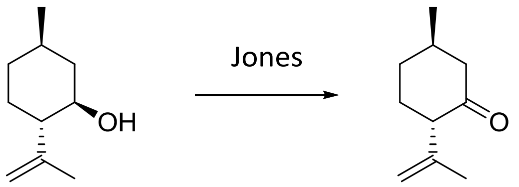

(-)-isopulegol **(*S*)-isopulegone**

To an ice cold and well stirred solution of (−)-isopulegol (31.0 g, 0.2 mol) in acetone (300 mL) was added dropwise the Jones reagent (2.8 M, 93.0 mL, 0.26 mol, 1.3 equiv). The reaction mixture was stirred for 3 h, until no further conversion was observed. Then, the reaction mixture was quenched with $NaHCO_3$ (1 M, 150 mL). The slurry was filtered on a celite pad; the latter was washed with EtOAc (3 × 50 mL). The filtrate was concentrated under reduced pressure and diluted with Et_2O (200 mL), washed with brine (3 × 50 mL), dried over Na_2SO_4 and the solvent was removed under reduced pressure to afford the crude product as a yellow-green liquid. Column chromatographic separation (eluent *n*-hexane/EtOAc, 95:05) gave (*S*)-isopulegone as colourless liquid (16.2 g); 53% yield; R_f =0.43 (9:1, *n*-hexane/EtOAc); t_r = 10.67 min 99% purity by GC; 95% de by NMR; $[\alpha]_D$ = –10.1° (*c* 2.0, $CHCl_3$); ^1H NMR (400 MHz, $CDCl_3$) δ 4.86 (quin, *J* = 1.5 Hz, 1H), 4.65 (sep, *J* = 1.7, 0.8 Hz, 1H), 2.89 (dd, *J* = 12.9, 5.4 Hz, 1H), 2.33 (ddd, *J* = 13.3, 3.9, 2.2 Hz, 1H), 2.03 – 1.94 (m, 2H), 1.91 – 1.76 (m, 2H), 1.73 (dd, *J* = 13.0, 3.4 Hz, 1H), 1.69 (dd, *J* = 1.4, 0.9 Hz, 3H), 1.37 (tdd, *J* = 12.5, 11.1, 3.3 Hz, 1H), 0.98 (d, *J* = 6.3 Hz, 3H); ^{13}C NMR (100 MHz, $CDCl_3$) δ 209.79, 143.48, 112.71, 57.69, 50.54, 35.27, 33.86, 31.21, 22.26, 21.26; GC/MS: *m/z* (%) 152 (M^+, 25), 137 (20), 67 (100).

(+)-Neoisopulegol (4)

Method A

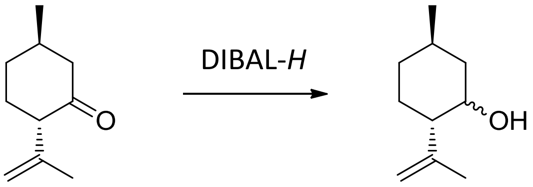

4

To an ice cold solution of DIBAL-*H* in THF (1.8 M, 5.5 mL, 10 mmol, 1 equiv) under a nitrogen atmosphere was added dropwisely a solution of 2-propanol (0.75 mL, 10

mmol, 1 equiv) in toluene (2.0 mL) and then left at rt for 1 h. Then, the reaction mixture was ice cooled and the (*S*)-isopulegone (1.5 g, 10 mmol) was slowly added, left at 0 °C for 3 h and then for 3 h at rt. 10% conversion and 0% de was obtained.

Method B

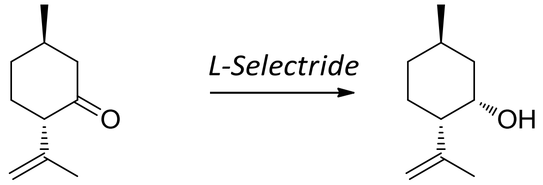

4

To a solution of (*S*)-Isopulegone (6.5 g, 42.7 mmol) in THF (130 mL) under a N_2 atmosphere at −78 °C was added dropwise a solution of *L*-Selectride in THF (1.0 M, 64.0 mL, 64.0 mmol, 1.5 equiv). The reaction temperature was left to reach rt in about 2 hours. After stirring for 14 h the mixture was cooled to 0 °C and H_2O_2 (30 wt %, 22.0 mL, 170 mmol, 4 equiv) was slowly added, followed by aqueous NaOH (15 mL, 15 wt.%) and stirred overnight at rt. The organic layer was washed with brine (3 × 30 mL), dried over Na_2SO_4 and the solvent removed under reduced pressure to afford a yellowish crude liquid. Column chromatographic separation (eluent *n*-hexane/EtOAc, 98:2) gave **4** as colourless liquid (5.5 g); 84% yield; R_f=0.52 (8:2 *n*-hexane/EtOAc); t_r = 10.21 min 99% by GC; 96.7% de by NMR; $[\alpha]_D$ = +24.5° (*c* 2.0, $CHCl_3$) *vs.* lit.[1] $[\alpha]_D$ = + 28.7° (*c* 17.2, $CHCl_3$) or distomer[2] $[\alpha]_D$ = –22.2° (*c* 2.0, $CHCl_3$); ^{1}H NMR (400 MHz, $CDCl_3$) δ 4.90 (dq, *J* = 2.1, 1.0 Hz, 1H), 4.75 (s, 1H), 3.95 (s, 1H), 1.98 – 1.88 (m, 2H), 1.84 – 1.62 (m, 6H), 1.52 (s, 1H), 1.46 – 1.37 (m, 1H), 1.14 – 1.04 (m, 1H), 0.97 – 0.88 (m, 1H), 0.85 (d, *J* = 6.5 Hz, 3H); ^{13}C NMR (100 MHz, $CDCl_3$) δ 147.42, 111.22, 66.50, 48.58, 41.20, 34.92, 25.86, 24.07, 22.74, 22.28; GC/MS: *m/z* (%) 154 (M^+, 15), 136 (50), 121 (100). Anal. Calcd for $C_{10}H_{18}O$: C, 77.87; H, 11.76. Found: C, 77.84; H, 11.72.

Method C

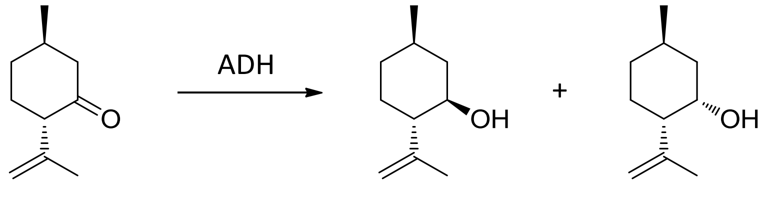

To a phosphate buffer solution (50 mM, 850 μL, pH 7) were added (*S*)-isopulegone (770 μg, 5 μmol) in DMSO (10 μL), glucose (20 μL, 1 M sol), $NAD(P)^+$ (10 μL, 10 mM sol., see Table 1), the ADH (see Table 1), the GDH-BM (30 μL, 1.0 mg/mL sol.). The reaction was stirred for 24 h at 30 °C, then extracted with EtOAc (1.0 mL), dried over Na_2SO_4 and submitted to GC–MS analysis (Table 1).

Method D

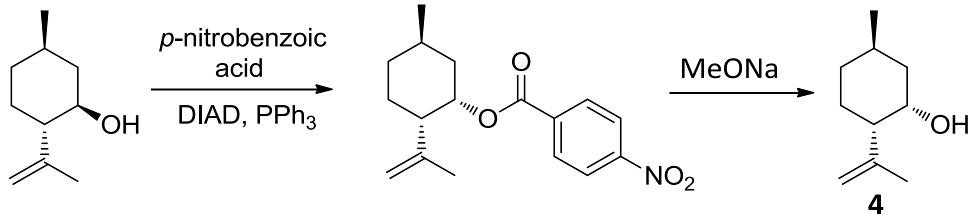

To an ice cold mixture and well stirred mixture of (−)-isopulegol (24.0 g, 156 mmol), *p*-nitrobenzoic acid (52.0 g, 0.31 mol, 2 equiv) and PPh_3 (82.0 g, 0.31 mol, 2 equiv) in toluene (160 mL) was added dropwise DIAD (64.0 g, 312 mmol, 2 equiv). The reaction mixture was stirred at rt for 24 h, then it was filtered at -10 °C and the solid was washed with *n*-hexane (3 × 50 mL). The organic phase was extracted with brine (3 × 100 mL). The combined aqueous layers were extracted with Et_2O (3 × 50 mL). The combined organic layers were dried over Na_2SO_4 and the solvent removed under reduced pressure to afford a yellowish crude solid. Crystallization of PPh_3O, with *n*-hexane (100 mL), at 0 °C, allowed the purification of crude ester which was used for the next step without further purification; 87% yield; t_r = 26.22 min 91% purity by GC; $[\alpha]_D$ = +37.6° (*c* 1.4, $CHCl_3$); ^1H NMR (400 MHz, $CDCl_3$) δ 8.34 – 8.10 (m, 4H), 5.56 (dd, *J* = 3.7, 2.3 Hz, 1H), 4.83 – 4.64 (m, 2H), 2.18 – 2.04 (m, 2H), 1.98 – 1.85 (m, 2H), 1.83 – 1.69 (m, 5H), 1.36 – 1.24 (m, 1H), 1.17 – 1.02 (m, 1H), 0.93 (d, *J* = 6.6 Hz, 3H); ^{13}C NMR (125 MHz, $CDCl_3$) δ 164.01, 150.47, 145.93, 136.43, 130.73, 123.65, 111.20, 72.38, 47.00, 39.27, 34.58, 27.11, 25.40, 22.64, 22.23;

GC/MS: *m/z* (%) 303 (M^+, 1), 167 (8), 150 (63), 79 (100). Anal. Calcd for $C_{17}H_{21}NO_4$: C, 67.31; H, 6.98. Found: C, 67.32; H, 6.92.

To a water cooled and well stirred solution of the above ester (45.4 g, 136 mmol) in MeOH (200 mL) was added slowly a MeONa solution (0.68 M, 200 mL, 136 mmol, 3 equiv) over 1 h. Then the reaction mixture was allowed to reach rt and stirred for other 4 h. The reaction was concentrated under reduced pressure (100 mL), then it was added ice cold brine (400 mL). The mixture was filtered and the solid washed with MeOH (3 × 50 mL). The filtrate was washed with CH_2Cl_2 (3 × 200 mL) and the combined organic layers were washed with brine (2 × 100 mL), dried over Na_2SO_4 and the solvent was removed under reduced pressure to afford a yellowish liquid. Column chromatographic separation (eluent *n*-hexane/EtOAc, 95:5) gave **4** as colourless liquid (20.1 g); 96% yield; 97.5% purity by GC; >99% *de* by NMR and GC; $[\alpha]_D$ = +25° (*c* 2.0, $CHCl_3$) *vs.* lit.[1] $[\alpha]_D$ = + 28.7° (*c* 17.2, $CHCl_3$) or distomer[2] $[\alpha]_D$ = –22.2° (*c* 2.0, $CHCl_3$)**.**

(*1S,2S,5R*)-5-Methyl-2-(6-methylhepta-1,5-dien-2-yl)cyclohexanol (5)

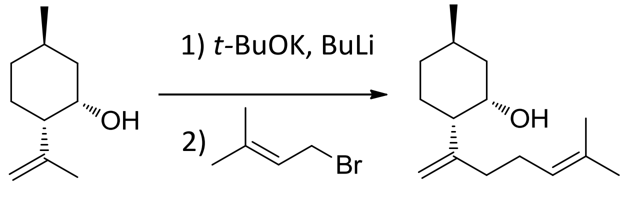

To an ice cooled solution of **4** (19.0 g, 123 mmol) in *n*-hexane (400 mL) under a N_2 atmosphere was quickly added *t*-BuOK (13.8 g, 123 mmol, 1 equiv) and left to stir at rt for 15 min. Therefore *n*-BuLi (2.5 M, 150 mL, 370 mmol, 3 equiv) was added dropwise at –10 °C. After 2 h freshly-clear-distilled prenyl bromide (17.8 mL, 154 mmol, 1.25 equiv) was added drop by drop, and the reaction was left to stir for 45 min at −10 °C, then at rt for 15 min. The reaction was quenched at 0 °C with brine (300 mL, starting drop by drop until no more gases are generated) until all solid were dissolved. Then the organic layer was separated and the aqueous layer was extracted with Et_2O (3 × 100 mL). The combined organic layers were washed with brine (2 × 100 mL) and the brine extracted with Et_2O (50 mL). Finally, the combined organic layers were dried over Na_2SO_4 and the solvent removed under reduced pressure. Column chromatographic separation (eluent *n*-hexane/EtOAc in gradient, from *n*-hexane to 98:2) gave **5** as colourless liquid (23.0 g); 84% yield; t_r= 20.10 min

96% purity by GC; R_f=0.5 (9:1, *n*-hexane/EtOAc); 96% *de* by NMR; $[\alpha]_D$ = +19.5° (*c* 1.2, $CHCl_3$); ^{1}H NMR (400 MHz, $CDCl_3$) δ 5.09 (tdq, *J* = 5.8, 3.0, 1.5 Hz, 1H), 4.94 (t, *J* = 1.5 Hz, 1H), 4.83 (s, 1H), 3.91 (q, *J* = 2.8 Hz, 1H), 2.21 – 1.91 (m, 6H), 1.86 – 1.69 (m, 3H), 1.67 (d, *J* = 1.4 Hz, 3H), 1.60 (d, *J* = 1.3 Hz, 3H), 1.57 – 1.51 (m, 1H), 1.45 – 1.34 (m, 1H), 1.10 (dddd, *J* = 13.6, 12.2, 2.7, 1.4 Hz, 1H), 1.04 – 0.90 (m, 1H), 0.87 (d, *J* = 6.5 Hz, 3H); ^{13}C NMR (100 MHz, $CDCl_3$) δ 151.43, 131.92, 124.00, 110.45, 66.45, 47.46, 41.13, 36.04, 35.09, 26.85, 25.91, 25.73, 24.35, 22.34, 17.80; GC/MS: *m/z* (%) 222 (M^+, 5), 204 (22), 161 (75), 69 (100); Anal. Calcd for $C_{15}H_{26}O$: C, 81.02; H, 11.79. Found: C, 81.07; H, 11.75.

(*1S,2R,5R*)-5-Methyl-2-((*S*)-2-(4-methylpent-3-enyl)oxiran-2-yl)cyclohexanol (6)

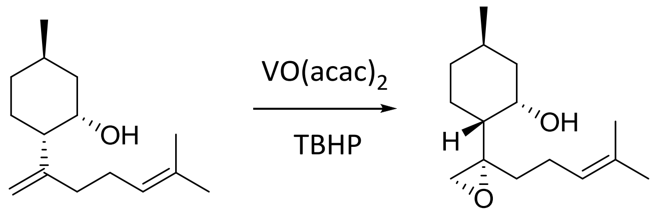

5 **6**

To a water cold solution of **5** (13.7 g, 61.7 mmol) and $VO(acac)_2$ (300 mg) in toluene (300 mL) under a N_2 atmosphere was added dropwise a solution of TBHP in toluene (4.87 *m*, 15.2 g, 74 mmol, 1.2 equiv) over 30 min. After 12 h was added PPh_3 (3.2 g, 12.3 mmol, 0.2 equiv), and after 5 minutes the solvent was removed under reduced pressure. Column chromatographic purification (eluent *n*-hexane/EtOAc gradient, *n*-hexane up to 95:5) gave **6** as colourless liquid (13.9 g); 95% yield; t_r = 22.00 min 96% purity by GC; R_f=0.32 (8:2, *n*-hexane/EtOAc); 95% *de* by NMR; $[\alpha]_D$ = +34.2° (*c* 1.3, $CHCl_3$); ^{1}H NMR (400 MHz, $CDCl_3$) δ 5.13 – 5.01 (m, 1H), 4.33 (s, 1H), 2.91 (s, 1H), 2.84 – 2.44 (m, 2H), 2.06 – 1.78 (m, 5H), 1.77 – 1.70 (m, 2H), 1.69 (dd, *J* = 5.9, 1.6 Hz, 3H), 1.60 (s, 3H), 1.48 (dddd, *J* = 20.0, 15.8, 13.2, 5.6 Hz, 2H), 1.38 – 1.23 (m, 2H), 1.08 – 0.90 (m, 2H), 0.86 (d, *J* = 6.5 Hz, 3H); ^{13}C NMR (125 MHz, $CDCl_3$) δ 132.38, 123.46, 67.89, 63.32, 49.61, 41.92, 41.56, 35.68, 34.88, 25.78, 25.67, 23.75, 22.93, 22.33, 17.80; GC/MS: *m/z* (%) 238 (M^+, 1), 220 (22), 95 (78), 69 (100); Anal. Calcd for $C_{15}H_{26}O_2$: C, 75.58; H, 10.99. Found: C, 75.57; H, 11.01.

(*1S,2R,5R*)-2-((*S*)-2-Hydroxy-6-methylhept-5-en-2-yl)-5-methylcyclohexanol (7)

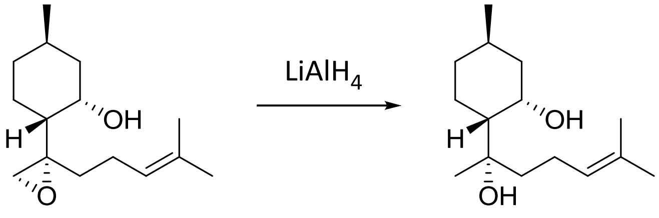

To an ice cold and well stirred solution of **6** (15.4 g, 64.5 mmol) in dry THF (250 mL) was added dropwise under a N_2 atmosphere a solution of $LiAlH_4$ (2.8 g, 71.5 mmol, 1.1 equiv) in dry THF (100 mL) over 45 min. The reaction mixture was stirred until no further conversion was observed after 4 h. Then, the slurry was quenched with a Signette salt solution (sat., 350 mL), filtered and the solid washed with CH_2Cl_2 (3 × 100 mL). The aqueous phase was washed with CH_2Cl_2 (3 × 100 mL). The combined organic phase was washed with the Signette salt solution (sat., 100 mL), brine (sat., 2 × 100 mL) and dried over Na_2SO_4. The solvent was removed under reduced pressure to give a yellow crude liquid. After crystallization (*n*-hexane, −70 °C to −30 °C) **7** was afforded as white solid (13.1 g); 85% yield; t_r = 22.59 min purity >99% by GC; R_f=0.19 (9:1, *n*-hexane/EtOAc); 96% de by NMR; $[\alpha]_D$ = +7.28° (*c* 1.2, $CHCl_3$); ^{1}H NMR (400 MHz, $CDCl_3$) δ 5.14 (tdq, *J* = 7.1, 2.9, 1.4 Hz, 1H), 4.33 (s, 1H), 3.40 (d, *J* = 2.3 Hz, 1H), 3.05 (s, 1H), 1.95 (ddq, *J* = 19.4, 14.1, 7.5, 7.0 Hz, 2H), 1.78 (ddq, *J* = 18.1, 9.7, 5.1, 4.2 Hz, 4H), 1.60 (d, *J* = 13.0 Hz, 9H), 1.20 (dt, *J* = 12.4, 3.0 Hz, 1H), 1.14 (s, 3H), 0.98 (d, *J* = 15.0 Hz, 1H), 0.87 (td, *J* = 12.9, 3.7 Hz, 1H), 0.84 (d, *J* = 6.2 Hz, 3H). ^{13}C NMR (100 MHz, $CDCl_3$) δ 131.57, 124.53, 75.28, 67.73, 46.24, 42.71, 41.31, 35.17, 25.75, 25.71, 25.30, 23.37, 22.27, 20.29, 17.70. GC/MS: *m/z* (%) 222 (M^+-18, 18), 204 (50), 189 (40), 109 (100). Anal. Calcd for $C_{15}H_{28}O_2$: C, 74.95; H, 11.74. Found: C, 74.90; H, 11.72.

(2*S*,5*R*)-2-((*S*)-2-Hydroxy-6-methylhept-5-en-2-yl)-5-methylcyclohexanone (8)

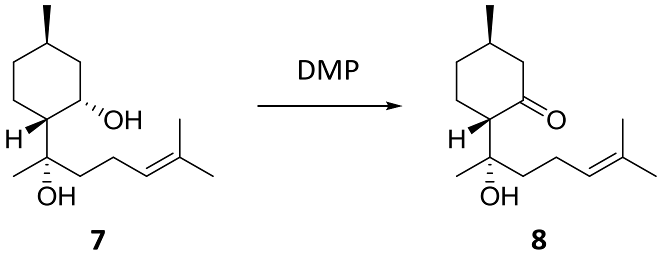

To an ice cold and stirred solution of **7** (1.00 g, 4.16 mmol) in CH_2Cl_2 (20 mL) was added portion wise DMP (2.12 g, 5 mmol, 1.2 equiv). After 1h the reaction was left to stir overnight at rt. Then it was added *n*-hexane/Et_2O (1:1, 40 mL), the slurry was filtrated and the filtrate was washed with $NaHCO_3$ (sat., 2 × 10 mL) and brine (sat., 10 mL), and finally dried over Na_2SO_4. The solvent was removed under reduced pressure. Column chromatographic separation (eluent, *n*-hexane/EtOAc gradient: *n*-hexane up to 8:2) gave **8** as a colourless liquid (0.88 g); 90% yield; R_f=0.55 (7:3, *n*-hexane/EtOAc); **8** decomposes into GC column into 3-methylcyclohexanone and 6-methyl-5-hepten-2-one; 96.3% de by NMR; $[\alpha]_D$ = +11.0° (*c* 1.0, $CHCl_3$); ^1H NMR (400 MHz, $CDCl_3$) δ 5.06 (dddd, *J* = 8.6, 5.7, 2.9, 1.5 Hz, 1H), 3.92 (s, 1H), 2.44 – 2.27 (m, 2H), 2.12 – 1.75 (m, 6H), 1.64 (q, *J* = 1.3 Hz, 3H), 1.58 (d, *J* = 1.3 Hz, 3H), 1.55 – 1.39 (m, 3H), 1.31 (tdd, *J* = 12.9, 11.2, 3.5 Hz, 1H), 1.14 (s, 3H), 0.99 (d, *J* = 6.2 Hz, 3H); ^{13}C NMR (100 MHz, $CDCl_3$) δ 215.29, 131.43, 124.68, 73.02, 56.87, 51.63, 40.56, 35.43, 34.07, 28.51, 25.72, 23.79, 22.29, 22.07, 17.66; Anal. Calcd for $C_{15}H_{26}O_2$: C, 75.58; H, 10.99. Found: C, 75.61; H, 10.94.

(2*S*,5*R*)-5-Methyl-2-((*S*)-6-methyl-2-(trimethylsilyloxy)hept-5-en-2-yl)cyclohexanone (9)

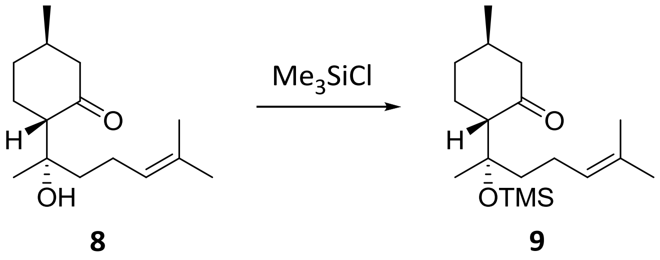

To a water cold and stirred solution of **8** (3.14 g, 13.2 mmol) in CH_2Cl_2/pyridine (2:1, 30 mL) was added dropwise TMSCl (3.6 g, 33 mmol, 2.5 equiv) under a N_2 atmosphere. After 13 h, to the reaction mixture at 0 °C was added drop by drop brine (sat., 50 mL). Then the slurry was filtered, the solid washed with CH_2Cl_2 (2 × 20 mL)

and the aq. phase extracted with CH_2Cl_2 (3 × 50 mL). The combined organic phase was dried over Na_2SO_4. The solvent was removed under reduced pressure. Column chromatographic separation (eluent *n*-hexane/EtOAc +1% Et_3N, gradient: *n*-hexane to 95:5) gave **9** as colourless liquid (4.05 g); 98.9% yield; R_f=0.75 (7:3 *n*-hexane/EtOAc); t_r = 22.49 min 96.2% purity by GC; $[\alpha]_D$ = -11.5° (*c* 1.3, $CHCl_3$) *vs.* lit.[3] $[\alpha]_D$ = –16.3° (*c* 0.12, EtOH); ^{1}H NMR (400 MHz, $CDCl_3$) δ 5.10 (ddq, *J* = 8.6, 5.8, 1.5 Hz, 1H), 2.39 (ddd, *J* = 12.7, 5.0, 1.2 Hz, 1H), 2.30 (ddd, *J* = 12.5, 4.3, 2.1 Hz, 1H), 2.20 (ddt, *J* = 13.2, 5.0, 3.4 Hz, 1H), 2.06 – 1.80 (m, 5H), 1.74 – 1.64 (m, 4H), 1.63 – 1.59 (m, 3H), 1.52 (qd, *J* = 12.8, 3.2 Hz, 1H), 1.44 – 1.22 (m, 5H), 0.99 (d, *J* = 6.3 Hz, 3H), 0.10 (s, 9H). ^{13}C NMR (100 MHz, $CDCl_3$) δ 210.74, 131.02, 125.23, 76.66, 60.66, 52.14, 38.70, 35.98, 34.49, 27.86, 25.87, 25.79, 22.56, 22.38, 17.69, 2.72; GC/MS: *m/z* (%) 220 (M^+-H2OTMS, 32), 295 (M^+-Me, 5), 185 (38), 109 (100). Anal. Calcd for $C_{18}H_{34}O_2Si$: C, 69.62; H, 11.04. Found: C, 69.61; H, 11.07.

General procedure for the preparation of silylenolether 10a and 10

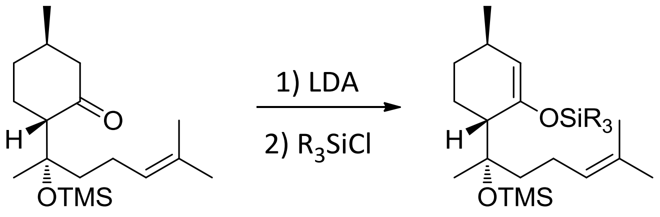

9

R=Me,**10**
R=Et, **10a**

To a stirred solution of diisopropylamine (1.62 g, 16 mmol, 1.6 equiv) in dry THF (10 mL) at −30 °C was added dropwise *n*-BuLi (2.5 M, 5.6 mL, 14 mmol, 1.4 equiv) under a N_2 atmosphere keeping the temperature below −25° C. The reaction was left at rt for 10 min. To the reaction mixture at -78 °C was added dropwise a solution of **9** (3.11 g, 10 mmol) in dry THF (10 mL) keeping the temperature below -73 °C during the addition. After 90 min at -78 °C. a solution of R_3SiCl (13 mmol, 1.3 equiv) in dry THF (10 mL) was added dropwisely in such a way to keep the temperature below -73 °C. After 90 min at -78 °C the reaction mixture was left to reach rt. Then, at -30 °C a phosphate buffer solution (1 M, 30 mL, 7.1 pH) was added. Then, it was filtrated on celite pad, the solid was washed with CH_2Cl_2 (2 × 10 mL). The aq. phase was washed with CH_2Cl_2 (3 × 30 mL). The combined organic phase was dried over Na_2SO_4. The solvent was removed under reduced pressure to afford a yellow liquid.

Trimethyl((*S*)-6-methyl-2-((*1S,4R*)-4-methyl-2-(trimethylsilyloxy)cyclohex-2-enyl)hept-5-en-2-yloxy)silane (10)

In this case the product as yellow liquid (3.49 g) was of sufficient purity for the next step; 91% yield; R_f=0.68 (8:2, *n*-hexane/EtOAc); t_r = 22.95 min 95% purity by GC; 95% de; $[\alpha]_D$ = +19.7° (*c* 1.4, $CHCl_3$); ^1H NMR (400 MHz, $CDCl_3$) δ 5.12 (tp, *J* = 7.1, 1.5 Hz, 1H), 4.70 (p, *J* = 1.0 Hz, 1H), 2.26 (dddd, *J* = 9.7, 6.6, 3.1, 1.4 Hz, 1H), 2.15 (ddtd, *J* = 11.4, 9.5, 4.8, 2.6 Hz, 1H), 2.07 – 1.94 (m, 2H), 1.88 – 1.71 (m, 2H), 1.68 (q, *J* = 1.2 Hz, 3H), 1.61 (d, *J* = 1.2 Hz, 3H), 1.57 – 1.26 (m, 7H), 0.92 (d, *J* = 6.9 Hz, 3H), 0.19 (s, 9H), 0.11 (s, 9H). ^{13}C NMR (100 MHz, $CDCl_3$) δ 151.03, 130.56, 125.52, 113.94, 78.37, 49.99, 38.02, 31.68, 30.21, 29.07, 25.66, 25.17, 22.77, 22.69, 17.55, 2.58, 0.27. GC/MS: *m/z* (%)382 (M^+-1, 1), 367 (M^+-Me, 2), 69 (40), 199 (100). Anal. Calcd for $C_{21}H_{42}O_2Si_2$: C, 65.90; H, 11.06. Found: C, 65.91; H, 11.04.

Triethyl((*3R,6S*)-3-methyl-6-((*S*)-6-methyl-2-(trimethylsilyloxy)hept-5-en-2-yl)cyclohex-1-enyloxy)silane (10a)

Column chromatographic separation (eluent *n*-hexane (+1% Et_3N)) gave **10a** as colourless liquid 3.48 g; 82% yield; R_f=0.62 (9:1, *n*-hexane/EtOAc); t_r = 25.46 min 96.1% purity by GC; $[\alpha]_D$ = +16.6° (*c* 1.2, $CHCl_3$); ^1H NMR (400 MHz, $CDCl_3$) δ 5.12 (dddd, *J* = 8.6, 5.7, 2.8, 1.4 Hz, 1H), 4.70 (dt, *J* = 2.1, 1.0 Hz, 1H), 2.29 (dddd, *J* = 9.6, 6.8, 3.0, 1.4 Hz, 1H), 2.25 – 2.11 (m, 1H), 2.11 – 1.95 (m, 2H), 1.88 – 1.71 (m, 2H), 1.68 (q, *J* = 1.3 Hz, 3H), 1.61 (d, *J* = 1.2 Hz, 3H), 1.55 – 1.47 (m, 2H), 1.45 (s, 3H), 0.98 (q, *J* = 7.8 Hz, 11H), 0.92 (d, *J* = 6.9 Hz, 3H), 0.75 – 0.67 (m, 6H), 0.12 (s, 9H). ^{13}C NMR (100 MHz, $CDCl_3$) δ 151.46, 130.68, 125.82, 113.02, 78.72, 50.38, 38.10, 31.82, 30.43, 29.51, 25.82, 25.32, 22.98, 22.94, 17.69, 6.96, 5.32, 2.75. GC/MS: *m/z* (%) 409 (M^+-Me, 1), 334 (2), 69 (40), 199 (100). Anal. Calcd for $C_{24}H_{48}O_2Si_2$: C, 67.86; H, 11.39. Found: C, 67.84; H, 11.43.

(*S*)-3-Methyl-6-((*S*)-6-methyl-2-((trimethylsilyl)oxy)hept-5-en-2-yl)cyclohex-2-en-1-one (11)

Method A: Saegusa–Ito oxidation of 10

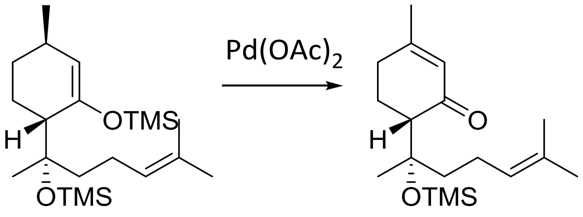

10 **11**

A mixture of $Pd(OAc)_2$ (700 mg, 3.13 mmol, 0.3 equiv) in dry DMSO (120 mL) was heated at 58 °C and stirred for 30 min in a Schlenk tube. Then, **10** (4.0 g, 10.4 mmol) was added, and O_2 was bubbled into the apparatus, which was shielded against sunlight. After 24 h it was added more catalyst (470 mg, 2.1 mmol, 0.2 equiv) and the reaction was bubbled for additional 2 days. Then, at rt was added a solvent mixture of *n*-hexane/Et_2O (3:7, 120 mL). The reaction mixture was filtrated on a pad of celite, the solid was washed with Et_2O (3 × 40 mL), the filtrate was washed with brine (sat., 200 mL). The aqueous phase was washed with *n*-hexane/Et_2O (3:7, 3 × 100 mL), the combined organic phase was washed with brine (sat., 4 × 30 mL) and dried over Na_2SO_4. The solvent was removed under reduced pressure to afford a dark crude oil. Column chromatographic separation (eluent *n*-hexane/EtOAc, 9:1 +0.1% Et_3N) gave **11** as a colourless liquid (1.74 g); 54% yield; t_r = 23.41 min 96% purity by GC; R_f=0.40 (9:1 *n*-hexane/EtOAc); $[\alpha]_D$ = +11.1° (*c* 1.4, $CHCl_3$) *vs.* lit.[3] $[\alpha]_D$ = +9.7° (*c* 0.14, EtOH)); ^1H NMR (400 MHz, $CDCl_3$) δ 5.76 (h, *J* = 1.3 Hz, 1H), 5.08 (ddq, *J* = 8.5, 5.8, 1.4 Hz, 1H), 2.45 – 2.24 (m, 2H), 2.23 – 2.12 (m, 2H), 2.11 – 2.00 (m, 1H), 2.00 – 1.89 (m, 2H), 1.88 (d, *J* = 1.3 Hz, 3H), 1.73 – 1.66 & 1.43 – 1.34 (ddd, 2H), 1.64 (q, *J* = 1.2 Hz, 3H), 1.59 (d, *J* = 1.3 Hz, 3H), 1.44 (s, 3H), 0.07 (s, 9H); ^{13}C NMR (100 MHz, $CDCl_3$) δ 199.90, 160.42, 131.04, 128.20, 124.99, 77.83, 54.83, 39.50, 31.14, 27.39, 25.71, 24.28, 23.84, 23.32, 17.66, 2.66; GC/MS: *m/z* (%) 308 (M^+, 1), 293 (M^+-Me, 15), 225 (63), 109 (100); Anal. Calcd for $C_{18}H_{32}O_2Si$: 70.07; H, 10.45. Found: 70.09; H, 10.48.

Method B: Saegusa–Ito oxidation of 10a

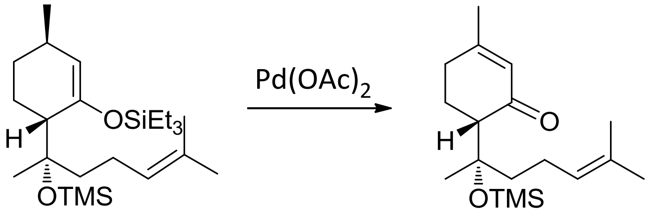

10a **11**

A mixture of **10a** (1.75 g, 4.12 mmol) and $Pd(OAc)_2$ (277 mg, 1.24 mmol, 0.3 equiv) in a mixed solvent system of dry DMSO/MeCN/CH_2Cl_2 (2:2:1, 250 mL) at 50 °C was bubbled with O_2 for 7 days in a Schlenk tube. During this time it was added occasionally more catalyst and CH_2Cl_2. Then, the work-up was made as in the previous case. 4% yield; the spectroscopic and GC data are consistent with that obtained with Method A.

(+)-Hernandulcin (1)

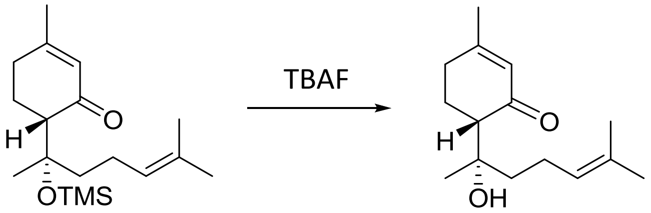

11 **1**

To a water cold and stirred solution of **11** (308 mg, 1 mmol) in MeCN (5 mL) was added TBAF (1 M, 2 mL, 2 mmol, 2 equiv) and the reaction was stirred at rt for 2 h. The reaction mixture diluted with Et_2O (20 mL), washed with brine (2 × 10 mL), dried over Na_2SO_4 and the solvent was removed under reduced pressure. Column chromatographic separation (eluent *n*-hexane/EtOAc gradient: *n*-hexane up to 9:1) gave **1** as an slight orange-yellow liquid (220 mg); 92% yield; R_f=0.52 (1:1 *n*-hexane/EtOAc); **1** decomposes into the GC-MS[4] in 3-methyl-2-cyclohexen-1-one and 6-methyl-5-hepten-2-one; $[\alpha]_D$ = +130° (*c* 1.6, $CHCl_3$) vs. lit.[5] ($[\alpha]_D$ = +115° (*c* 0.64, $CHCl_3$); ^{1}H NMR (400 MHz, $CDCl_3$) δ 5.83 (dq, *J* = 3.1, 1.4 Hz, 1H), 5.13 (t, *J* = 1.4 Hz, 1H), 5.08 (tdq, *J* = 7.1, 2.9, 1.4 Hz, 1H), 2.43 – 2.20 (m, 3H), 2.19 – 2.07 (m, 1H), 2.07 – 1.95 (m, 2H), 1.92 (t, *J* = 1.2 Hz, 3H), 1.72 – 1.61 (m, 1H), 1.64 (d, *J* = 1.3 Hz, 3H), 1.58 (d, *J* = 1.3 Hz, 3H), 1.44 (ddd, *J* = 9.0, 7.7, 1.3 Hz, 2H), 1.13 (s, 3H); ^{13}C NMR (100 MHz, $CDCl_3$) δ 203.95, 163.28, 131.39, 127.64, 124.71, 73.93, 52.38,

40.33, 31.40, 25.69, 25.20, 24.03, 23.70, 21.69, 17.66; Anal. Calcd for $C_{15}H_{24}O_2$: C, 76.23; H, 10.24. Found: C, 76.25; H, 10.22.

Peroxylippidulcines A, B and C (2a–c)

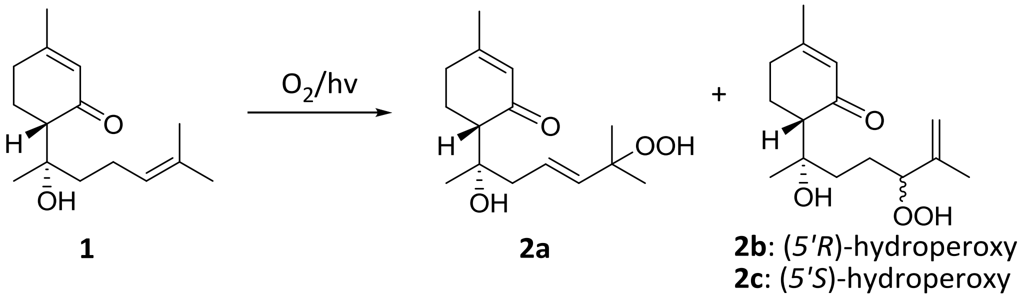

To a solution of **1** (1.1 g, 4.66 mmol) in CH_2Cl_2 (400 mL) and MeOH (100 mL) was bubbled O_2 in presence of methylene blue (3 drops, alcoholic sat. solution) and the reaction vessel was irradiated with white light for 15 h. Then, the solvent was removed under reduced pressure. The crude material was diluted with Et_2O/*n*-hexane (1:1, 200 mL), washed with brine (sat., 10 × 5 mL), dried over Na_2SO_4 and the solvent was removed under reduced pressure to afford a yellowish liquid **2** (1.2 g). 96% yield; R_f=0.13 (7:3 *n*-hexane/EtOAc); $[\alpha]_D$ of mixture = +90.9° (*c* 1.5, $CHCl_3$); **2a-c** decompose into the GC-MS[4];^{1}H NMR (400 MHz, $CDCl_3$) δ 9.47 (s, hydroperoxide **2b** 20.4%), 9.42 (s, hydroperoxide **2c** 32.3%), 8.83 (s, hydroperoxide **2a** 47.3%); ^{13}C NMR (100 MHz, $CDCl_3$) δ 204.27 (s, hydroperoxide **2a** 45%), 204.13 (s, hydroperoxide **2c** 34%), 204.03 (s, hydroperoxide **2b** 21%), 164.15, 164.10, 143.99, 137.25, 127.43, 127.39, 126.18, 113.55, 113.51, 89.06, 88.89, 81.81, 74.56, 74.34, 65.81, 52.50, 51.97, 51.89, 43.24, 35.57, 35.42, 31.31, 25.02, 24.96, 24.84, 24.54, 24.31, 24.16, 24.06, 23.82, 23.74, 23.26, 17.84, 15.18.

Photooxygenation followed by DeLaMare rearrangement

Method A (12 + 13)

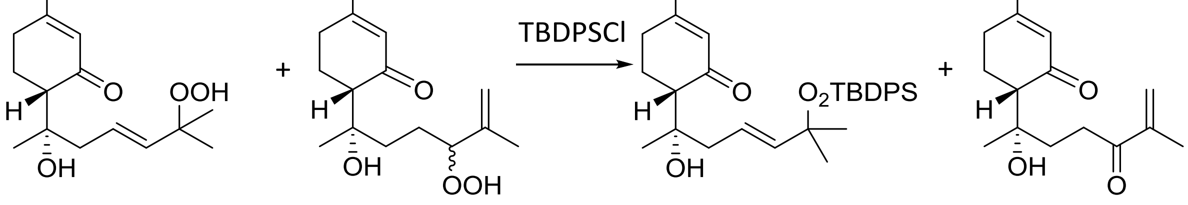

2a **2b-c** **12** **13**

To a stirred solution of **2** (0.9 g, 3.36 mmol) in dry DMF (15 mL) was added imidazole (460 mg, 6.7 mmol, 2 equiv), TBDPSCl (930 mg, 3.36 mmol, 1 equiv) and then a catalytic amount of DMAP (20 mg). Reaction was kept stirred at rt for 24 h, then the reaction mixture was ice-cooled and brine (sat., 15 mL) was added drop by drop. The reaction was diluted with *n*-hexane/Et_2O (7:3, 100 mL), extracted with brine (3 × 10 mL), HCl (1 M, 7 mL), brine (sat., 10 mL), $NaHCO_3$ (sat., 10 mL) and brine (sat., 10 mL). Finally, the combined organic phase was dried over Na_2SO_4. The solvent was removed under reduced pressure. Column chromatographic separation (eluent *n*-hexane/EtOAc in gradient: from *n*-hexane to 6:4) gave **12** as a yellowish solid (0.35 g mg) and **13** as a yellowish liquid (0.15 g).

(*S*)-6-((*S,E*)-6-(*tert*-Butyldiphenylsilylperoxy)-2-hydroxy-6-methylhept-4-en-2-yl)-3-methylcyclohex-2-enone (12). Yield 43%; R_f=0.34 (7:3, *n*-hexane/EtOAc); **12** decomposes into the GC-MS[4]; ^{1}H NMR (400 MHz, $CDCl_3$) δ 7.81 – 7.67 (m, 4H), 7.47 – 7.28 (m, 6H), 5.85 (ddd, J = 16.0, 9.1, 5.3 Hz, 1H), 5.79 (q, J = 1.5 Hz, 1H), 5.60 (ddd, J = 16.0, 1.8, 0.8 Hz, 1H), 2.35 (dd, J = 13.8, 4.5 Hz, 1H), 2.29 – 2.07 (m, 2H), 2.06 – 1.98 (m, 2H), 1.97 – 1.83 (m, 2H), 1.79 (d, J = 1.2 Hz, 3H), 1.74 – 1.37 (m, 3H), 1.32 (d, J = 8.3 Hz, 6H), 1.29 (dt, J = 2.8, 1.4 Hz, 3H), 1.16 (s, 3H), 1.12 (s, 9H), 0.94 – 0.86 (m, 3H). ^{13}C NMR (100 MHz, $CDCl_3$) δ 204.37, 163.58, 137.27, 135.91, 135.88, 134.97, 133.68, 129.78, 127.81, 127.56, 126.27, 82.94, 74.34, 51.60, 43.63, 31.70, 31.16, 27.56, 25.23, 24.87, 24.41, 24.18, 24.02, 22.76, 19.75, 14.18; Anal. Calcd for $C_{31}H_{42}O_4Si$: C, 73.47; H, 8.35. Found: C, 73.42; H, 8.39.

(*S*)-6-((*S*)-2-hydroxy-6-methyl-5-oxohept-6-en-2-yl)-3-methylcyclohex-2-enone (13). Yield 34%; R_f=0.14 (7:3, *n*-hexane/EtOAc); **12** decomposes into the GC-MS[4]; ^{1}H NMR (400 MHz, $CDCl_3$) δ 5.97 (t, *J* = 1.0 Hz, 1H), 5.81 (t, *J* = 1.8 Hz, 1H), 5.69 (t, *J* = 1.1 Hz, 1H), 5.25 (d, *J* = 1.3 Hz, 1H), 2.90 – 2.68 (m, 2H), 2.39 – 2.20 (m, 3H), 2.03 (dtd, *J* = 13.1, 4.6, 2.7 Hz, 1H), 1.90 (t, *J* = 1.2 Hz, 3H), 1.81 (t, *J* = 1.2 Hz, 3H), 1.77 – 1.70 (m, 2H), 1.70 – 1.56 (m, 1H), 1.11 (s, 3H); ^{13}C NMR (100 MHz, $CDCl_3$) δ 203.64, 202.32, 163.66, 144.41, 127.46, 124.46, 73.55, 52.80, 34.46, 31.43, 31.35, 25.05, 24.00, 23.22, 17.64; Anal. Calcd for $C_{15}H_{22}O_3$: C, 71.97; H, 8.86. Found: C, 72.03; H, 8.97.

Method B (15a + 14)

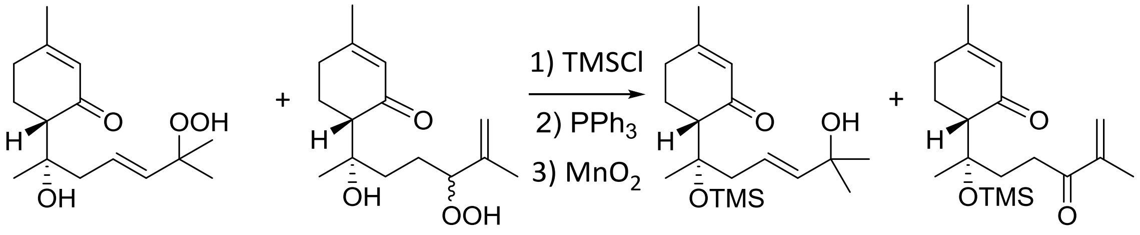

2a **2b-c** **15a** **14**

To an ice cooled solution of **2** (0.65 g, 2.43 mmol) in CH_2Cl_2/Pyridine (2:1, 12 mL) was added TMSCl (1.85 mL, 14.6 mmol, 6 equiv) and DMAP (20 mg). The reaction was stirred for 4 h at 0 °C, and then left at rt for 20 h (the reaction has to be monitored by ^{1}H NMR and not by TLC, since the acidity of SiO_2 cleaves the peroxy silyl group). The reaction mixture was quenched with distilled water at 0 °C, diluted with *n*-hexane/Et_2O (10:1, 220 mL) and washed with HCl (0.5 M, 2 × 10 mL) until acid pH of the extract, then with brine (sat., 10 mL). the organic phase was dried over Na_2SO_4 and the solvent was removed under reduced pressure. Then to a water cooled solution of the crude material in CH_2Cl_2 (2 mL) was added slowly PPh_3 (0.65 g, 2.48 mmol, 1.02 equiv) and the reaction was stirred for 15 min at rt. Then, to the reaction mixture diluted with CH_2Cl_2 (20 mL) was added MnO_2 (6.5 g, 10 equiv in wt.) and the reaction was stirred at rt 1 h. The reaction was filtered over celite and the solvent was removed under reduced pressure. Column chromatographic separation (eluent *n*-hexane/EtOAc in gradient: from *n*-hexane to 6:4) gave in order elution **14** as a transparent liquid (295 mg), and **15a** as a transparent liquid (305 mg).

(*S*)-3-methyl-6-((*S*)-6-methyl-5-oxo-2-(trimethylsilyloxy)hept-6-en-2-yl)cyclohex-2-enone (14). Yield 71%; R_f=0.45 (7:3, *n*-hexane/EtOAc); t_r= 24.81 min >99% purity by GC; $[\alpha]_D$ = +13.6° (*c* 1.1, $CHCl_3$); ^{1}H NMR (400 MHz, $CDCl_3$) δ 6.02 (t, J = 0.9 Hz, 1H), 5.79 (dt, J = 1.8, 1.2 Hz, 1H), 5.76 (qd, J = 1.5, 0.8 Hz, 1H), 2.82 (ddd, J = 15.7, 10.6, 5.9 Hz, 1H), 2.65 (ddd, J = 15.7, 10.4, 5.1 Hz, 1H), 2.47 – 2.36 (m, 1H), 2.31 (dd, J = 10.1, 4.5 Hz, 1H), 2.27 – 2.14 (m, 2H), 2.03 – 1.93 (m, 1H), 1.93 – 1.89 (m, 3H), 1.86 (dd, J = 1.5, 0.9 Hz, 3H), 1.85 – 1.74 (m, 2H), 1.48 (s, 3H), 0.09 (s, 9H); ^{13}C NMR (100 MHz, $CDCl_3$) δ 202.24, 199.61, 160.82, 144.55, 128.06, 124.35, 77.36, 55.33, 33.84, 33.11, 31.24, 27.34, 24.14, 23.84, 17.75, 2.60; GC/MS: *m/z* (%) 309 (M^+, 44), 225 (21), 213 (31), 75 (100); Calcd for $C_{18}H_{30}O_3Si$: C, 67.03; H, 9.38. Found: C, 67.24; H, 9.46.

(*S*)-6-((*S,E*)-6-hydroxy-6-methyl-2-(trimethylsilyloxy)hept-4-en-2-yl)-3-methylcyclohex-2-enone (15a). Yield 82%. R_f=0.18 (7:3, *n*-hexane/EtOAc); t_r= 24.27 min 97% purity by GC; $[\alpha]_D$ = -11.3° (*c* 1.0, $CHCl_3$); ^1H NMR (400 MHz, $CDCl_3$) δ 5.79 (q, J = 1.4 Hz, 1H), 5.69 – 5.65 (m, 2H), 2.51 – 2.38 (m, 2H), 2.27 (dd, J = 8.8, 4.7 Hz, 1H), 2.18 (td, J = 4.1, 2.9 Hz, 2H), 2.15 (dd, J = 5.8, 3.3 Hz, 2H), 2.02 – 1.93 (m, 1H), 1.90 (p, J = 0.9 Hz, 3H), 1.41 (s, 3H), 1.31 – 1.29 (m, 6H), 0.09 – 0.07 (m, 9H); ^{13}C NMR (100 MHz, $CDCl_3$) δ 199.99, 160.65, 141.32, 128.17, 123.39, 77.84, 70.67, 53.97, 42.82, 30.93, 29.91, 29.80, 27.62, 24.23, 23.84, 2.61; GC/MS: *m/z* (%) 309 (M^+, 6), 225 (37), 213 (5), 75 (100); Anal. Anal. Calcd for $C_{18}H_{32}O_3Si$: C, 66.62; H, 9.94. Found: C, 66.57; H, 10.03.

Peroxylippidulcine A (2a)

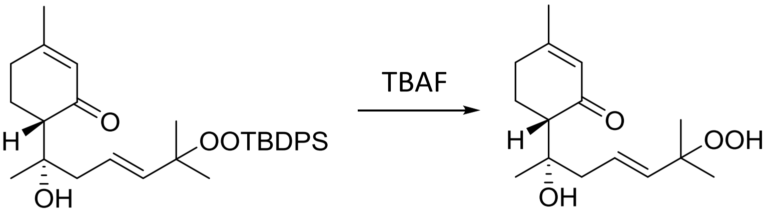

12 **2a**

To a solution of **12** (0.51 g, 1.01 mmol) in THF (5 mL) was added TBAF (1 M, 2 mL, 2.02 mmol, 2 equiv) and reaction was kept stirred at rt for 12 h. The reaction mixture diluted with Et_2O (50 mL), washed with brine (sat., 3 × 5 mL), dried over Na_2SO_4 and the solvent was removed under reduced pressure. Column chromatographic separation (eluent *n*-hexane/EtOAc in gradient: from *n*-hexane to 6:4) gave peroxy lippidulcine A **2a** as a yellowish solid 224 mg; 83% yield; R_f=0.06 (7:3 n-hexane/EtOAc); $[\alpha]_D$ = +43.5° (*c* 1.6, $CHCl_3$) *vs.* lit.[6] $[\alpha]_D$ = +42.0° (*c* 3.2, $CHCl_3$); **2a** decomposes into the GC-MS[4]; ^1H NMR (400 MHz, $CDCl_3$) δ 8.38 (s, 1H), 5.89 – 5.70 (m, 2H), 5.60 (dt, *J* = 16.0, 1.3 Hz, 1H), 5.35 (t, *J* = 1.3 Hz, 1H), 2.38 (dd, *J* = 13.9, 4.5 Hz, 1H), 2.34 – 2.25 (m, 2H), 2.25 – 2.11 (m, 2H), 2.01 (dtd, *J* = 12.9, 4.6, 2.7 Hz, 1H), 1.93 (t, *J* = 1.1 Hz, 3H), 1.69 – 1.57 (m, 1H), 1.31 (d, *J* = 7.0 Hz, 6H), 1.16 (s, 3H). ^{13}C NMR (100 MHz, $CDCl_3$) δ 204.18, 163.84, 137.06, 127.52, 126.61, 81.99, 74.44, 52.09, 43.36, 31.39, 24.91, 24.59, 24.30, 24.10, 23.83; Anal. Calcd for $C_{15}H_{24}O_4$: C, 67.14; H, 9.01. Found: C, 67.17; H, 9.03.

Lippidulcine A (3a)

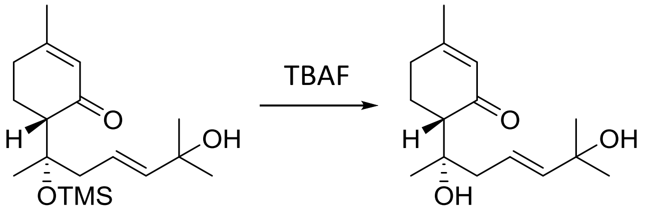

15a **3a**

To a stirred solution of **15a** (0.18 g, 0.56 mmol) in MeCN (0.5 mL) was added H_2O (60 mg, 3.36 mmol, 6 equiv) and TBAF (1 M, 1.1 mL, 1.12 mmol, 2 equiv) and the reaction was stirred for 30 min at rt. The reaction was diluted with CH_2Cl_2 (30 mL) and washed with brine (sat., 3 × 5 mL), dried over Na_2SO_4 and the solvent was removed under reduced pressure. Column chromatographic separation (eluent *n*-hexane/EtOAc in gradient: from *n*-hexane to 4:6) gave **3a** as a yellowish solid (110 mg); 78% yield; R_f=0.37 (1:9, *n*-hexane/EtOAc); $[\alpha]_D$= +132° (*c* 1.3, $CHCl_3$) *vs.* lit.[6] $[\alpha]_D$ = +123.6° (*c* 0.1, $CHCl_3$); ^1H NMR (400 MHz, $CDCl_3$) δ 5.84 (dq, J = 2.1, 1.1 Hz, 1H), 5.79 (ddd, J = 15.8, 8.2, 5.9 Hz, 1H), 5.64 (dt, J = 15.7, 1.2 Hz, 1H), 5.21 (s, 1H), 2.36 (dd, J = 13.8, 4.6 Hz, 1H), 2.33 – 2.21 (m, 2H), 2.19 (dd, J = 5.9, 1.5 Hz, 1H), 2.16 (d, J = 8.1 Hz, 1H), 1.99 (dtd, J = 13.0, 4.5, 2.8 Hz, 1H), 1.95 – 1.89 (m, 3H), 1.63 (tdd, J = 13.3, 11.2, 5.8 Hz, 1H), 1.29 (s, 6H), 1.15 (s, 3H); ^{13}C NMR (100 MHz, $CDCl_3$) δ 204.15, 163.51, 141.33, 127.60, 122.42, 74.37, 70.66, 52.01, 43.16, 31.39, 29.96, 29.89, 24.89, 24.09, 23.88; GC/MS: decomposes[4] into two compounds t_r(3-methyl-2-cyclohexen-1-one) = 7.78 min; *m/z* (%) 110 (M^+, 45), 54 (21), 39 (19), 82 (100), t_r(2° fragment)= 8.93 min; *m/z* (%) 124 (M^+, 20), 109 (48), 43 (100); Anal. Calcd for $C_{15}H_{24}O_3$: C, 71.39; H, 9.59. Found: C, 71.35; H, 9.68.

Epilippidulcine A (3d).

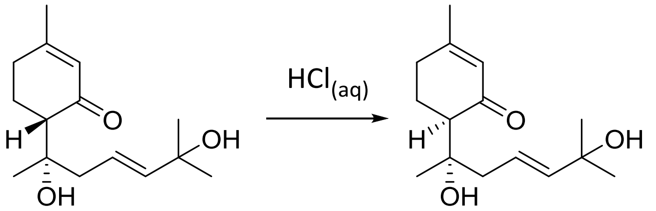

3a **3d**

To a stirred solution of lippidulcine A **3a** (100 mg, 0.40 mmol) in MeCN (0.5 mL) was added HCl (1 M, 0.8 mL, 0.80 mmol, 2 equiv) and the reaction was stirred overnight at rt. The reaction was diluted with CH_2Cl_2 (30 mL) and washed with brine (sat., 3 × 5 mL), dried over Na_2SO_4 and the solvent was removed under reduced pressure.

Column chromatographic separation (eluent *n*-hexane/EtOAc in gradient: from *n*-hexane to 4:6) gave **3d** as a colourless liquid (24 mg); 24% yield; R_f=0.30 (1:9 *n*-hexane/EtOAc); $[\alpha]_D$ = -107° (*c* 1.2, $CHCl_3$) vs. lit.[7] $[\alpha]_D$ = -118.4° (*c* 0.5, $CHCl_3$); ^1H NMR (400 MHz, $CDCl_3$) δ 5.83 (dq, J = 2.5, 1.1 Hz, 1H), 5.71 (ddd, J = 15.7, 7.6, 6.5 Hz, 1H), 5.59 (dt, J = 15.6, 1.1 Hz, 1H), 5.05 (s, 1H), 2.39 – 2.29 (m, 4H), 2.14 (ddd, J = 13.9, 7.6, 1.0 Hz, 1H), 2.06 (dtd, J = 13.0, 4.5, 2.8 Hz, 1H), 1.94 (t, J = 1.2 Hz, 3H), 1.87 (br s, 1H), 1.77 (dddd, J = 13.9, 13.0, 11.2, 5.5 Hz, 1H), 1.27 (d, J = 1.4 Hz, 6H), 1.16 (s, 3H); ^{13}C NMR (100 MHz, $CDCl_3$) δ 203.34, 163.46, 141.15, 127.59, 122.98, 74.69, 70.68, 54.64, 40.91, 31.68, 29.80, 29.76, 26.16, 24.97, 24.11; GC/MS: decomposes[4] into two compounds t_r (3-methyl-2-cyclohexen-1-one) = 7.78 min; *m/z* (%) 110 (M^+, 45), 54 (21), 39 (19), 82 (100), t_r (2° fragment)= 8.93 min; *m/z* (%) 124 (M^+, 20), 109 (48), 43 (100); Anal. Calcd for $C_{15}H_{24}O_3$: C, 71.39; H, 9.59. Found: C, 71.42; H, 9.54.

(*S*)-6-((*2S,5R*)-5-Hydroxy-6-methyl-2-(trimethylsilyloxy)hept-6-en-2-yl)-3-methylcyclohex-2-enone (15b)

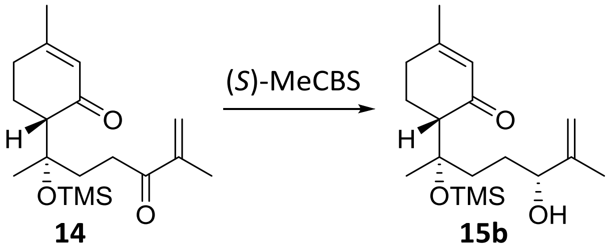

To a stirred solution of (*S*)-MeCBS (1 M, 0.85 mL, 0.85 mmol, 1.2 equiv) in CH_2Cl_2 (3 mL) was added $BH_3{\cdot}Me_2S$ (10.5 M, 74 μL, 0.78 mmol, 1.1 equiv) and the reaction was stirred at rt for 1 h. Then at −78 °C, a solution of **14** (0.23 g, 0.71 mmol) in CH_2Cl_2 (6 mL) was added over a 45 min period. The reaction was kept stirring for 17 h at −78 °C, then for 2 days at −60 °C. The reaction was quenched with slow addition of MeOH (1 mL) at −70 °C, then let stirred for 30 min at rt. Finally the reaction was diluted with CH_2Cl_2 (30 mL) and extracted with brine (sat., 3 × 5 mL), dried over Na_2SO_4 and the solvent was removed under reduced pressure. Column flash-chromatographic separation (eluent *n*-hexane/EtOAc, 8:2) gave **15b** as a colorless-pale green liquid (184 mg); 80% yield; R_f=0.21 (7:3 *n*-hexane/EtOAc); t_r= 25.36 min >99% purity by GC; $[\alpha]_D$= –1.6° (*c* 1.2, $CHCl_3$); ^1H NMR (400 MHz, $CDCl_3$) δ 5.80 (h, J = 1.4 Hz, 1H), 4.93 (dq, J = 1.9, 0.9 Hz, 1H), 4.82 (ddt, J = 2.0, 1.5, 0.7 Hz, 1H), 4.03 (t, J = 5.7 Hz, 1H), 2.49 – 2.39 (m, 1H), 2.33 (dd, J = 8.8, 4.8 Hz, 1H), 2.21 (d, J

= 4.1 Hz, 1H), 2.20 – 2.12 (m, 2H), 2.05 – 1.94 (m, 1H), 1.90 (dd, J = 1.4, 0.8 Hz, 3H), 1.80 – 1.73 (m, 1H), 1.72 (ddt, J = 1.3, 0.9, 0.4 Hz, 3H), 1.65 – 1.58 (m, 2H), 1.44 (dd, J = 15.2, 5.6 Hz, 1H), 1.44 (s, 3H), 0.09 (s, 9H); ^{13}C NMR (100 MHz, $CDCl_3$) δ 200.17, 161.08, 147.54, 127.96, 110.77, 77.87, 75.78, 53.97, 35.20, 30.81, 30.04, 27.22, 24.02, 23.78, 17.53, 2.50; GC/MS: *m/z* (%) 309 (M^+, 9), 225 (13), 105 (98), 75 (100); Anal. Calcd for $C_{18}H_{32}O_3Si$: C, 66.62; H, 9.94. Found: C, 66.75; H, 9.81.

(*S*)-6-((*2S,5S*)-5-Hydroxy-6-methyl-2-(trimethylsilyloxy)hept-6-en-2-yl)-3-methylcyclohex-2-enone (15c)

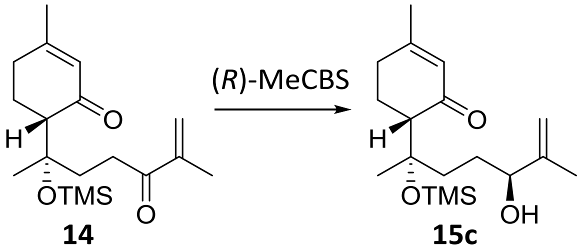

The same procedure and work-up adopted for synthesis of **15b** but with (*R*)-MeCBS. In this case the reaction was faster. Colourless liquid (207 mg); 82% yield; R_f=0.25 (7:3 *n*-hexane/EtOAc); t_r= 25.31 min >99% purity by GC; $[\alpha]_D$= +3.5° (*c* 1.1, $CHCl_3$); ^{1}H NMR (400 MHz, $CDCl_3$) δ 5.79 (q, J = 1.5 Hz, 1H), 4.94 (dt, J = 1.9, 1.0 Hz, 1H), 4.81 (ddq, J = 2.9, 1.4, 0.7 Hz, 1H), 4.01 (t, J = 5.9 Hz, 1H), 2.49 – 2.38 (m, 1H), 2.31 (dd, J = 9.0, 4.8 Hz, 1H), 2.23 – 2.11 (m, 3H), 2.05 – 1.93 (m, 1H), 1.90 (dt, J = 1.3, 0.7 Hz, 3H), 1.73 (ddd, J = 1.4, 0.9, 0.4 Hz, 3H), 1.71 – 1.63 (m, 2H), 1.59 – 1.47 (m, 2H), 1.44 (s, 3H), 0.08 (s, 9H); ^{13}C NMR (100 MHz, $CDCl_3$) δ 200.24, 161.13, 147.70, 128.15, 110.84, 78.02, 76.18, 54.26, 35.17, 31.01, 29.97, 27.51, 24.23, 23.94, 17.85, 2.64; GC/MS: *m/z* (%) 309 (M^+, 8), 225 (20), 213 (5), 75 (100); Anal. Calcd for $C_{18}H_{32}O_3Si$: C, 66.62; H, 9.94. Found: C, 66.54; H, 10.07.

Lippidulcine B (3b)

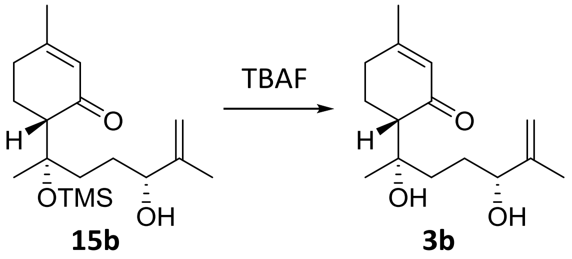

To a stirred solution of **15b** (52.5 mg, 0.162 mmol) in MeCN (0.5 mL) was added H_2O (18 mg, 0.97 mmol, 6 equiv) and TBAF (1 M, 320 μL, 0.32 mmol, 2 equiv) and the reaction was stirred for 30 min at rt. The reaction was diluted with CH_2Cl_2 (30 mL) and washed with brine (3 × 5 mL), dried over Na_2SO_4 and the solvent was removed under reduced pressure. Column flash chromatographic separation (eluent *n*-hexane/EtOAc 1:9) gave **3b** as a colourless liquid 30 mg; 73% yield; R_f=0.37 (1:9 *n*-hexane/EtOAc); $[\alpha]_D$ = +123° (*c* 1.5, $CHCl_3$) vs. lit.[5] $[\alpha]_D$ = +113.3° (*c* 0.4, $CHCl_3$); **3b** decomposes into the GC-MS[4]; ^{1}H NMR (400 MHz, $CDCl_3$) δ 5.94 – 5.79 (m, 1H), 5.54 (s, 1H), 4.96 (dt, J = 2.0, 1.0 Hz, 1H), 4.81 (dtt, J = 2.2, 1.5, 1.0 Hz, 1H), 4.04 (t, J = 6.0 Hz, 1H), 3.20 (s, 1H), 2.45 (dd, J = 14.0, 4.6 Hz, 1H), 2.41 – 2.33 (m, 1H), 2.28 (ddd, J = 18.5, 5.1, 2.6 Hz, 1H), 2.03 – 1.97 (m, 1H), 1.95 (td, J = 1.2, 0.6 Hz, 3H), 1.72 (ddd, J = 1.4, 0.9, 0.4 Hz, 3H), 1.71 – 1.49 (m, 5H), 1.18 (s, 3H); ^{13}C NMR (100 MHz, $CDCl_3$) δ 204.02, 163.71, 147.83, 127.65, 110.52, 75.91, 74.23, 52.27, 36.53, 31.42, 29.20, 25.14, 24.15, 23.74, 18.28; Anal. Calcd for $C_{15}H_{24}O_3$: C, 71.39; H, 9.59. Found: C, 71.29; H, 9.67.

Lippidulcine C (3c)

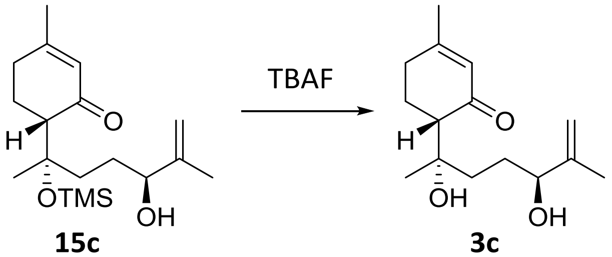

15c **3c**

The same procedure and work-up adopted for synthesis of **3b**. Colourless-yellowish liquid (33 mg); 79% yield; R_f=0.37 (1:9, *n*-hexane/EtOAc); $[\alpha]_D$ = +92.1° (*c* 1.7, $CHCl_3$) *vs.* lit.[5] $[\alpha]_D$ = +119.8° (*c* 0.7, $CHCl_3$); **3c** decomposes into the GC-MS[4]; ^{1}H NMR (400 MHz, $CDCl_3$) δ 5.86 (br s, 1H), 5.47 (s, 1H), 4.96 (ddt, J = 2.0, 1.0, 0.4 Hz, 1H), 4.82 (ddd, J = 2.1, 1.5, 0.6 Hz, 1H), 4.03 (t, J = 5.6 Hz, 1H), 2.88 (s, 1H), 2.41 (dd, J = 14.0, 4.8 Hz, 1H), 2.36 (br d, J = 12.2 Hz, 1H), 2.28 (ddd, J = 18.6, 5.0, 2.3 Hz, 1H), 2.02 (ddt, J = 15.7, 4.6, 2.6 Hz, 1H), 1.95 (dq, J = 1.4, 0.5 Hz, 3H), 1.83 – 1.73 (m, 1H), 1.72 (ddd, J = 1.4, 0.9, 0.5 Hz, 3H), 1.69 – 1.51 (m, 4H), 1.17 (s, 3H); ^{13}C NMR (100 MHz, $CDCl_3$) δ 204.03, 163.73, 147.93, 127.64, 110.57, 75.75, 74.14, 52.25, 36.16, 31.43, 28.82, 25.13, 24.15, 23.81, 18.28; Anal. Calcd for $C_{15}H_{24}O_3$: C, 71.39; H, 9.59. Found: C, 71.43; H, 9.65.

3. Sensorial evaluation criteria

A panel of four persons of different age, tasted a sample of 3 mg of each substance.

4. Notes and references

1. Moreira, J. A.; Correa, A. G. *Tetrahedron: Asymmetry*, **2003**,*14*, 3787–3795.
2. Yadav, J.S.; Vijaya, E. B.; Srihari, P. *Tetrahedron*, **2010**, *66*, 1997–2004.
3. Kim, J. H.; Lim, H. J.; Cheon, S. H. *Tetrahedron*, **2003**, *59*, 7501–7507.
4. The high temperature of GC-injector induces the spontaneous retro aldolic reaction.
5. Souto-Bachiller, F. A.; De Jesus-Echevarria, M.; Cardenas-Gonzalez, O. E.; Acuna-Rodriguez, M. F.; Melendez, P. A.; Romero-Ramsey, L. P*hytochemistry*, **1997**, *44*, 1077–1086.
6. Ono, M.; Tsuru, T.; Abe, H.; Eto, S.;Okawa, M.; Abe, F.; Kinjo, J.; Ikeda, T.; Nohara, T. *J. Nat. Prod.* **2006**, *69*, 1417–1420.
7. Ono, M.; Morinaga, H.; Masuoka, C.; Ikeda, T.; Okawa, M.; Junei Kinjo, J.; Nohara, T. *Chem. Pharm. Bull.*, **2005**, *53*, 1175-1177.

5. Copies of ^{1}H and ^{13}C NMR spectra

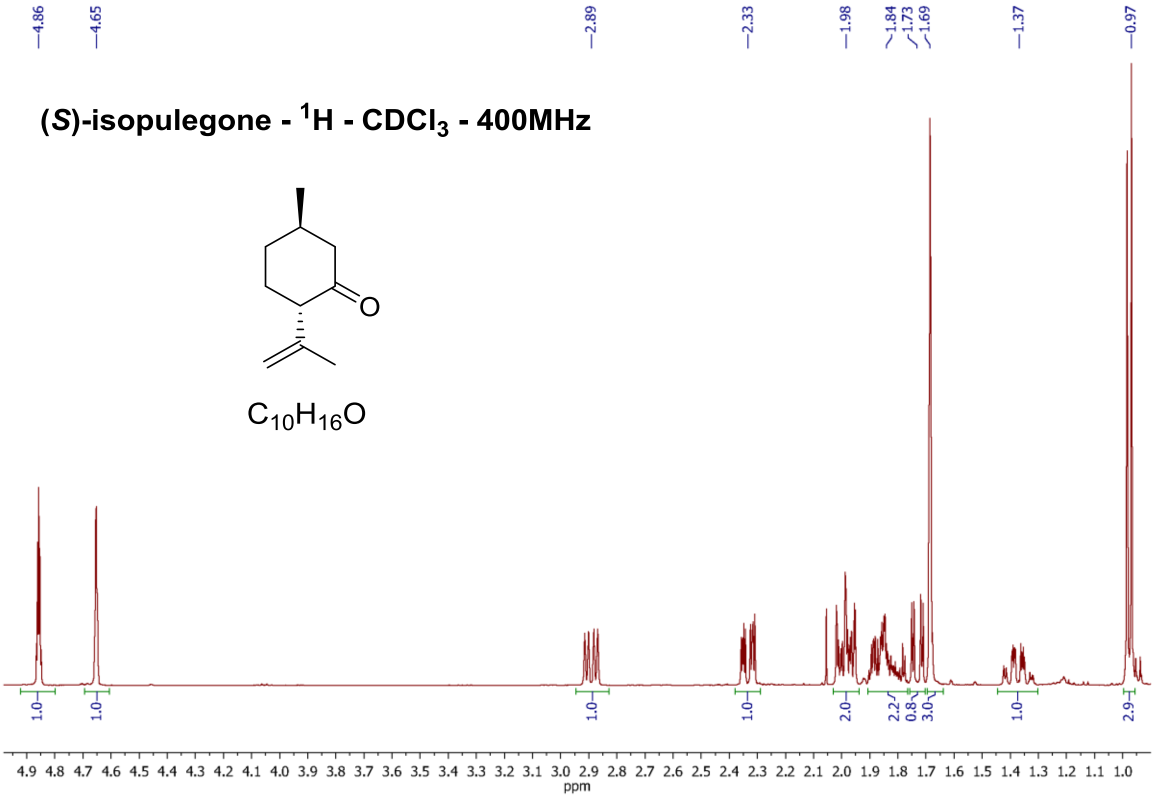

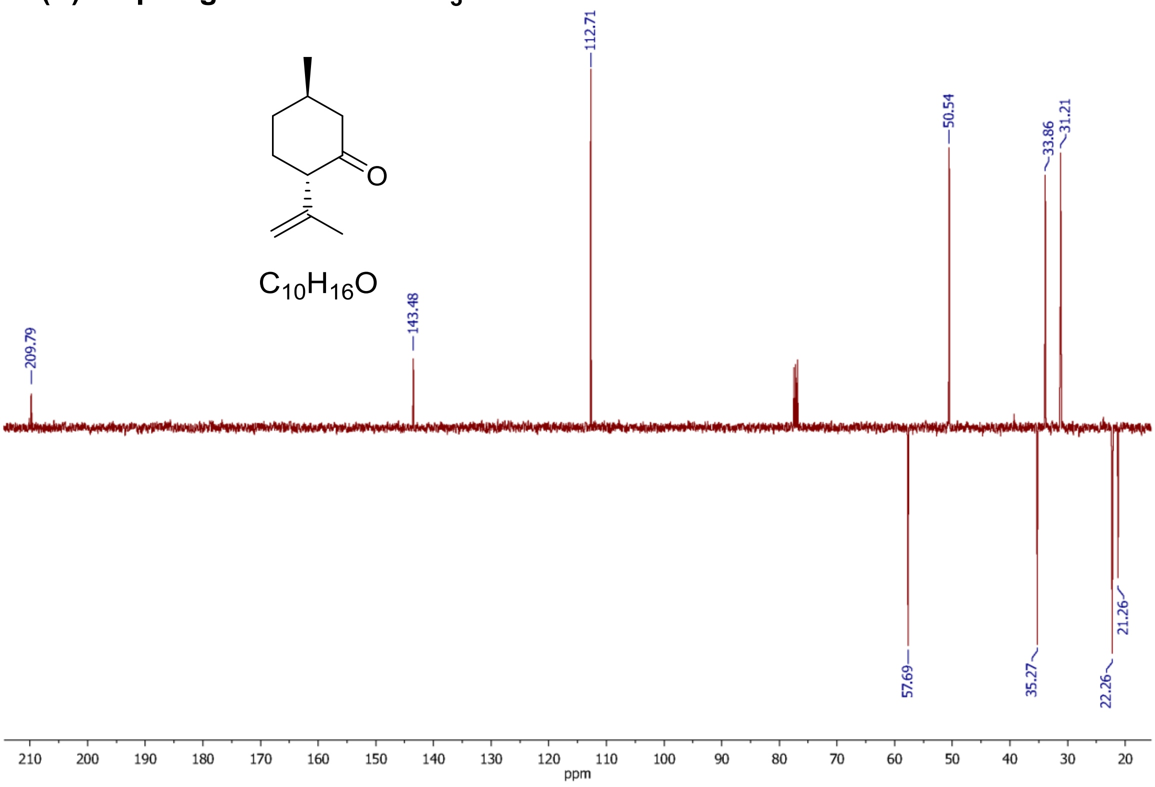

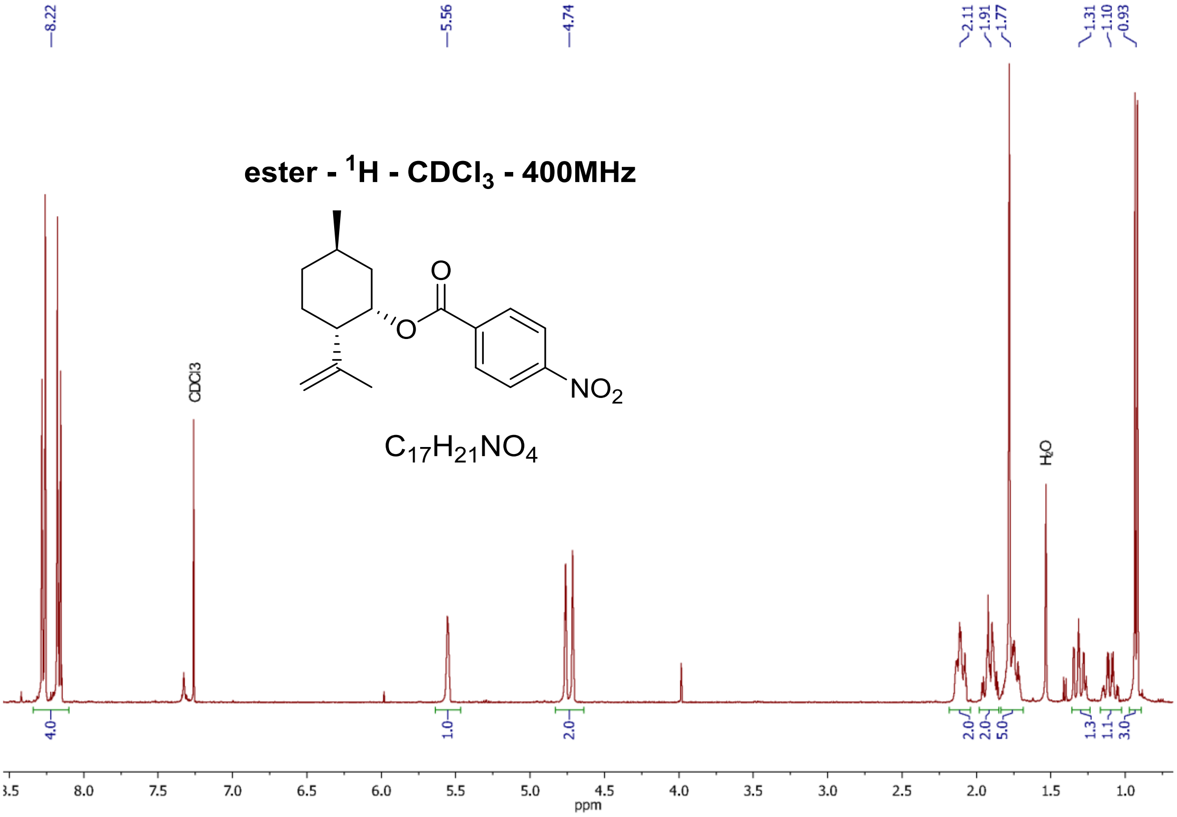
ester - ^{1}H - $CDCl_3$ - 400MHz
O
O
NO_2
$C_{17}H_{21}NO_4$
8.22
5.56
4.74
2.11
1.91
1.77
1.31
1.10
0.93
CDCl3
H2O
4.0
1.0
2.0
2.0
2.0
5.0
1.3
1.1
3.0
ppm

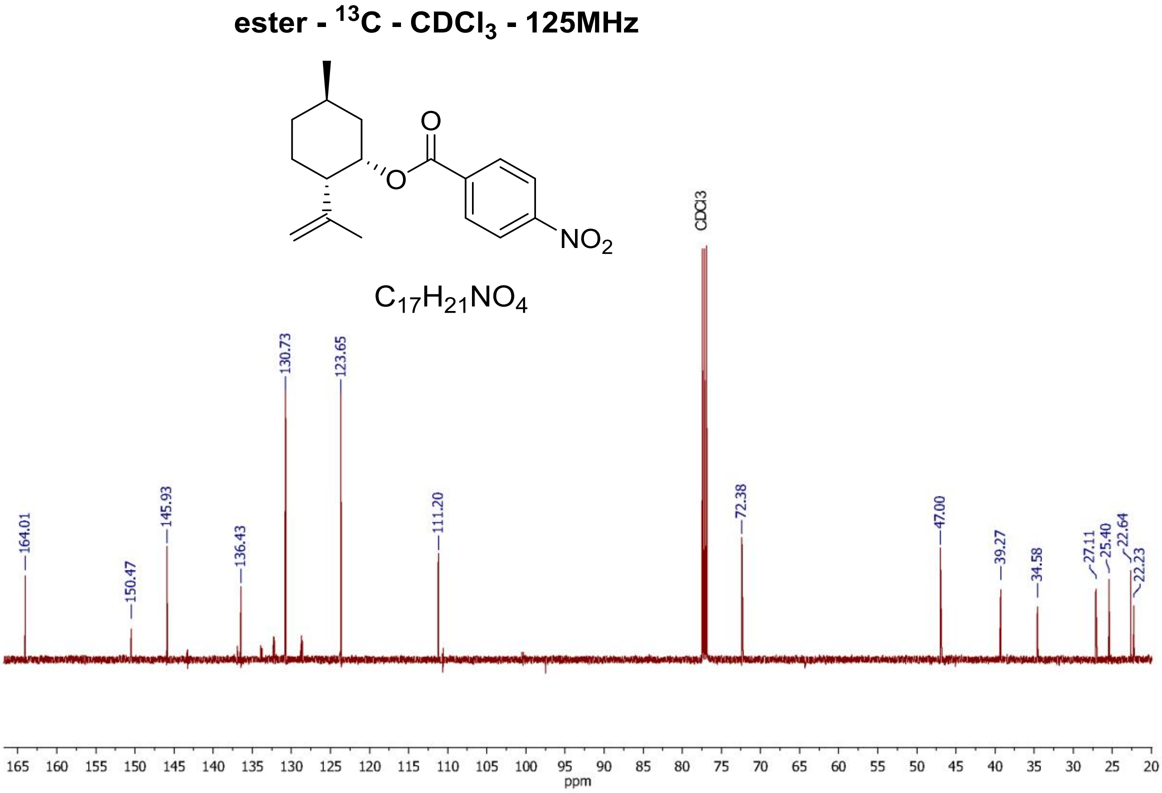
ester - ^{13}C - $CDCl_3$ - 125MHz
O
O
NO_2
$C_{17}H_{21}NO_4$
CDCl3
164.01
150.47
145.93
136.43
130.73
123.65
111.20
72.38
47.00
39.27
34.58
27.11
25.40
22.64
22.23
ppm

4 - (+)-Neoisopulegol - ^{1}H - $CDCl_3$ - 400MHz

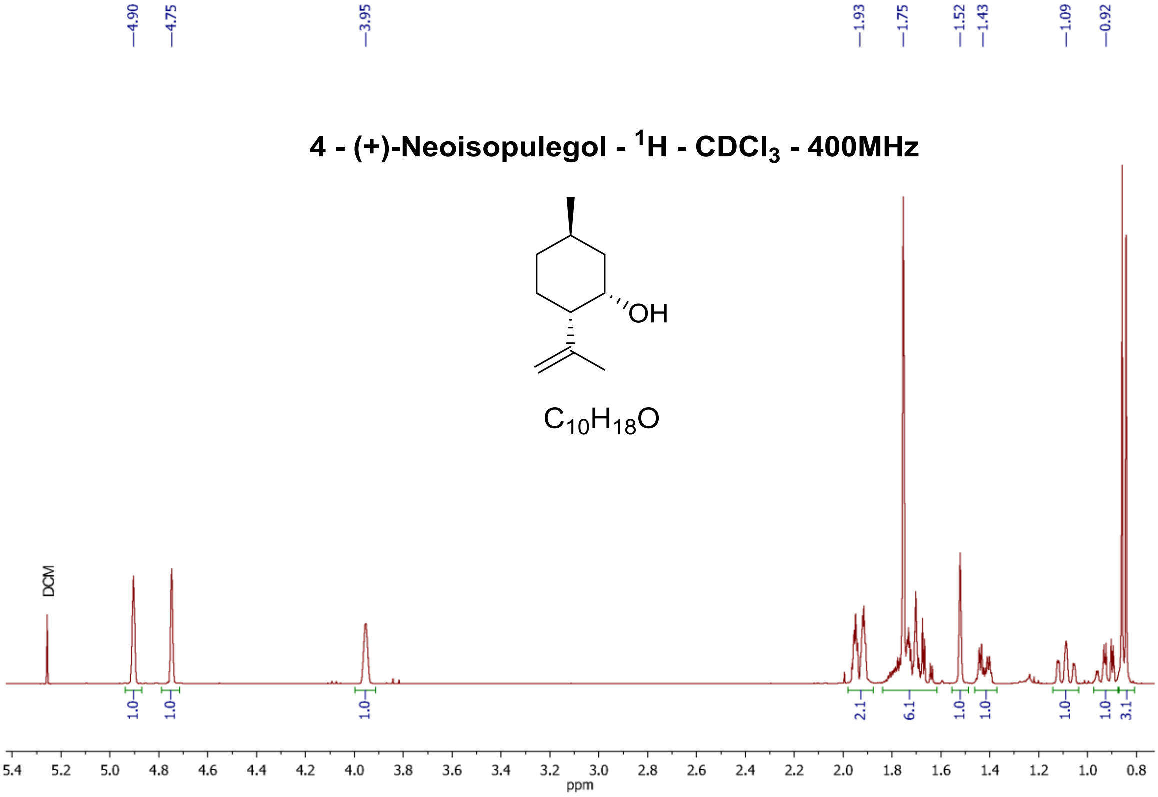

4 - (+)-Neoisopulegol - ^{13}C - $CDCl_3$ - 100MHz

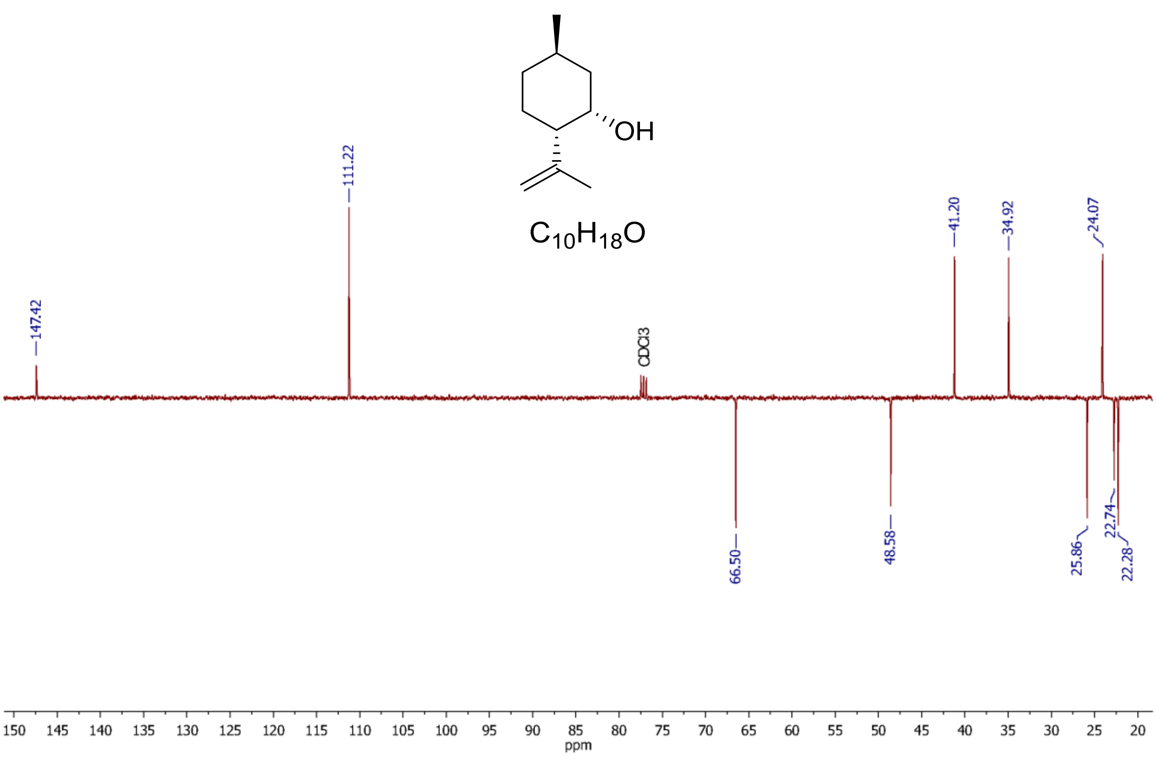

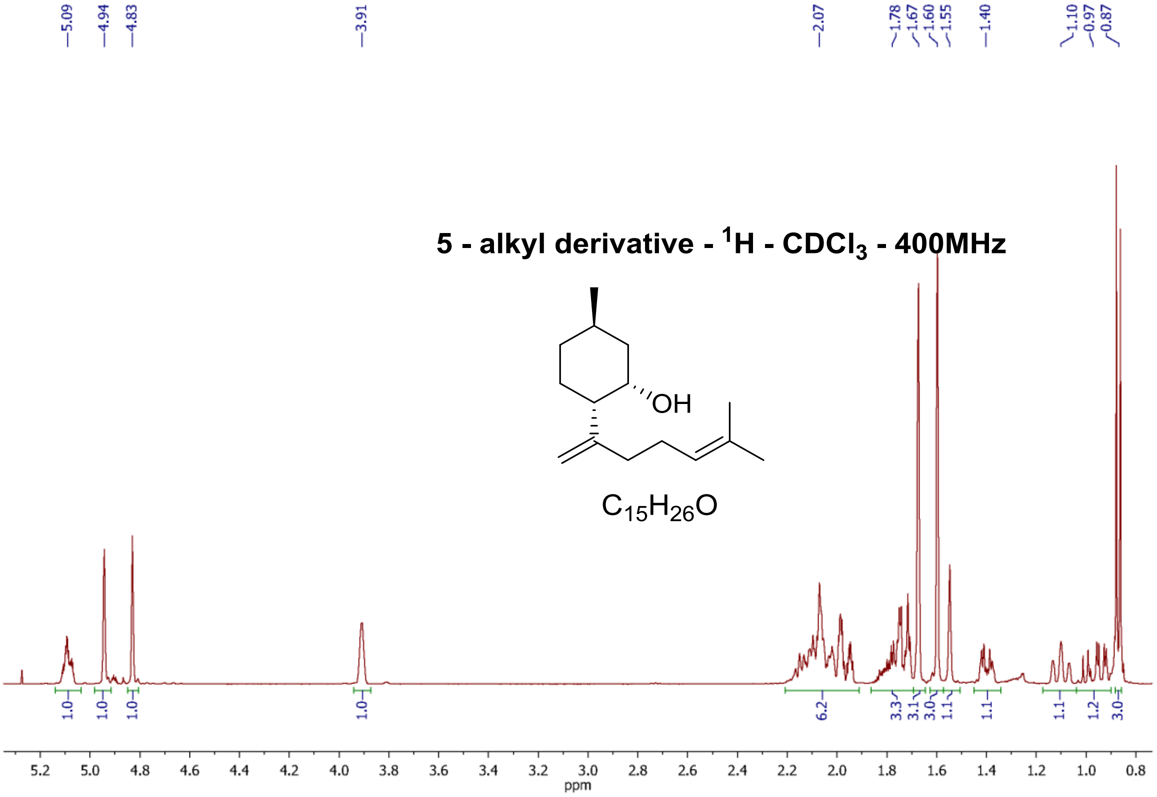
5 - alkyl derivative - ^{1}H - $CDCl_3$ - 400MHz
OH
$C_{15}H_{26}O$
5.09
4.94
4.83
3.91
2.07
1.78
1.67
1.60
1.55
1.40
1.10
0.97
0.87
1.0
1.0
1.0
1.0
6.2
3.3
3.1
3.0
1.1
1.1
1.1
1.2
3.0
ppm

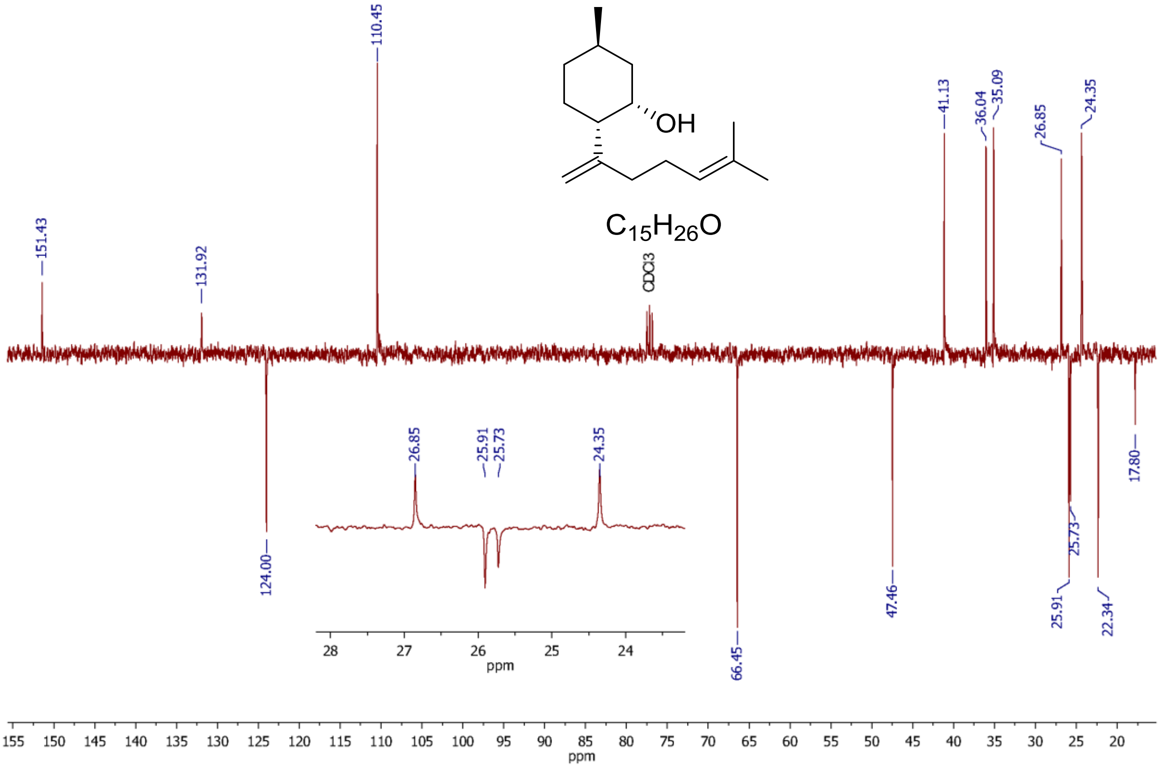
5 - alkyl derivative - ^{13}C - $CDCl_3$ - 100MHz
OH
$C_{15}H_{26}O$
CDCl3
151.43
131.92
110.45
124.00
66.45
47.46
41.13
36.04
35.09
26.85
24.35
25.73
25.91
22.34
17.80
26.85
25.91
25.73
24.35
ppm
ppm

6 - epoxide - ^{1}H - $CDCl_3$ - 400MHz

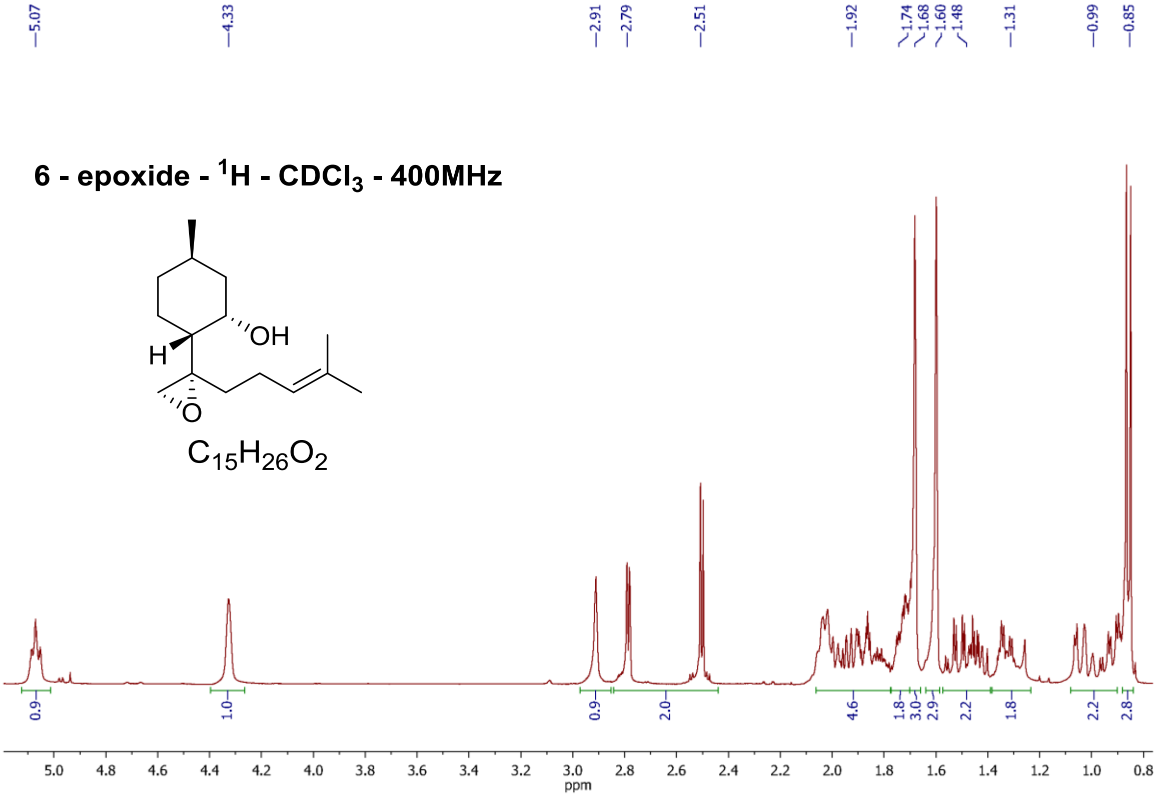

6 - epoxide - ^{13}C - $CDCl_3$ - 125MHz

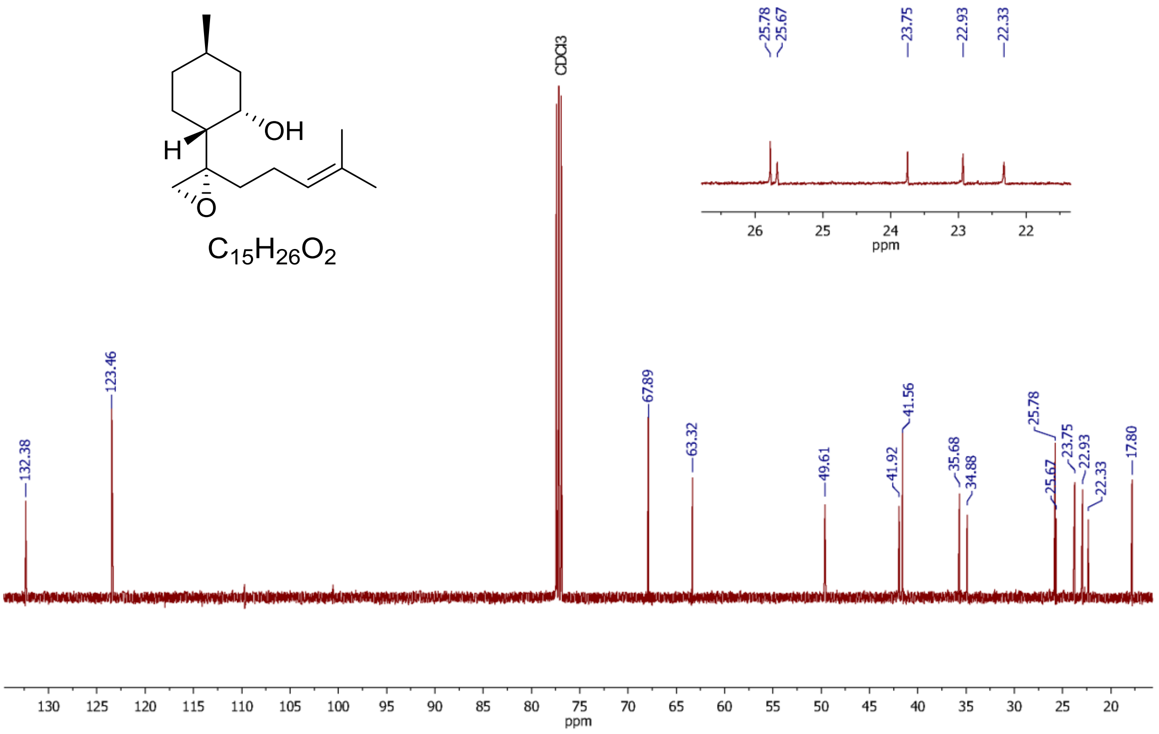

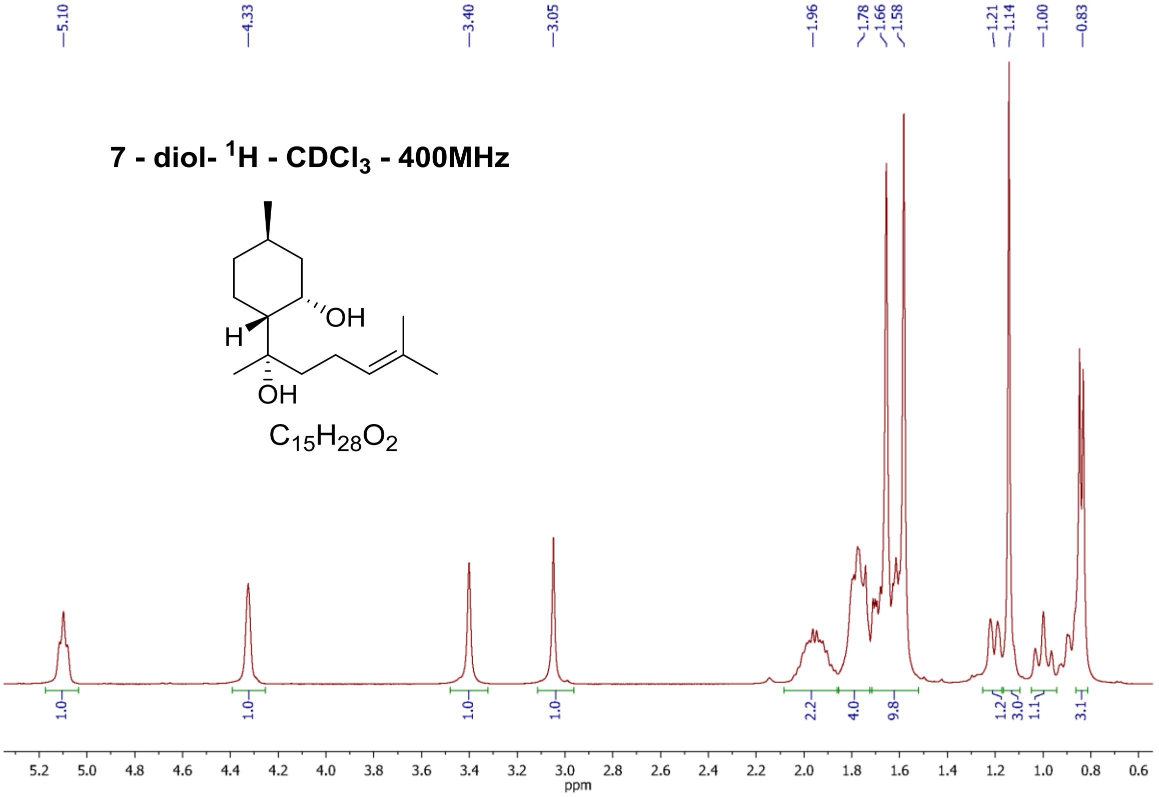
7 - diol- 1H - CDCl3 - 400MHz
OH
H
OH
C15H28O2
5.10
4.33
3.40
3.05
1.96
1.78
1.66
1.58
1.21
1.14
1.00
0.83
1.0
1.0
1.0
1.0
2.2
4.0
9.8
1.2
3.0
1.1
3.1
ppm

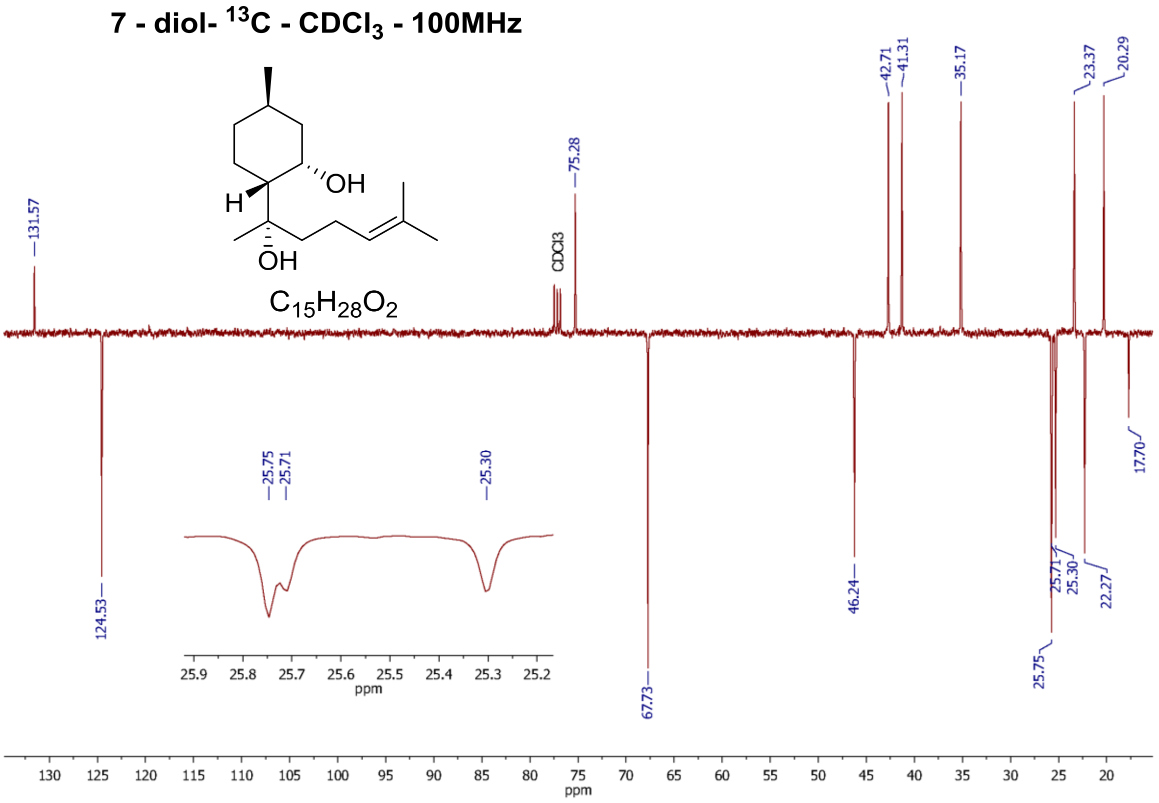
7 - diol- 13C - CDCl3 - 100MHz
OH
H
OH
C15H28O2
131.57
124.53
CDCl3
75.28
67.73
46.24
42.71
41.31
35.17
23.37
20.29
17.70
25.75
25.71
25.30
22.27
ppm

8 - ketone - ^{1}H - $CDCl_3$ - 400MHz

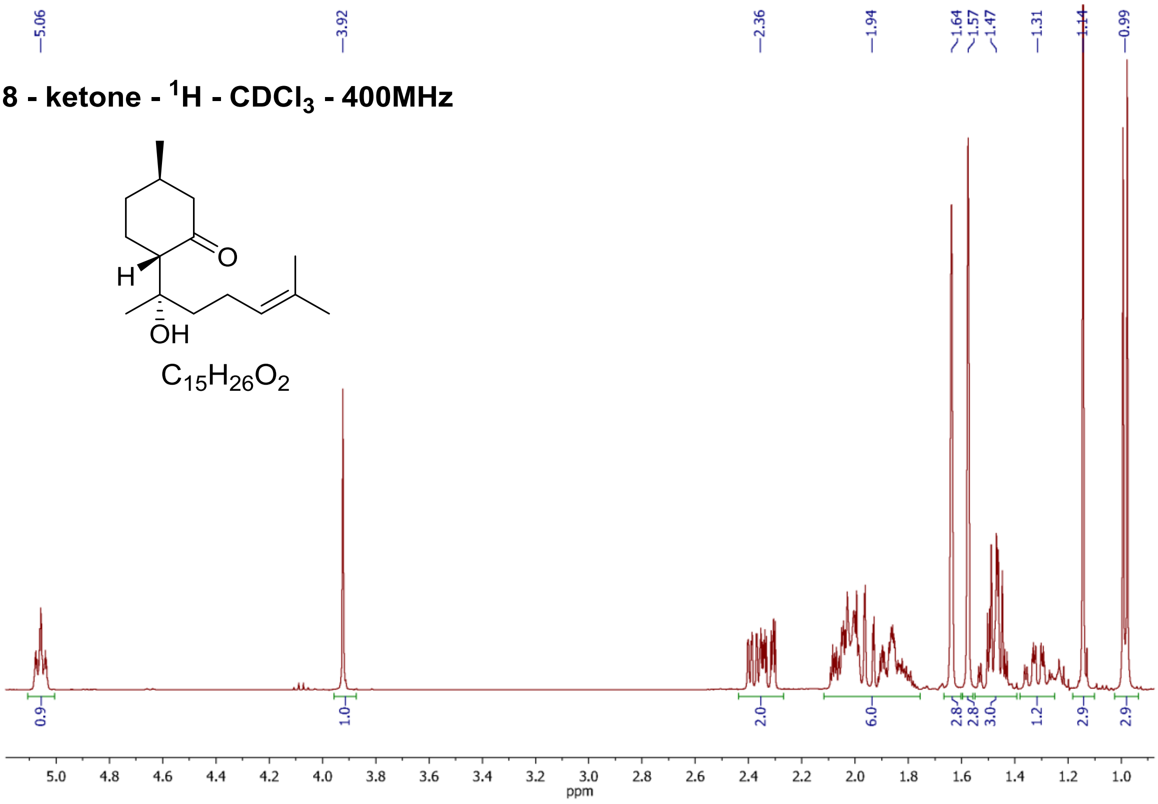

8 - ketone - ^{13}C - $CDCl_3$ - 100MHz

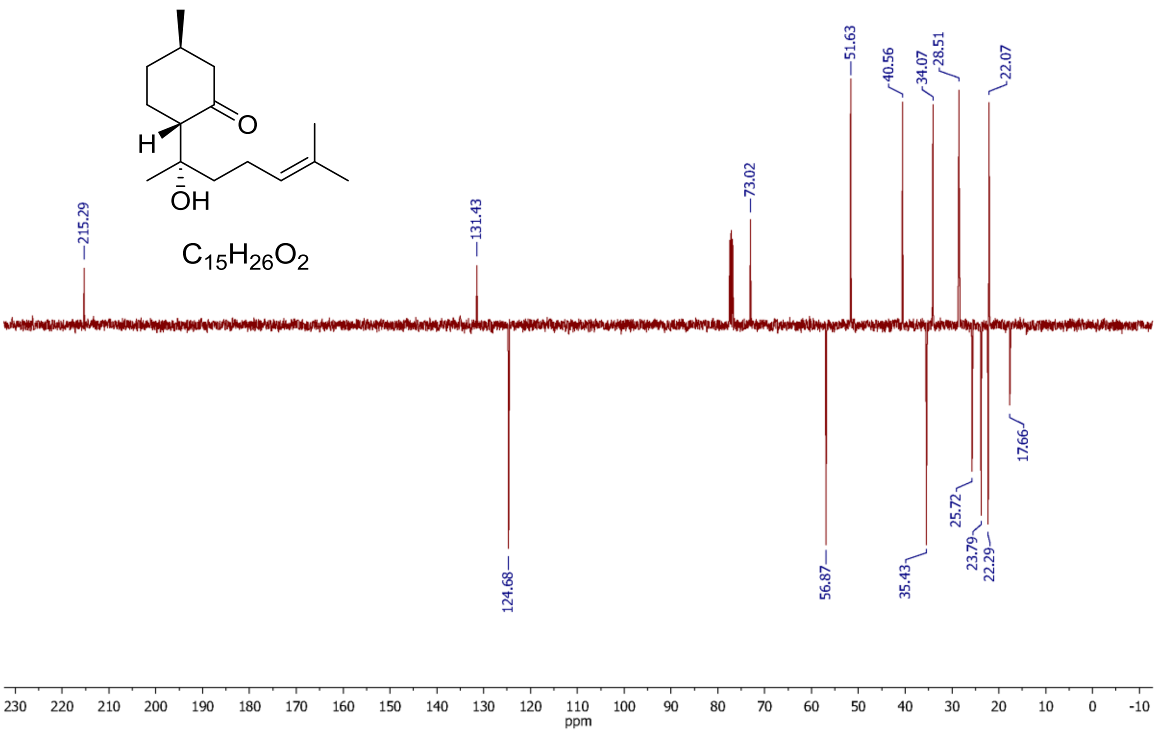

9 - trimethyl silyl ether derivative - ^1H - $CDCl_3$ - 400MHz

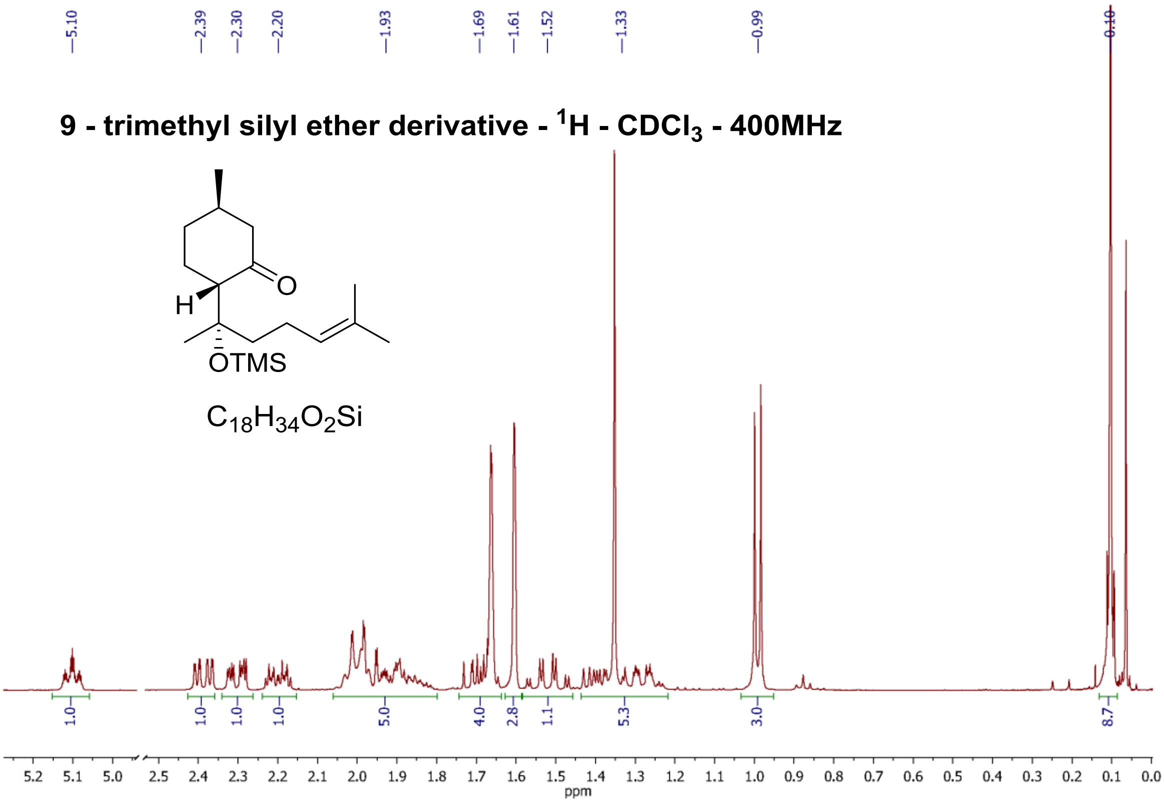

9 - trimethyl silyl ether derivative - ^{13}C - $CDCl_3$ - 100MHz

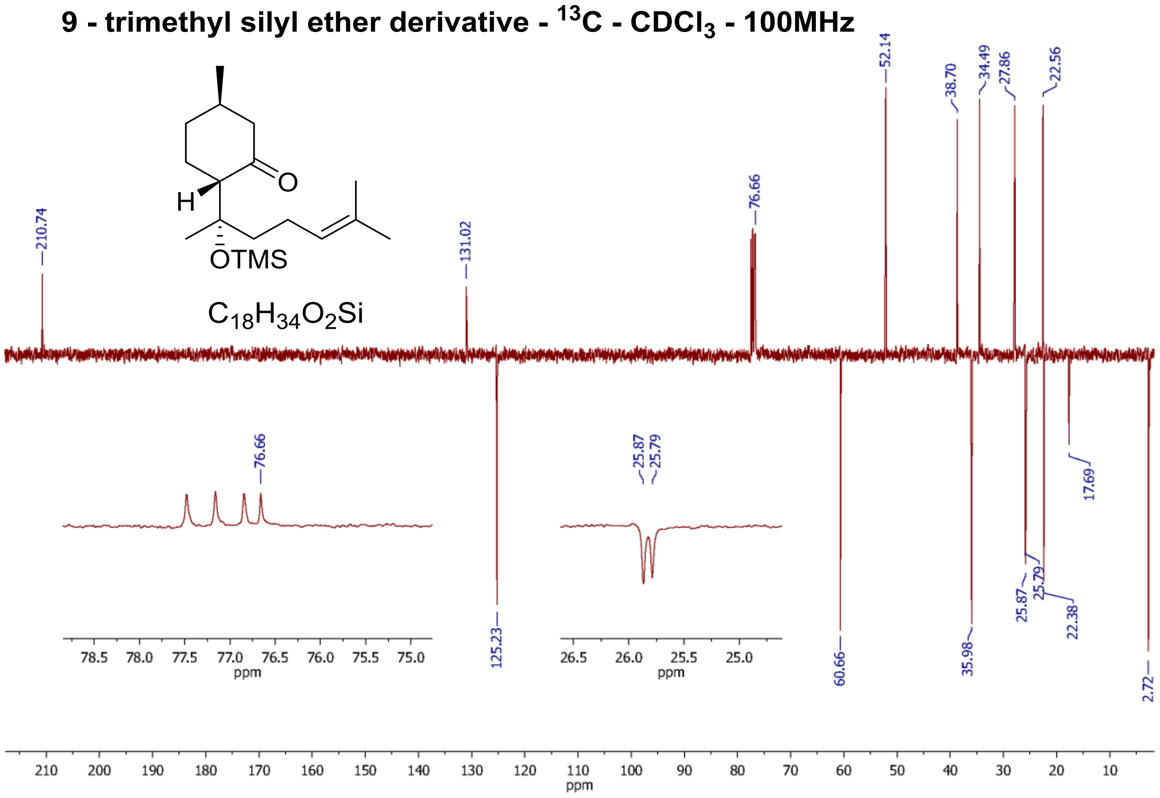

10a - triethyl silyl enol ether - ^{1}H - $CDCl_3$ - 400MHz

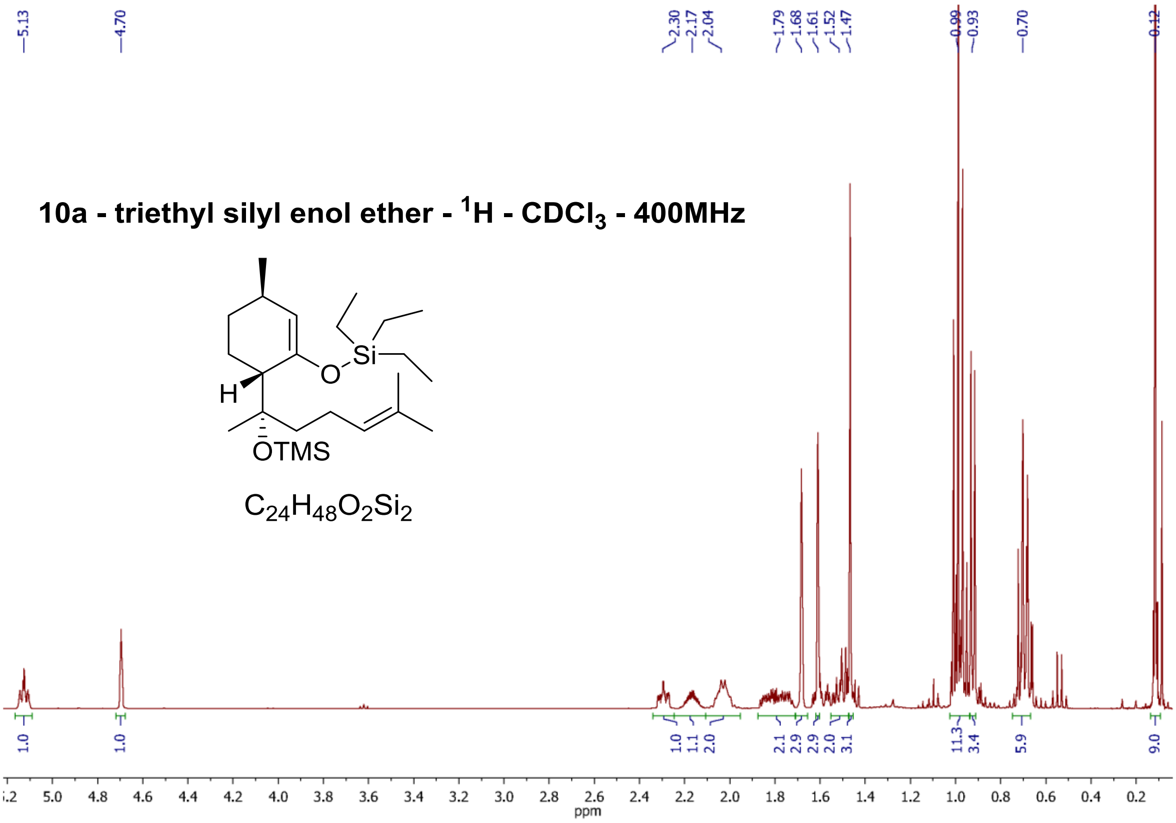

10a - triethyl silyl enol ether - ^{13}C - $CDCl_3$ - 100MHz

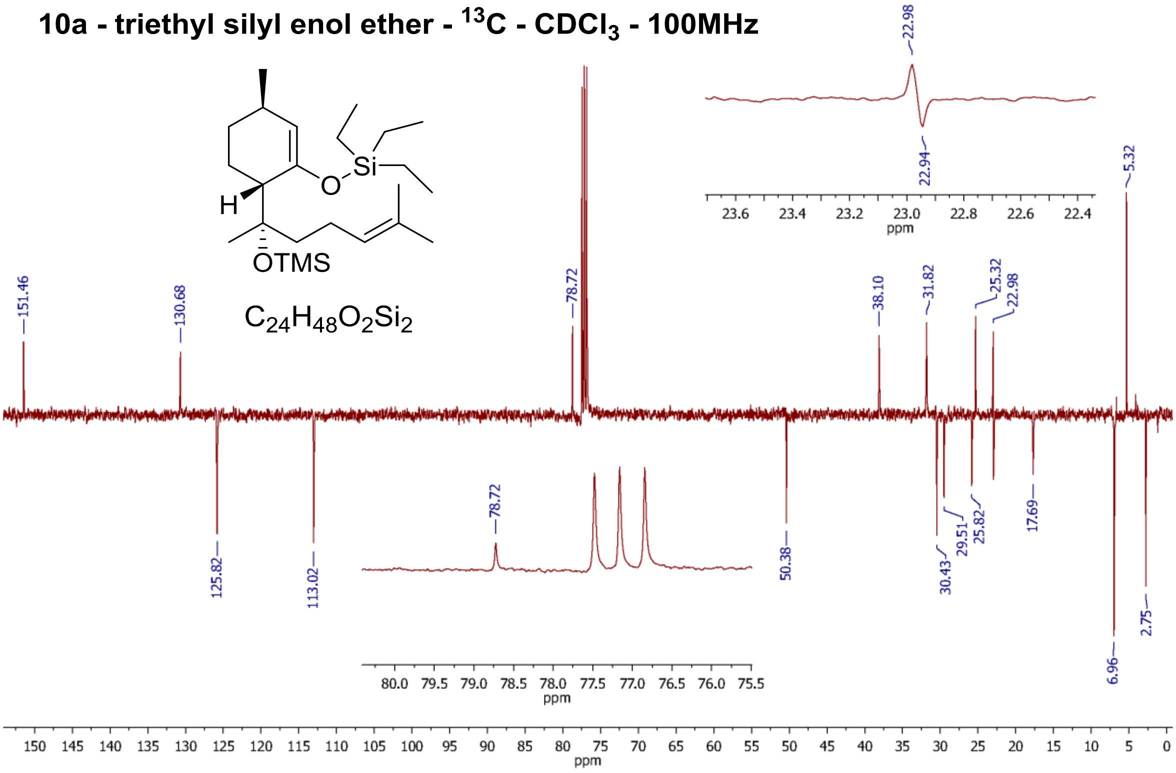

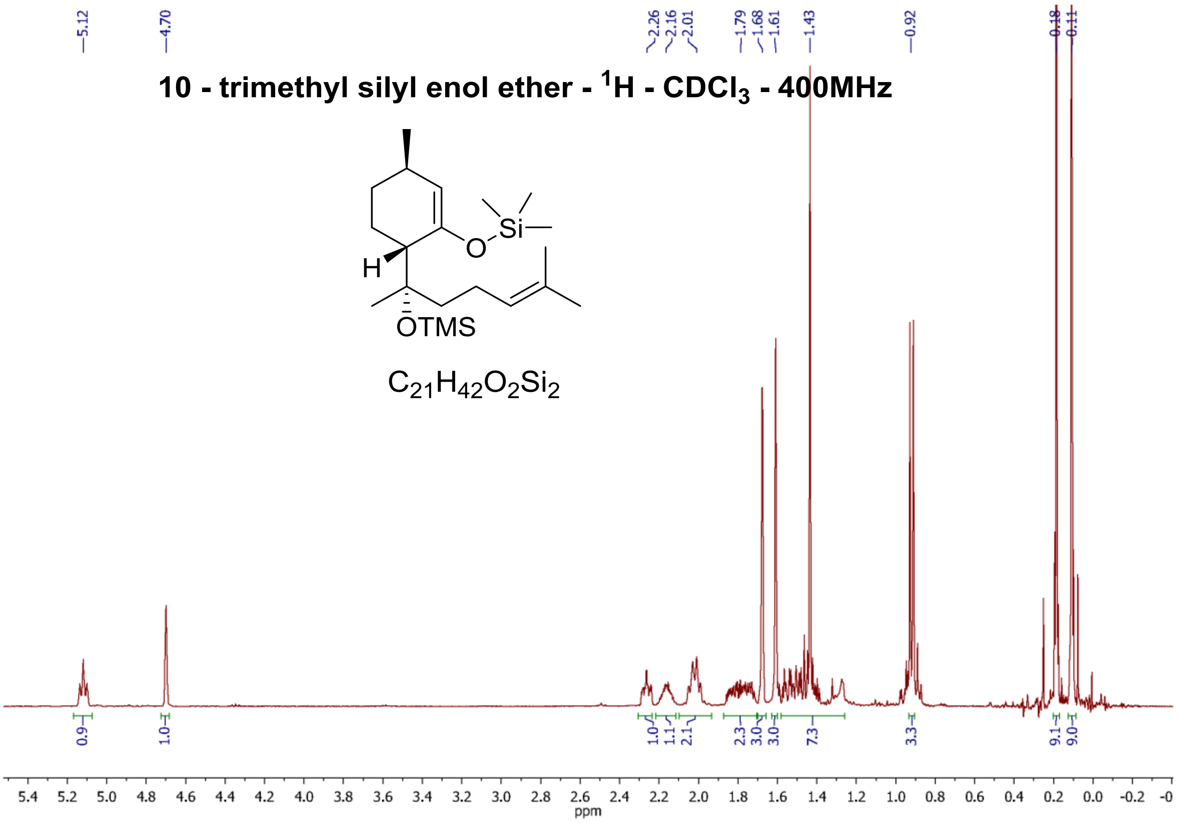
10 - trimethyl silyl enol ether - ^{1}H - $CDCl_3$ - 400MHz
5.12
4.70
2.26
2.16
2.01
1.79
1.68
1.61
1.43
0.92
0.18
0.11
H
O
Si
OTMS
$C_{21}H_{42}O_2Si_2$
0.9
1.0
1.0
1.1
2.1
2.3
3.0
3.0
7.3
3.3
9.1
9.0
ppm

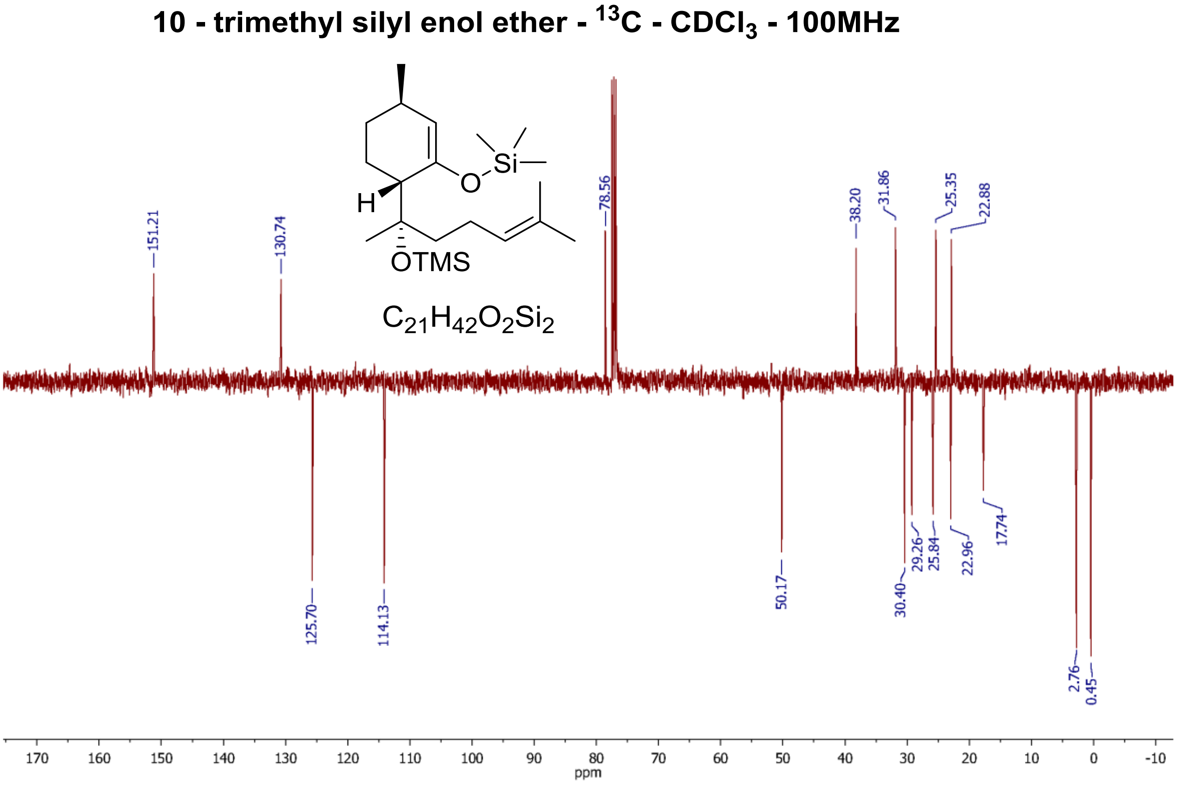
10 - trimethyl silyl enol ether - ^{13}C - $CDCl_3$ - 100MHz
151.21
130.74
78.56
38.20
31.86
25.35
22.88
H
O
Si
OTMS
$C_{21}H_{42}O_2Si_2$
125.70
114.13
50.17
30.40
29.26
25.84
22.96
17.74
2.76
0.45
ppm

11 - silyl hernandulcin - ^{1}H - $CDCl_3$ - 400MHz

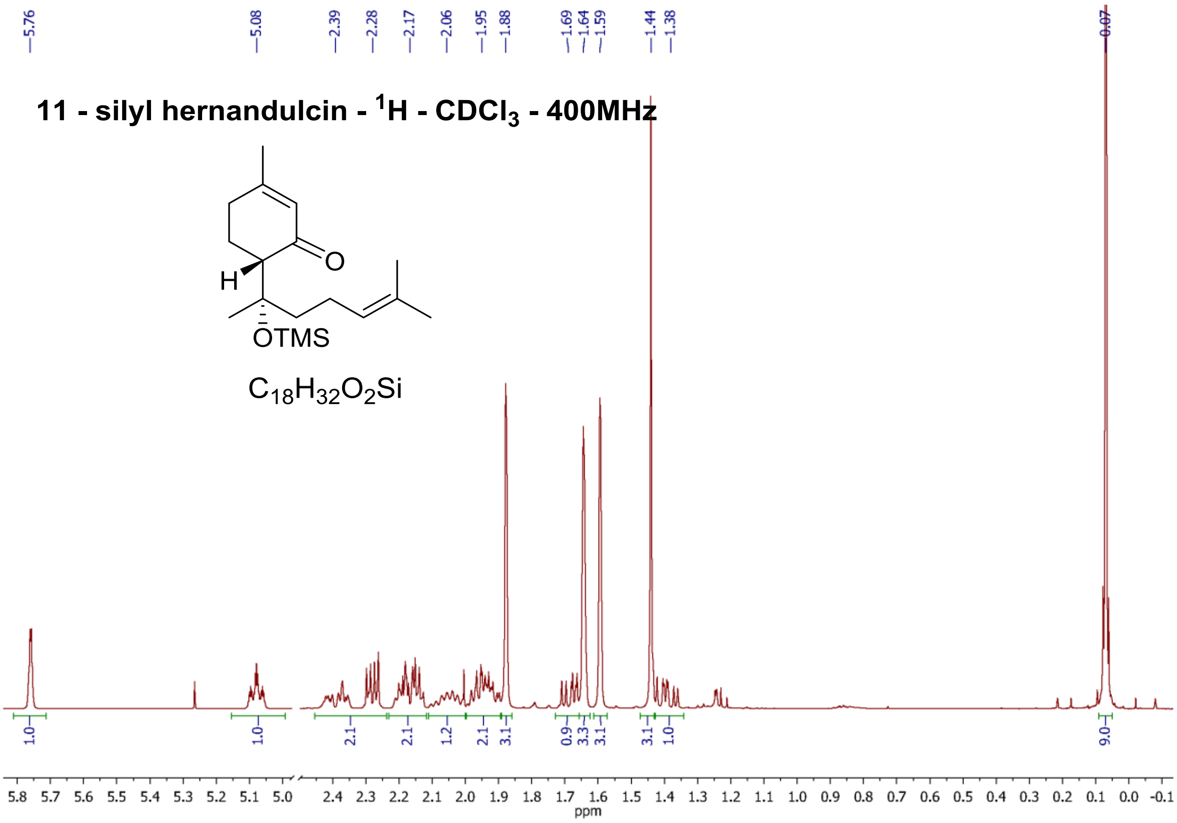

11 - silyl hernandulcin - ^{13}C - $CDCl_3$ - 100MHz

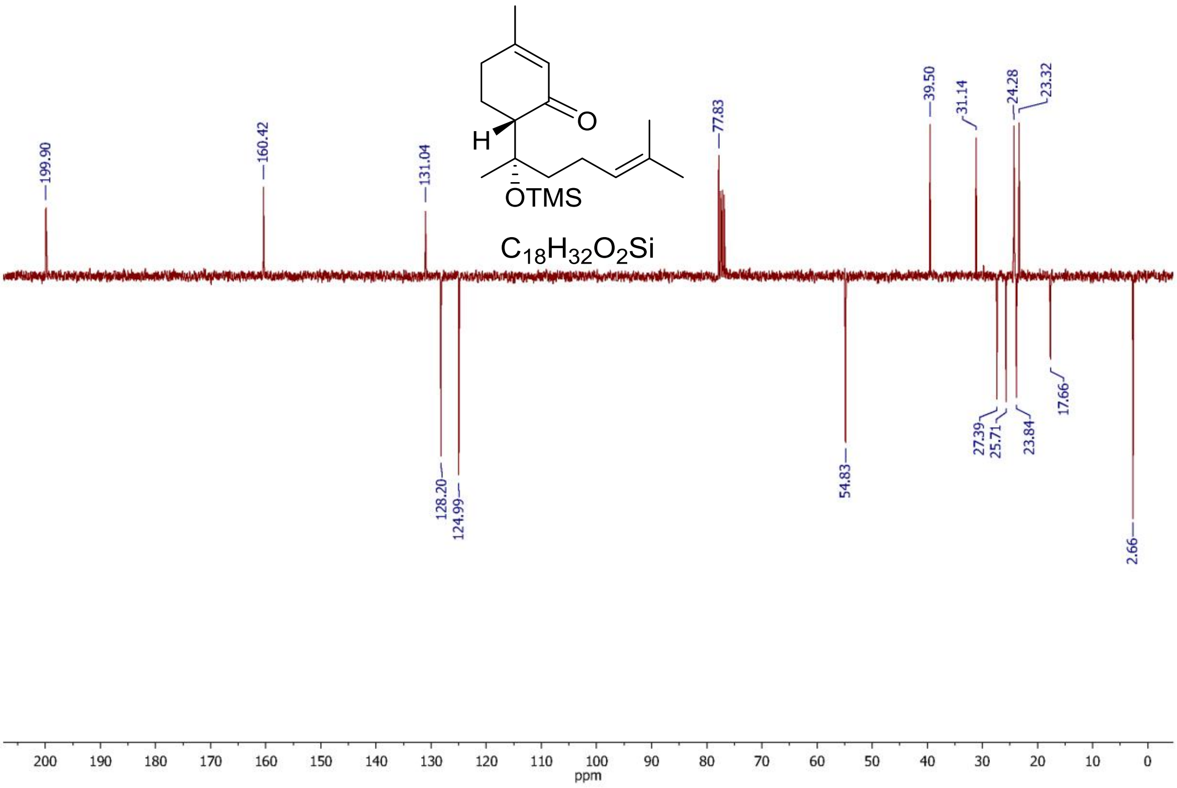

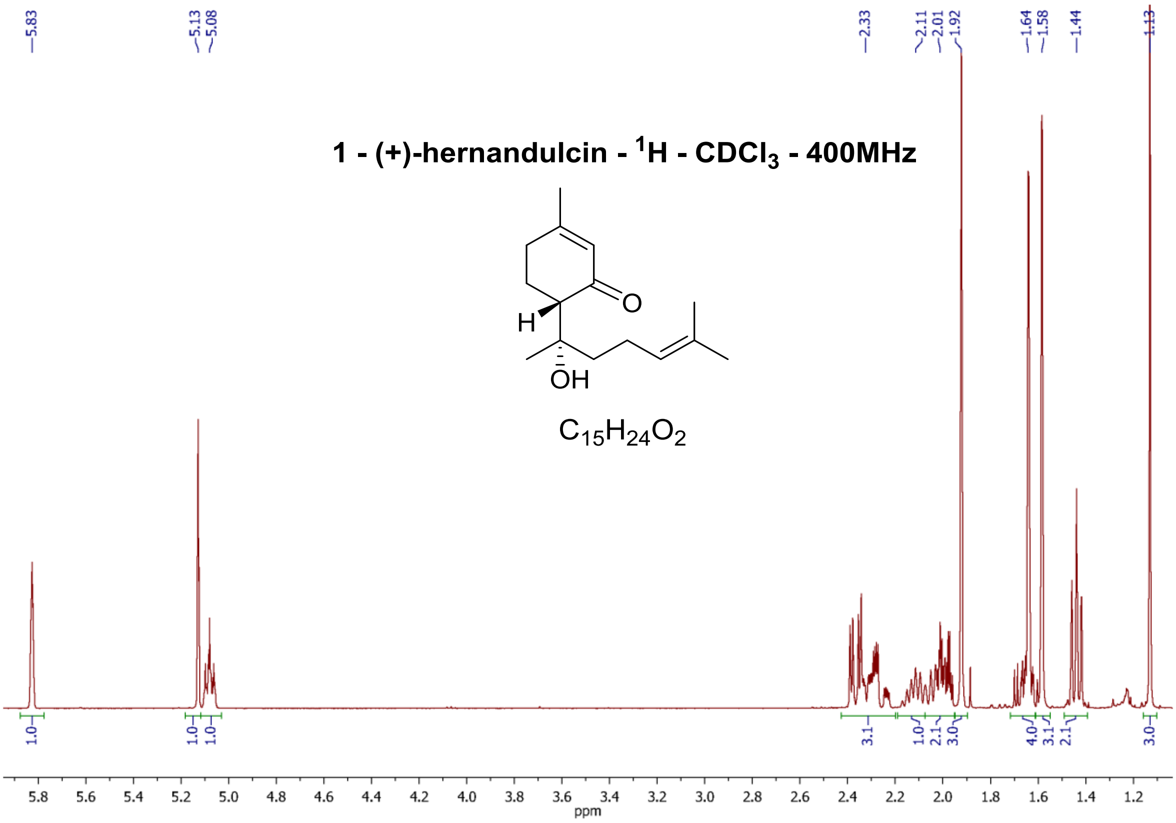
1 - (+)-hernandulcin - ^{1}H - $CDCl_3$ - 400MHz
H
O
OH
$C_{15}H_{24}O_2$
5.83
5.13
5.08
2.33
2.11
2.01
1.92
1.64
1.58
1.44
1.13
1.0
1.0
1.0
3.1
1.0
2.1
3.0
4.0
3.1
2.1
3.0
ppm

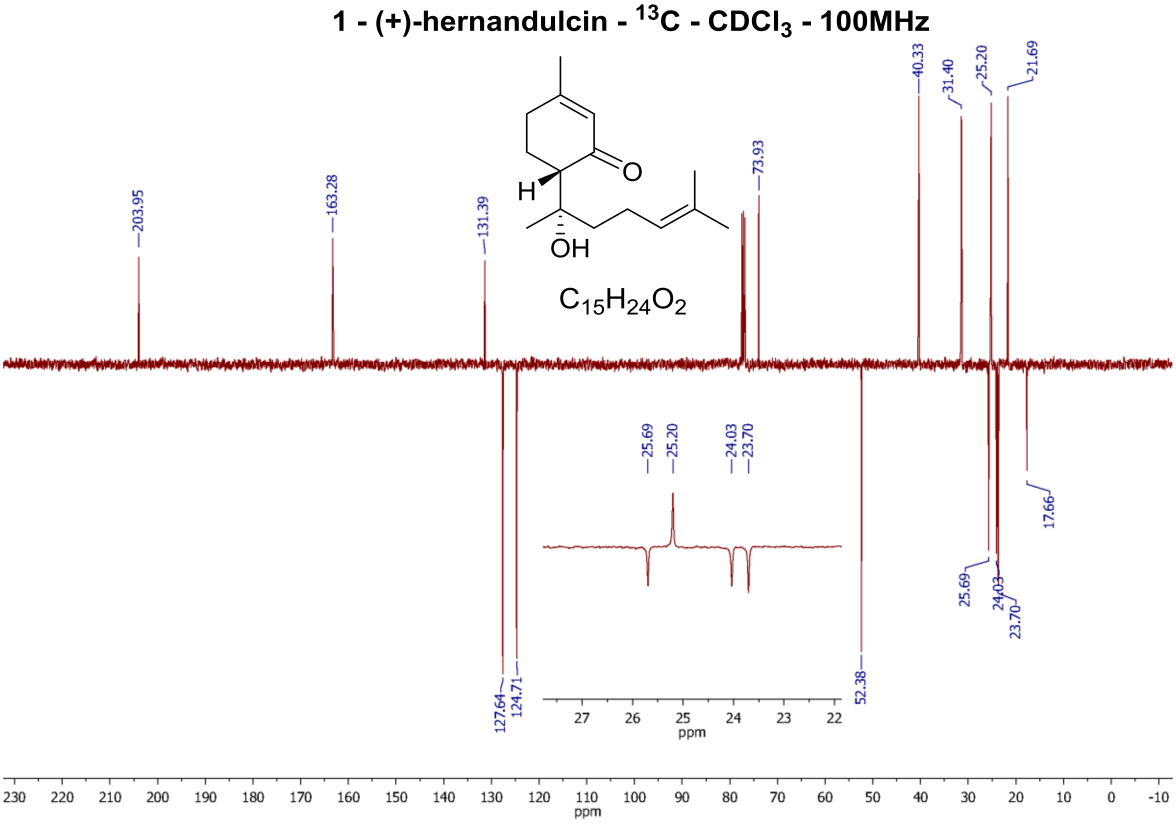
1 - (+)-hernandulcin - ^{13}C - $CDCl_3$ - 100MHz
H
O
OH
$C_{15}H_{24}O_2$
203.95
163.28
131.39
127.64
124.71
73.93
52.38
40.33
31.40
25.69
25.20
24.03
23.70
21.69
17.66
ppm

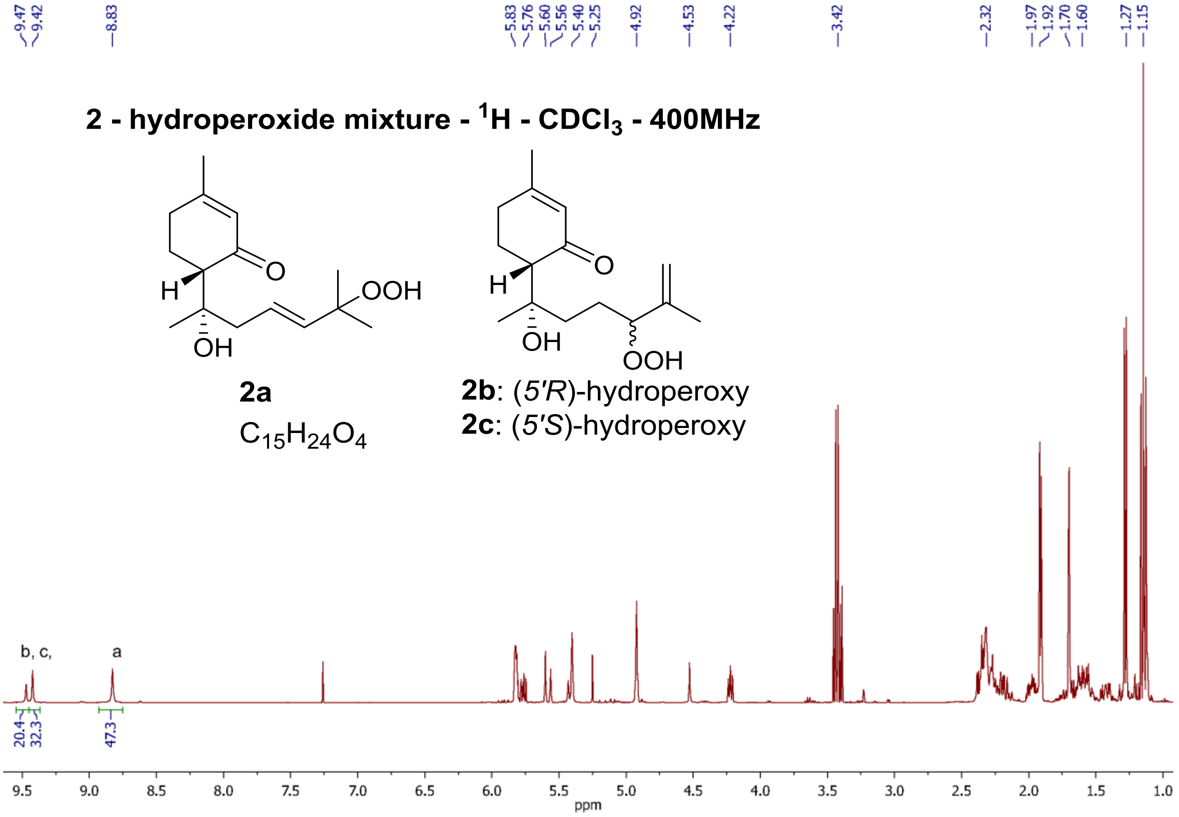
9.47
9.42
8.83
5.83
5.76
5.60
5.56
5.40
5.25
4.92
4.53
4.22
3.42
2.32
1.97
1.92
1.70
1.60
1.27
1.15
2 - hydroperoxide mixture - 1H - CDCl3 - 400MHz
H
O
OOH
OH
2a
C15H24O4
H
O
OH
OOH
2b: (5'R)-hydroperoxy
2c: (5'S)-hydroperoxy
b, c,
a
20.4
32.3
47.3
9.5
9.0
8.5
8.0
7.5
7.0
6.5
6.0
5.5
5.0
4.5
4.0
3.5
3.0
2.5
2.0
1.5
1.0
ppm

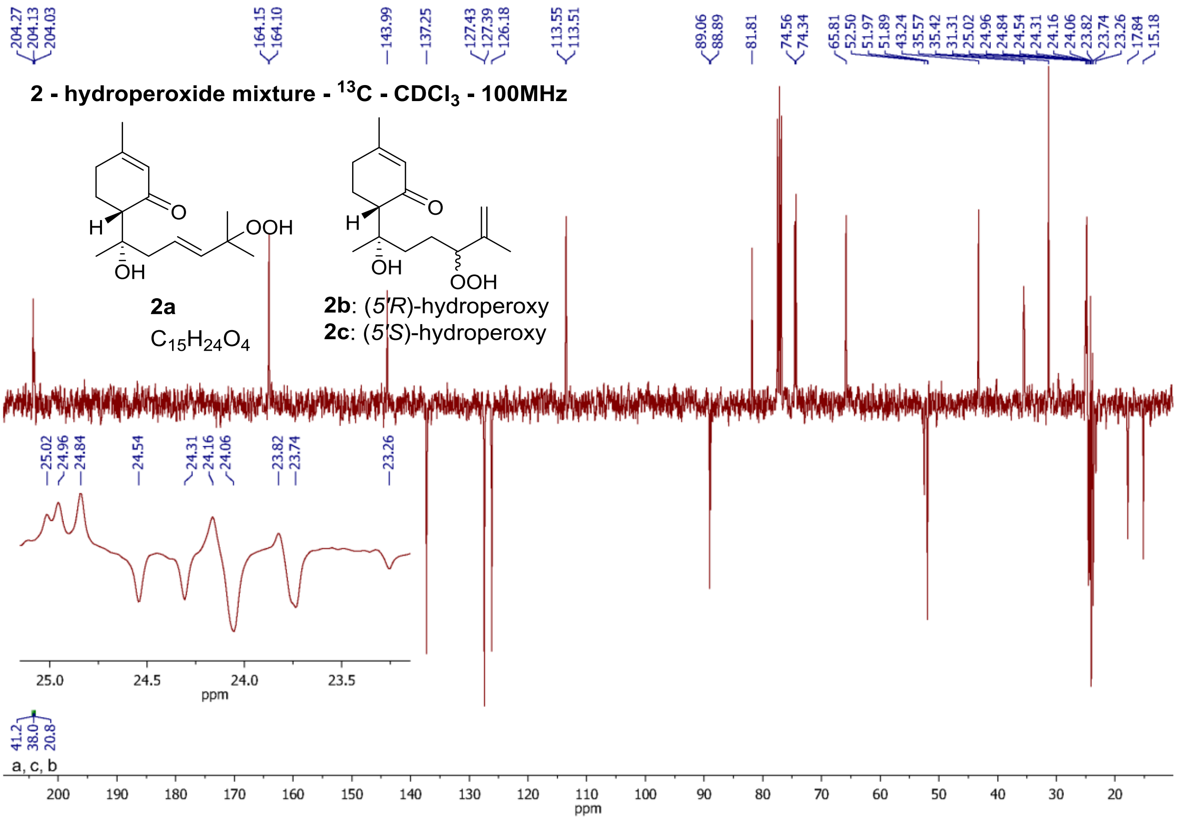
204.27
204.13
204.03
164.15
164.10
143.99
137.25
127.43
127.39
126.18
113.55
113.51
89.06
88.89
81.81
74.56
74.34
65.81
52.50
51.97
51.89
43.24
35.57
35.42
31.31
25.02
24.96
24.84
24.54
24.31
24.16
24.06
23.82
23.74
23.26
17.84
15.18
2 - hydroperoxide mixture - 13C - CDCl3 - 100MHz
H
O
OOH
OH
2a
C15H24O4
H
O
OH
OOH
2b: (5'R)-hydroperoxy
2c: (5'S)-hydroperoxy
25.02
24.96
24.84
24.54
24.31
24.16
24.06
23.82
23.74
23.26
25.0
24.5
24.0
23.5
ppm
41.2
38.0
20.8
a, c, b
200
190
180
170
160
150
140
130
120
110
100
90
80
70
60
50
40
30
20
ppm

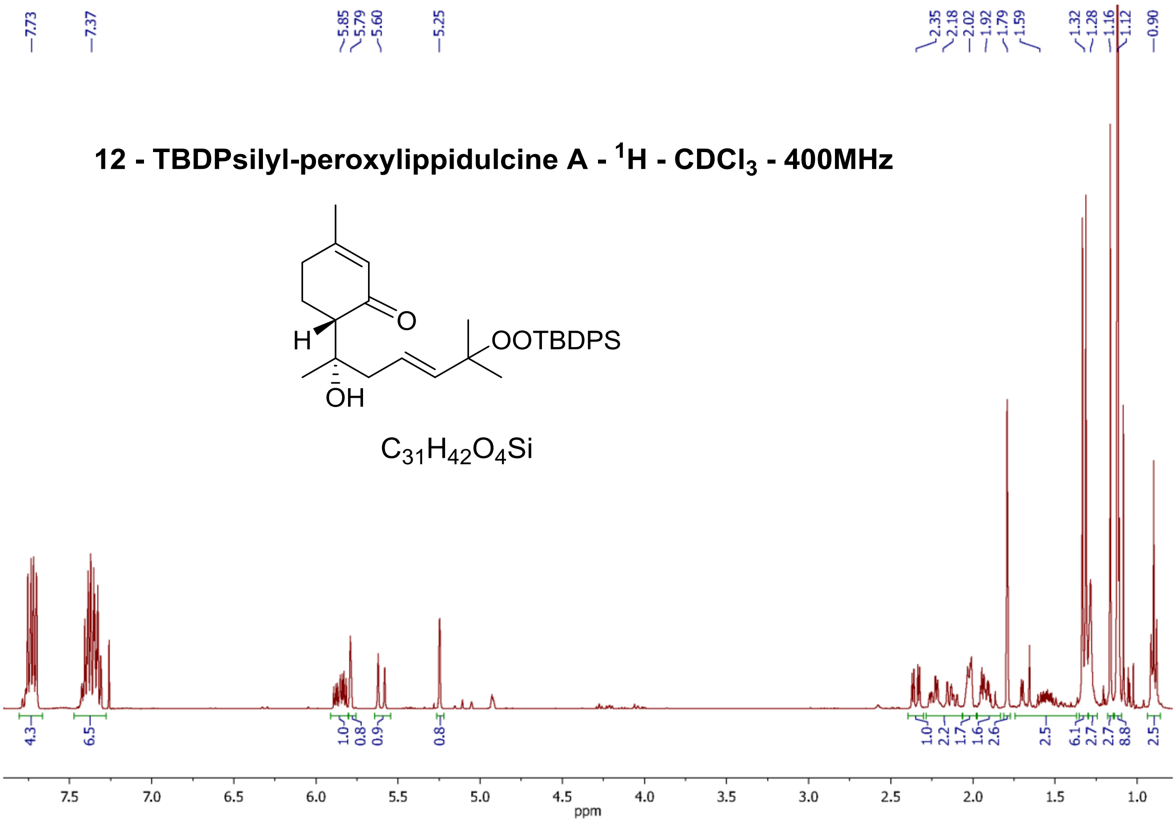
7.73
7.37
5.85
5.79
5.60
5.25
2.35
2.18
2.02
1.92
1.79
1.59
1.32
1.28
1.16
1.12
0.90
12 - TBDPsilyl-peroxylippidulcine A - 1H - CDCl3 - 400MHz
O
H
OOTBDPS
OH
C31H42O4Si
4.3
6.5
1.0
0.8
0.9
0.8
1.0
2.2
1.7
1.6
2.6
2.5
6.1
2.7
2.7
8.8
2.5
ppm

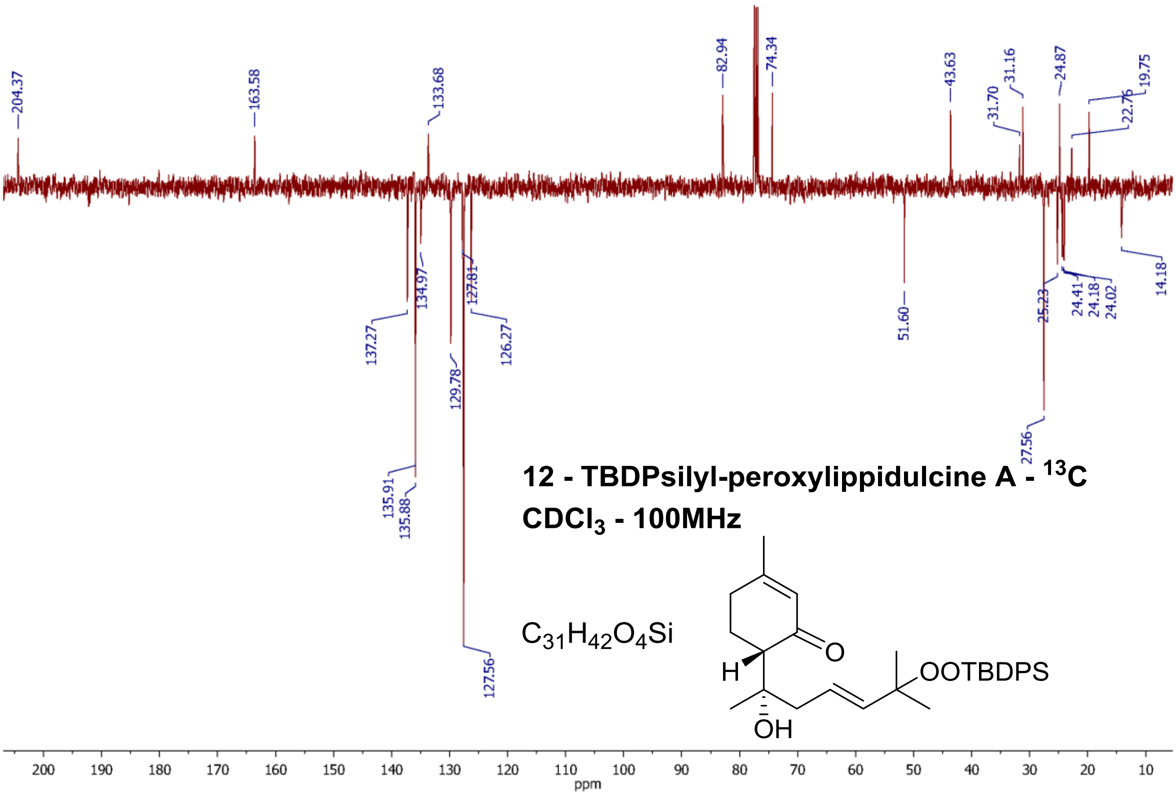
204.37
163.58
133.68
82.94
74.34
43.63
31.70
31.16
24.87
22.76
19.75
137.27
134.97
127.81
126.27
129.78
135.91
135.88
127.56
51.60
25.23
24.41
24.18
24.02
14.18
27.56
12 - TBDPsilyl-peroxylippidulcine A - 13C
CDCl3 - 100MHz
C31H42O4Si
O
H
OOTBDPS
OH
ppm

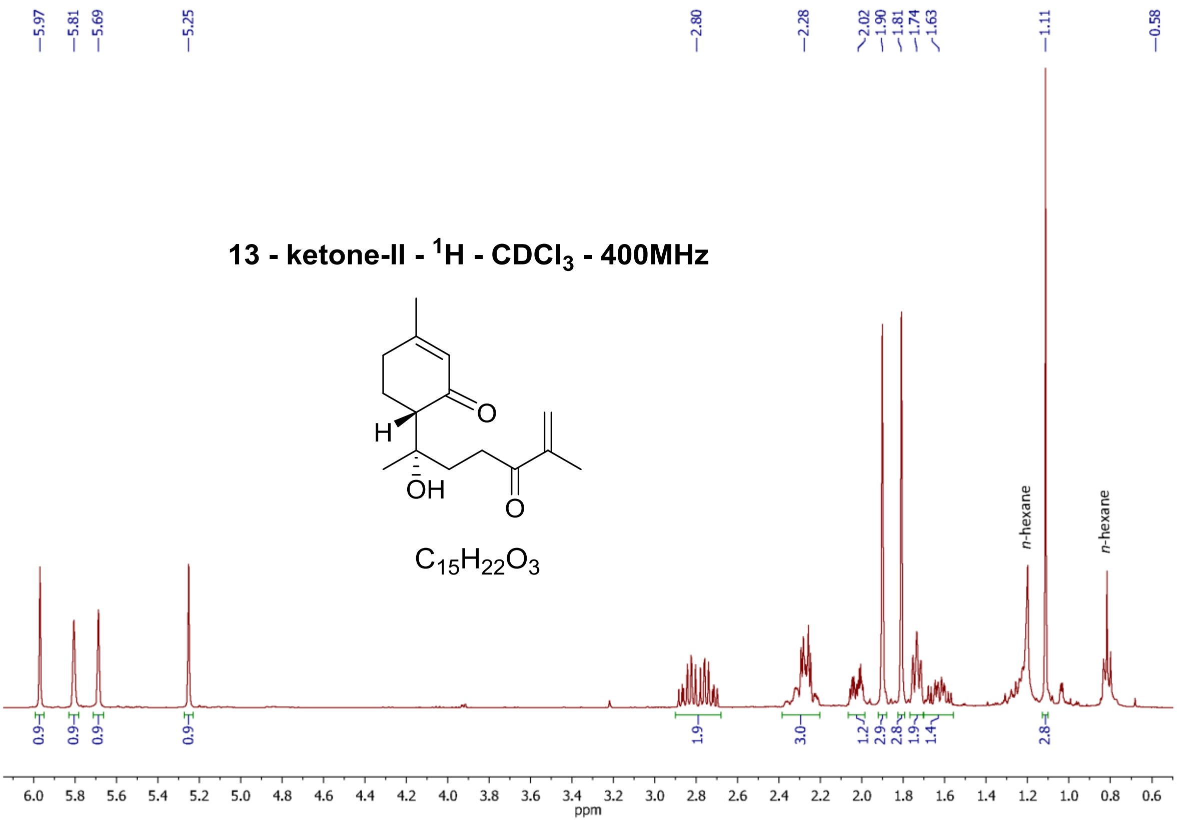
13 - ketone-II - ^{1}H - $CDCl_3$ - 400MHz
$C_{15}H_{22}O_3$
H
OH
O
O
n-hexane
n-hexane
5.97
5.81
5.69
5.25
2.80
2.28
2.02
1.90
1.81
1.74
1.63
1.11
0.58
ppm

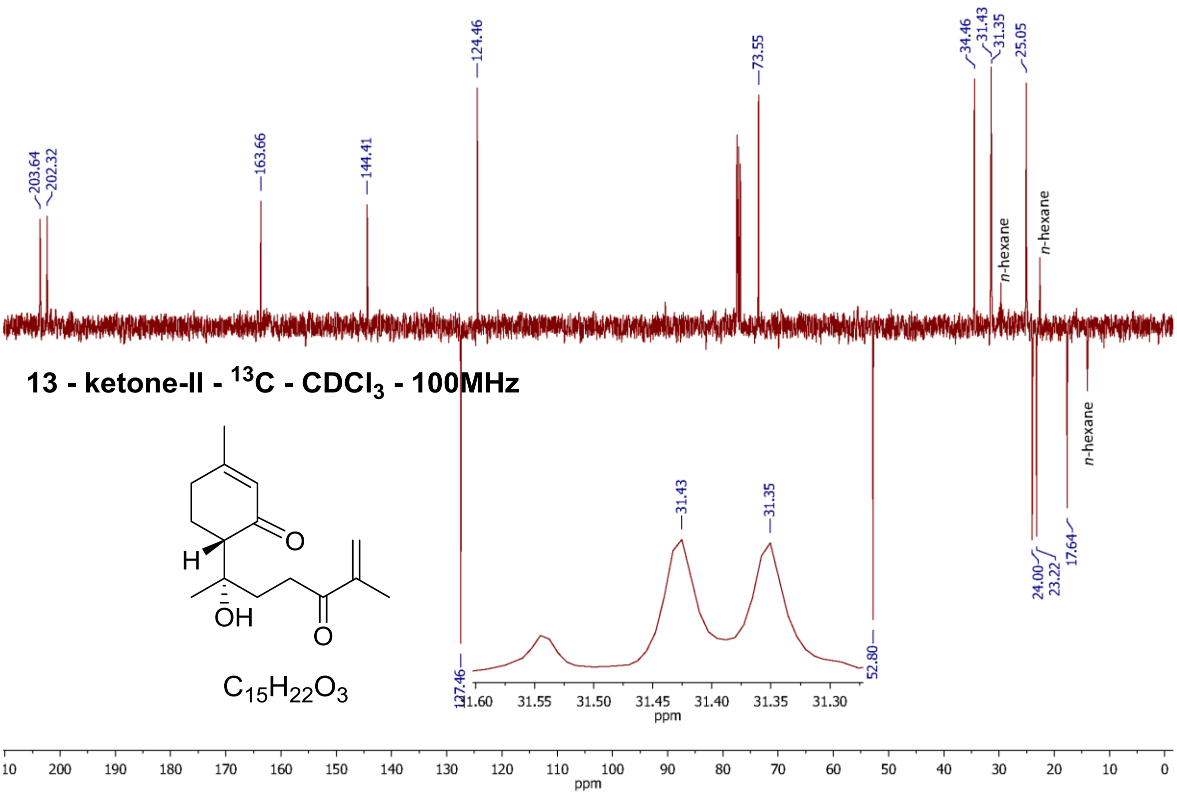
13 - ketone-II - ^{13}C - $CDCl_3$ - 100MHz
$C_{15}H_{22}O_3$
H
OH
O
O
203.64
202.32
163.66
144.41
124.46
73.55
34.46
31.43
31.35
25.05
127.46
52.80
24.00
23.22
17.64
n-hexane
n-hexane
n-hexane
ppm

2a - peroxylippidulcine A - ^{1}H - $CDCl_3$ - 400MHz

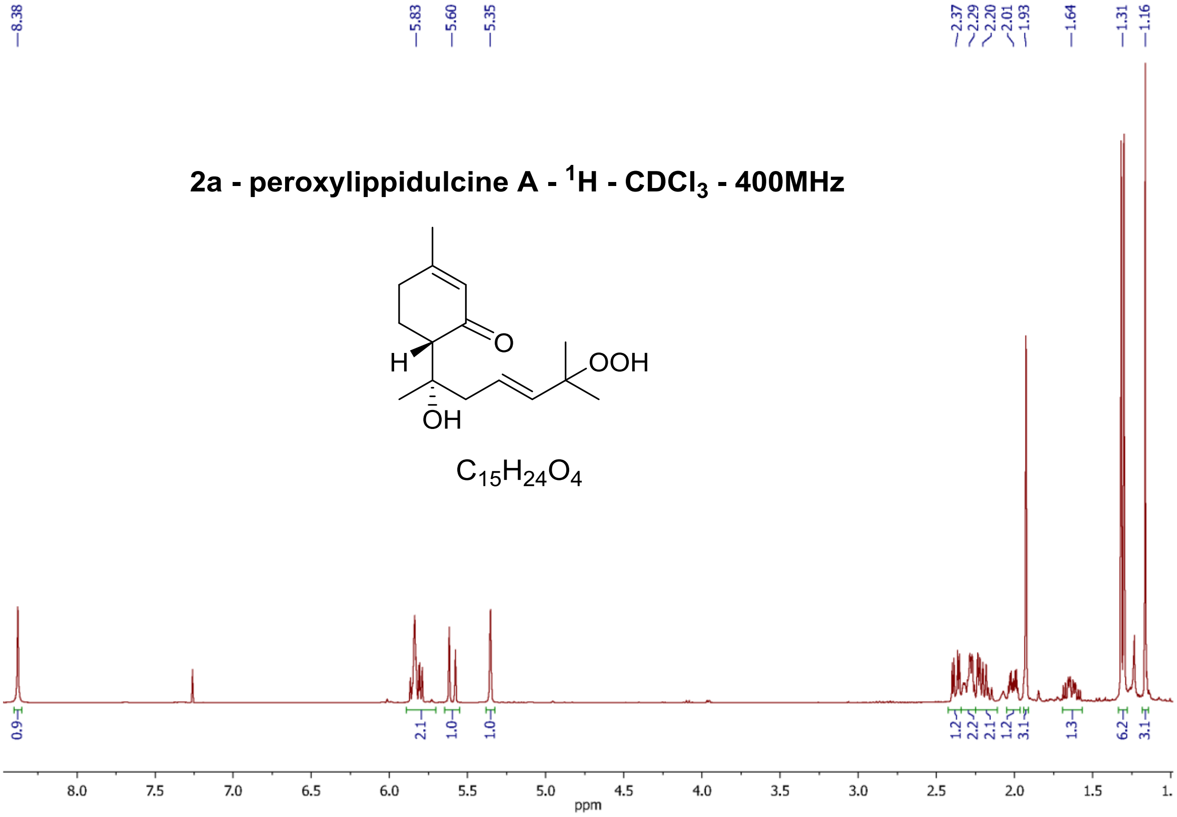

2a - peroxylippidulcine A - ^{13}C - $CDCl_3$ - 100MHz

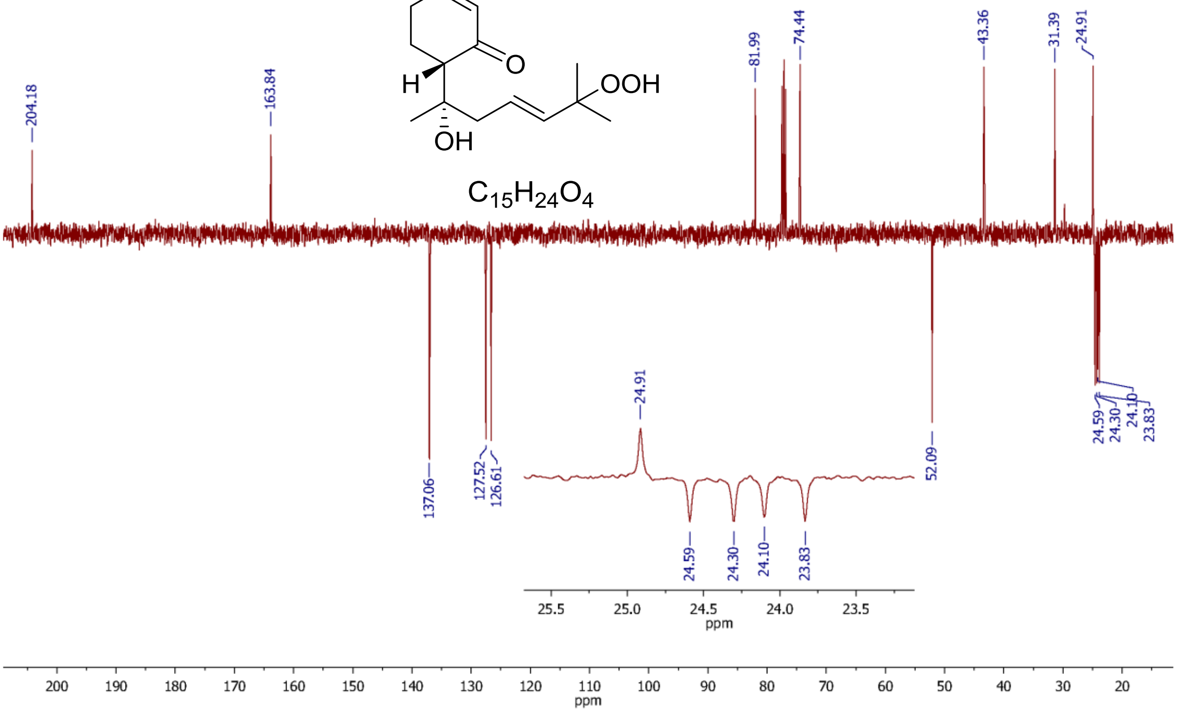

15a - lippidulcine A-OTMS - ^{1}H - $CDCl_3$ - 400 MHz

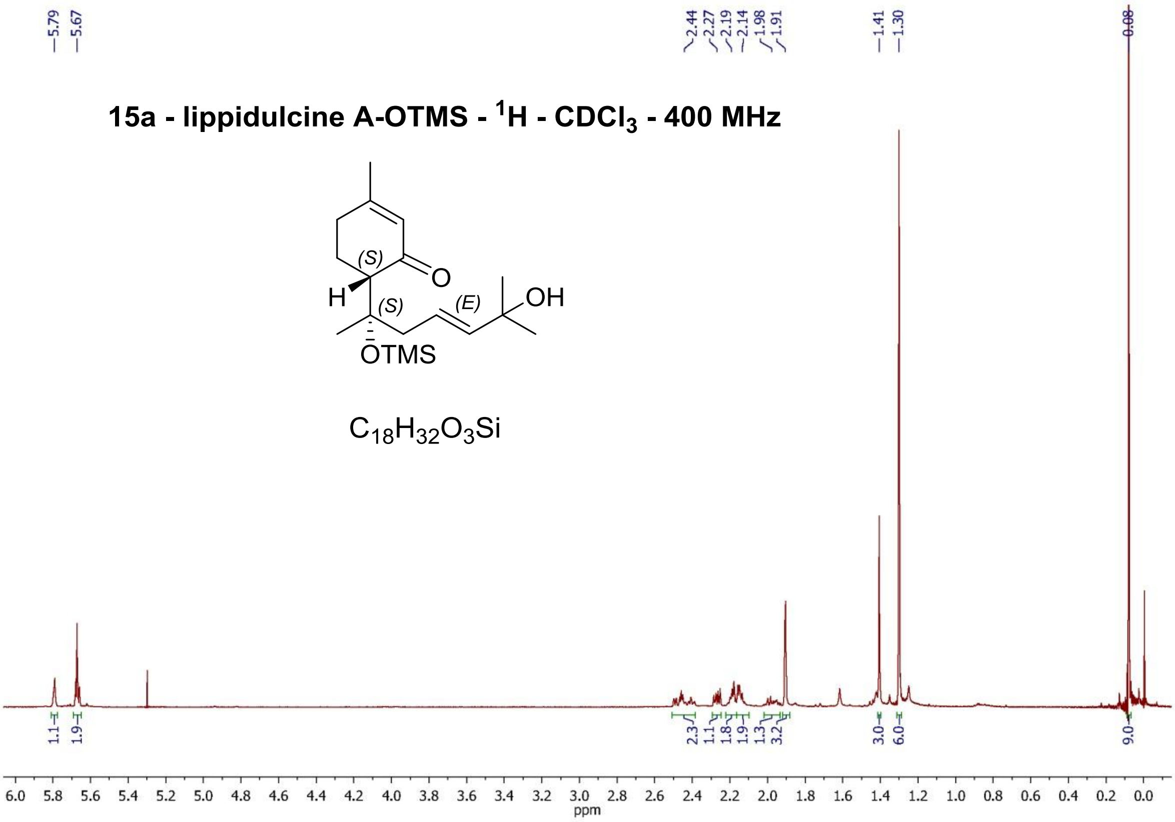

15a - lippidulcine A-OTMS - ^{13}C - $CDCl_3$ - 100 MHz

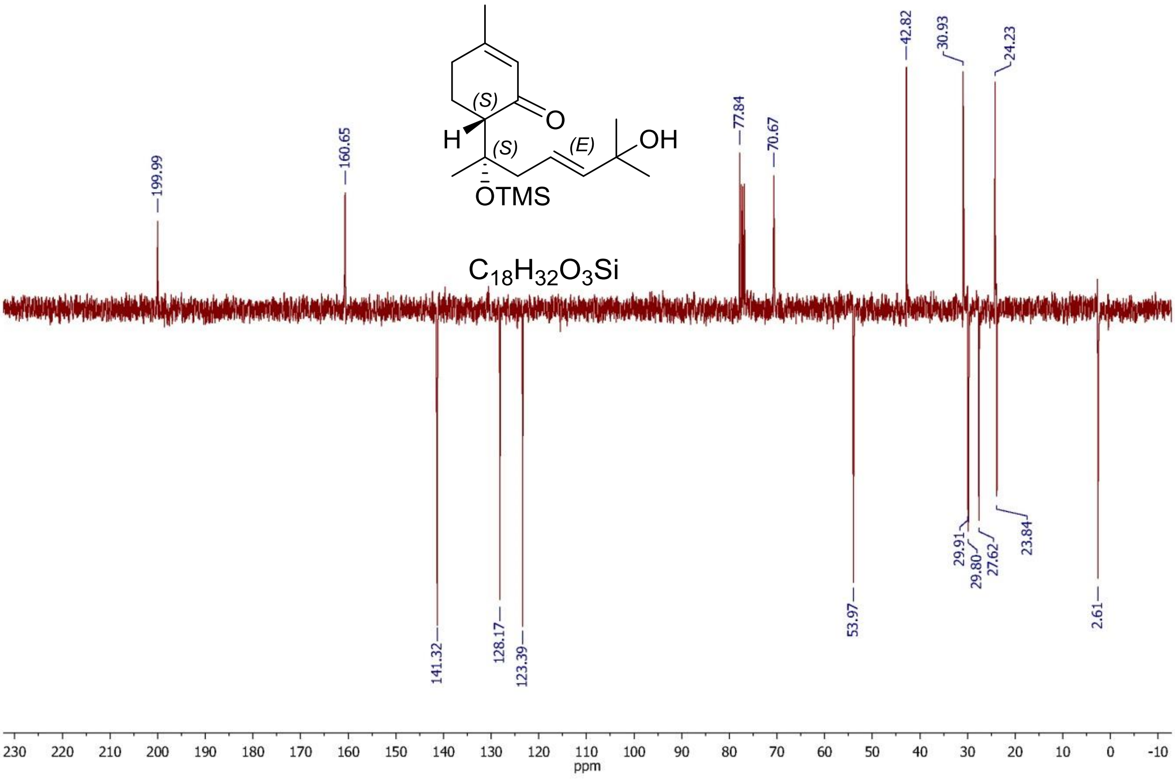

3a - lippidulcine A - ^1H - $CDCl_3$ - 400 MHz

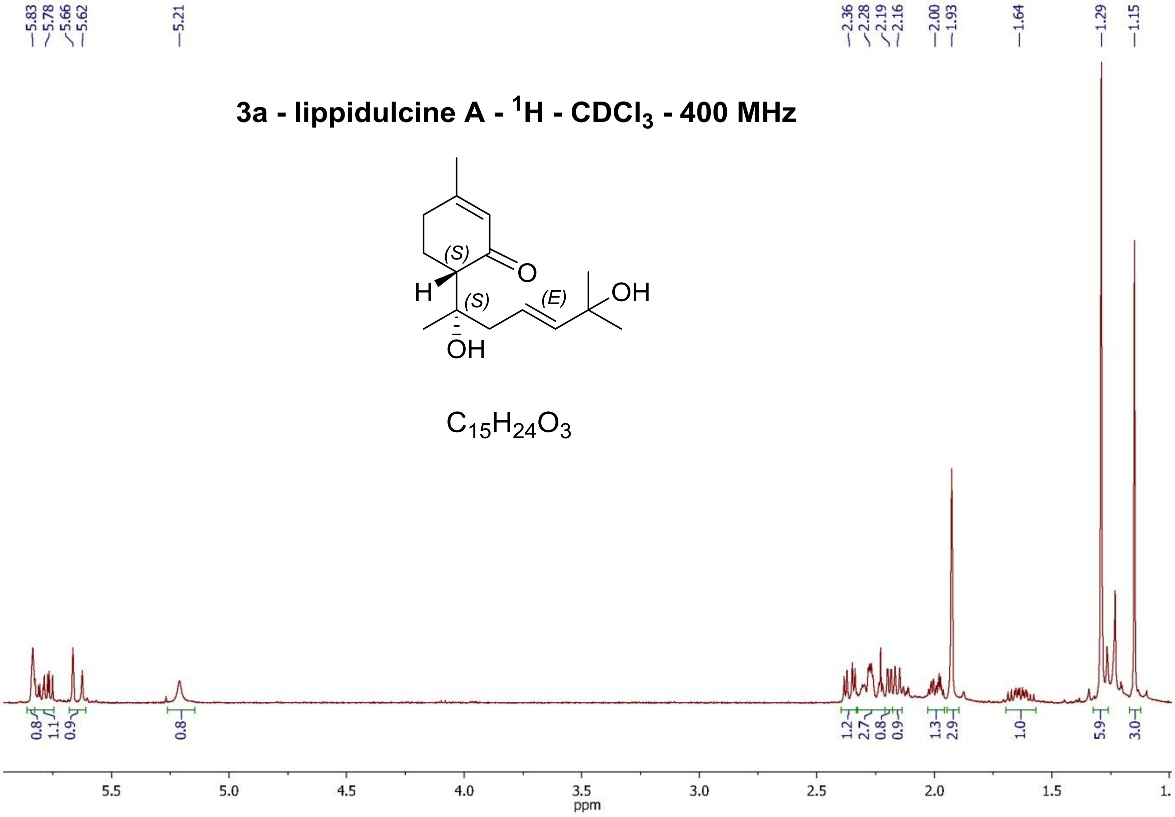

3a - lippidulcine A - ^{13}C - $CDCl_3$ - 100 MHz

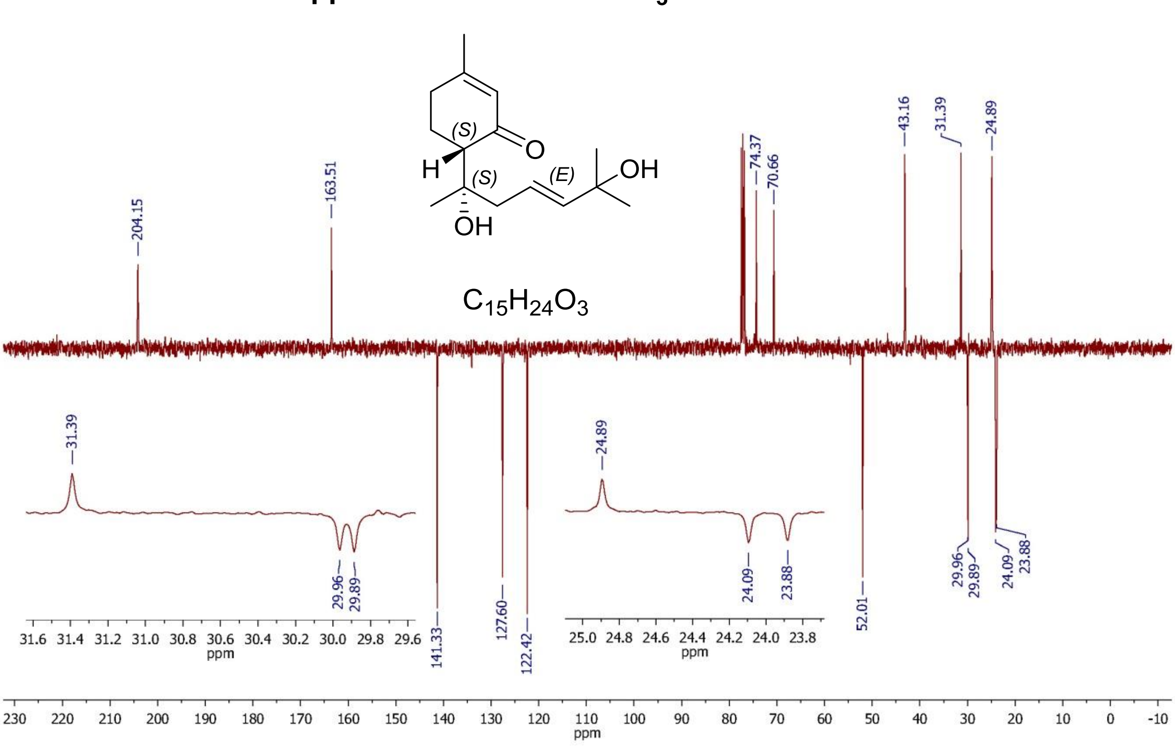

3d - epilippidulcine A - ^{1}H - $CDCl_3$ - 400 MHz

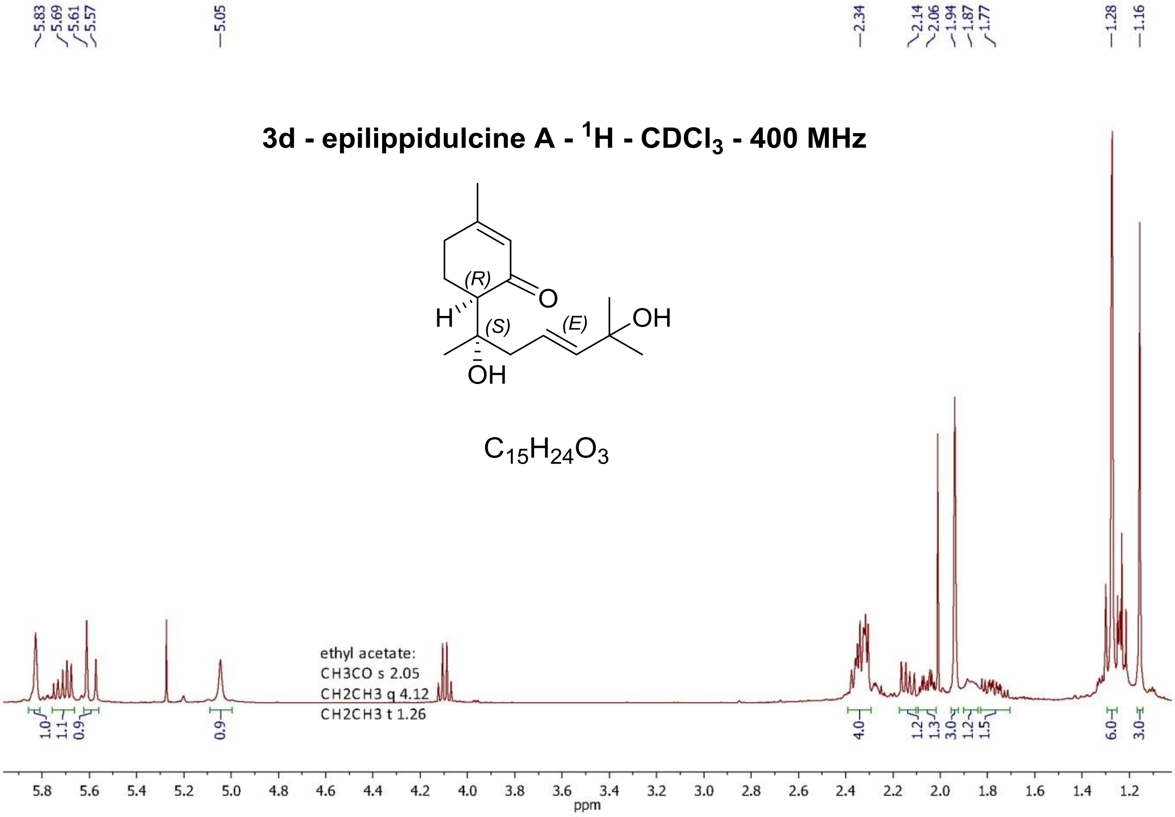

3d - epilippidulcine A - ^{13}C - $CDCl_3$ - 100 MHz

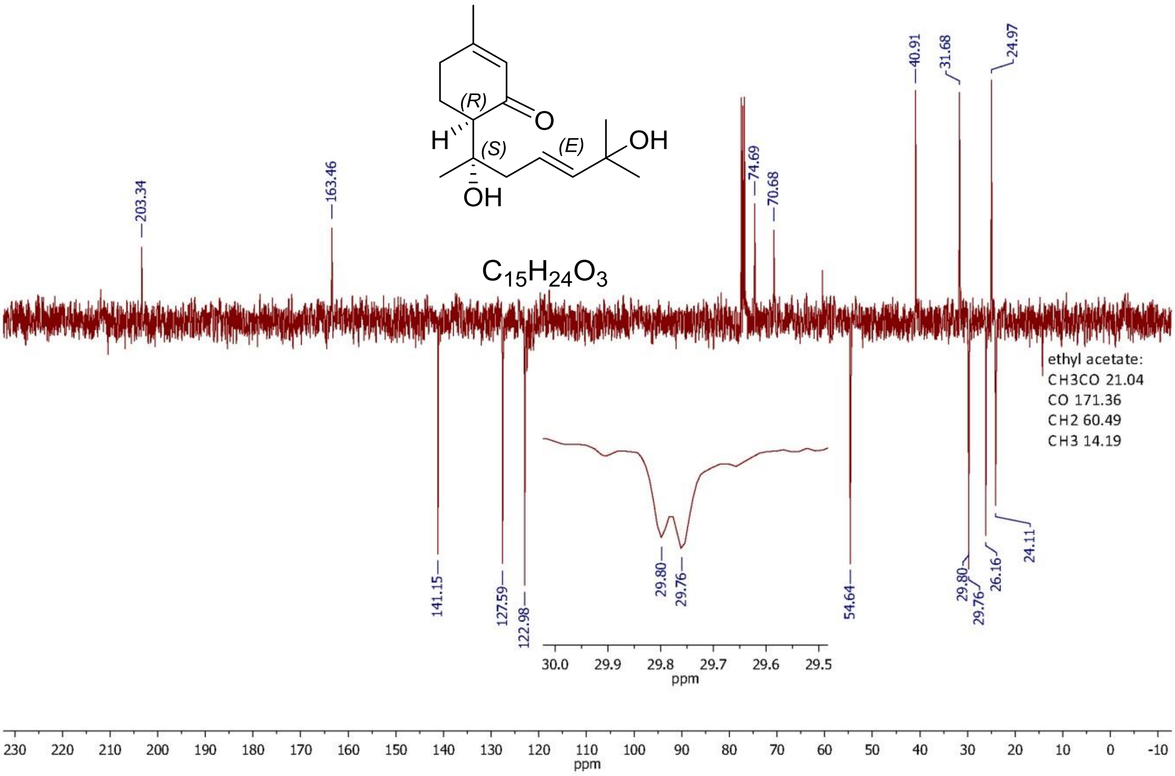

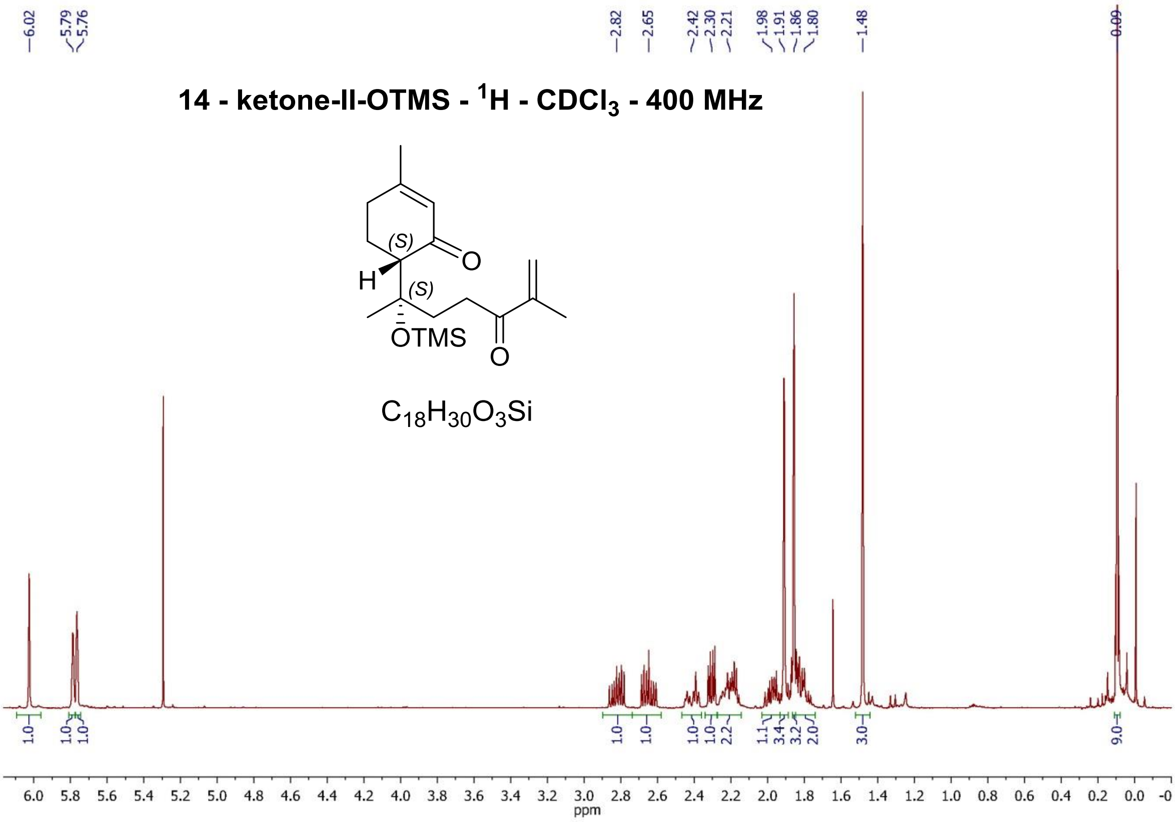
6.02
5.79
5.76
2.82
2.65
2.42
2.30
2.21
1.98
1.91
1.86
1.80
1.48
0.09
14 - ketone-II-OTMS - 1H - CDCl3 - 400 MHz
(S)
H
(S)
OTMS
O
O
C18H30O3Si
1.0
1.0
1.0
1.0
1.0
1.0
1.0
2.2
1.1
3.4
3.2
2.0
3.0
9.0
6.0 5.8 5.6 5.4 5.2 5.0 4.8 4.6 4.4 4.2 4.0 3.8 3.6 3.4 3.2 3.0 2.8 2.6 2.4 2.2 2.0 1.8 1.6 1.4 1.2 1.0 0.8 0.6 0.4 0.2 0.0 -0
ppm

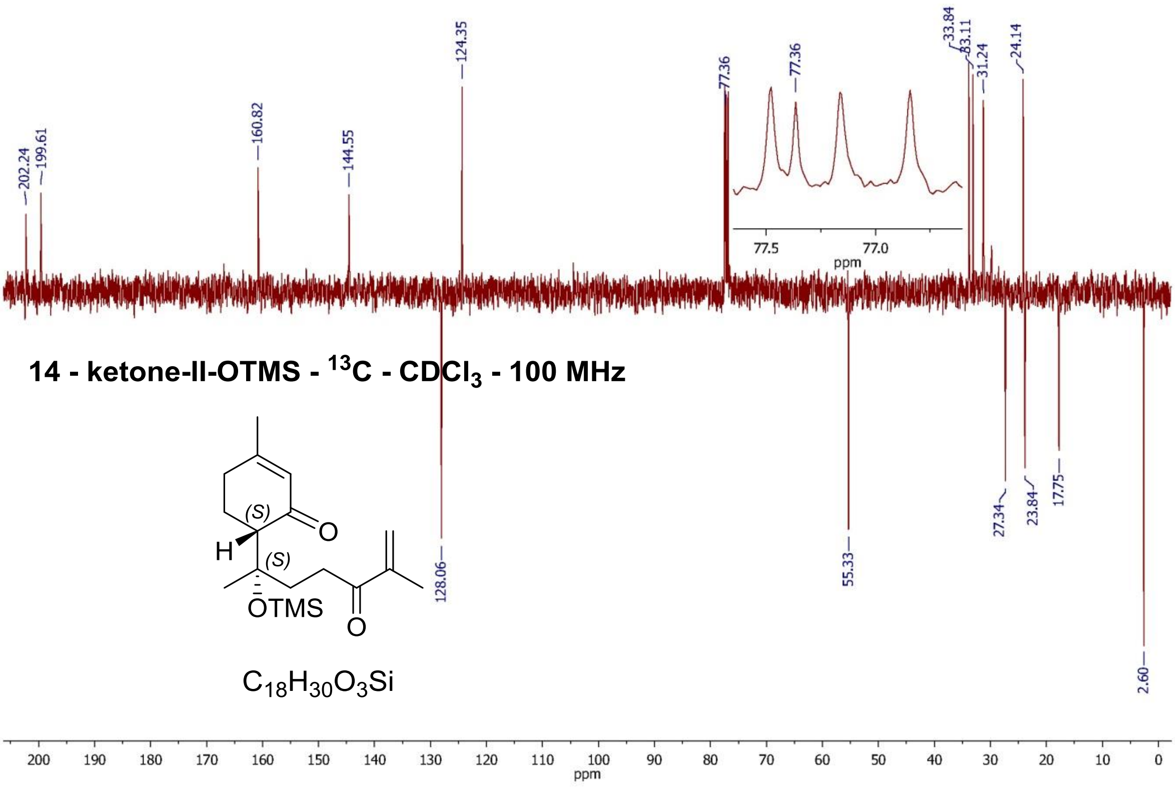
202.24
199.61
160.82
144.55
124.35
77.36
77.36
33.84
33.11
31.24
24.14
77.5
77.0
ppm
14 - ketone-II-OTMS - 13C - CDCl3 - 100 MHz
(S)
H
(S)
OTMS
O
O
C18H30O3Si
128.06
55.33
27.34
23.84
17.75
2.60
200 190 180 170 160 150 140 130 120 110 100 90 80 70 60 50 40 30 20 10 0
ppm

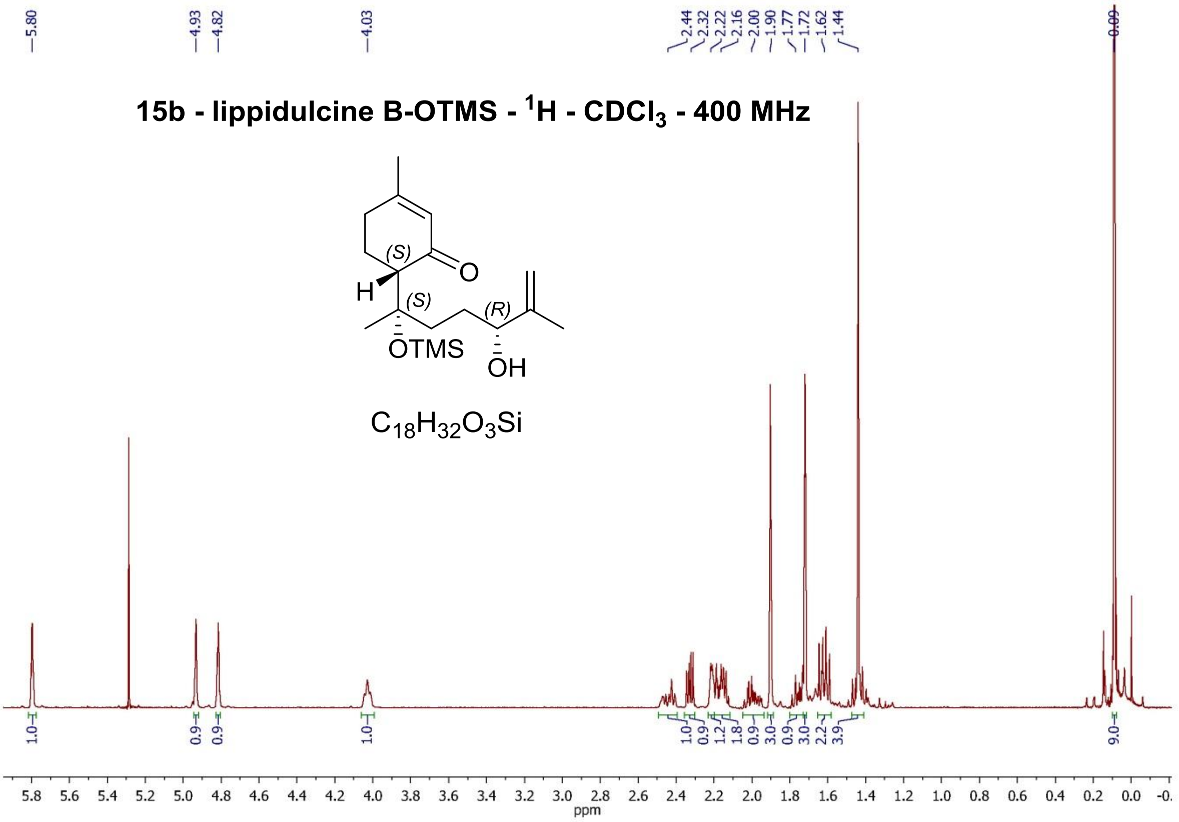
5.80
4.93
4.82
4.03
2.44
2.32
2.22
2.16
2.00
1.90
1.77
1.72
1.62
1.44
0.09
15b - lippidulcine B-OTMS - 1H - CDCl3 - 400 MHz
(S)
O
H
(S)
(R)
OTMS
OH
C18H32O3Si
1.0
0.9
0.9
1.0
1.0
0.9
1.2
1.8
0.9
3.0
0.9
3.0
2.2
3.9
9.0
ppm

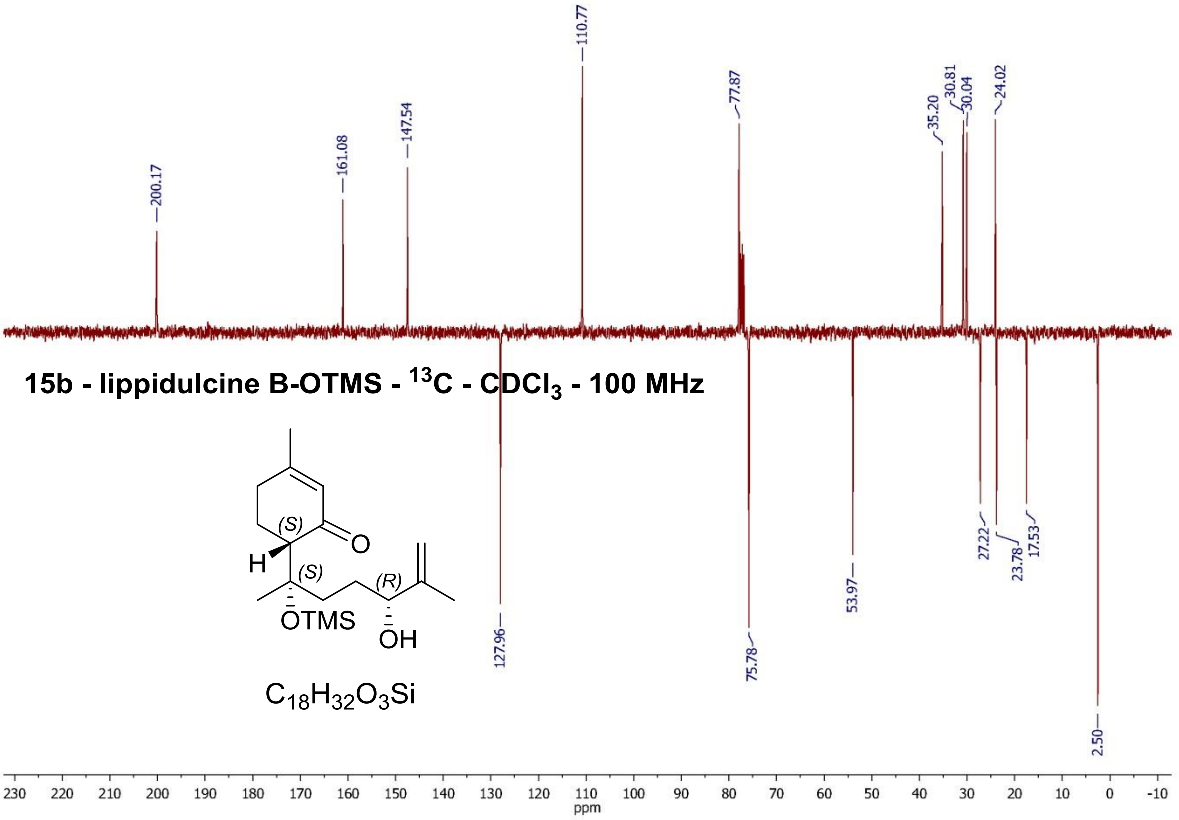
110.77
77.87
35.20
30.81
30.04
24.02
200.17
161.08
147.54
15b - lippidulcine B-OTMS - 13C - CDCl3 - 100 MHz
(S)
O
H
(S)
(R)
OTMS
OH
C18H32O3Si
127.96
75.78
53.97
27.22
23.78
17.53
2.50
ppm

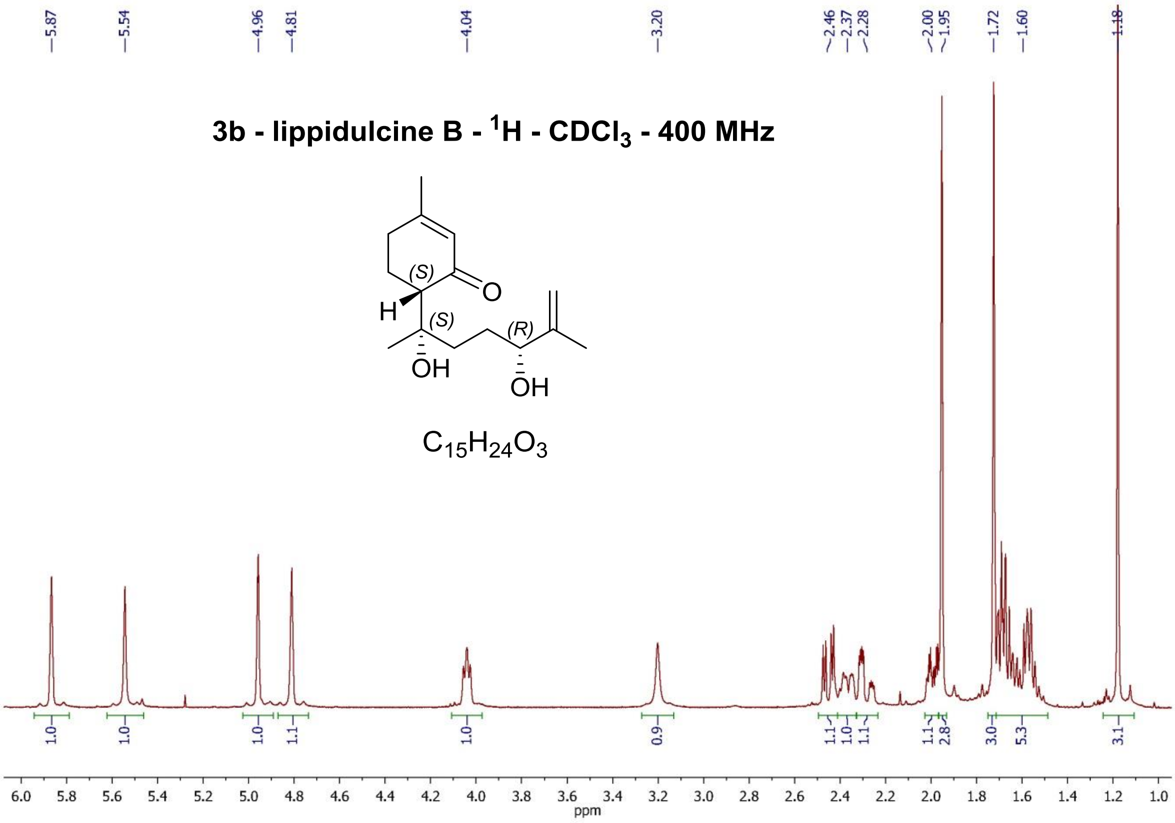

3b - lippidulcine B - 1H - CDCl3 - 400 MHz
(S)
H
(S)
(R)
OH
OH
O
C15H24O3
5.87
5.54
4.96
4.81
4.04
3.20
2.46
2.37
2.28
2.00
1.95
1.72
1.60
1.18
1.0
1.0
1.0
1.1
1.0
0.9
1.1
1.0
1.1
1.1
2.8
3.0
5.3
3.1
ppm

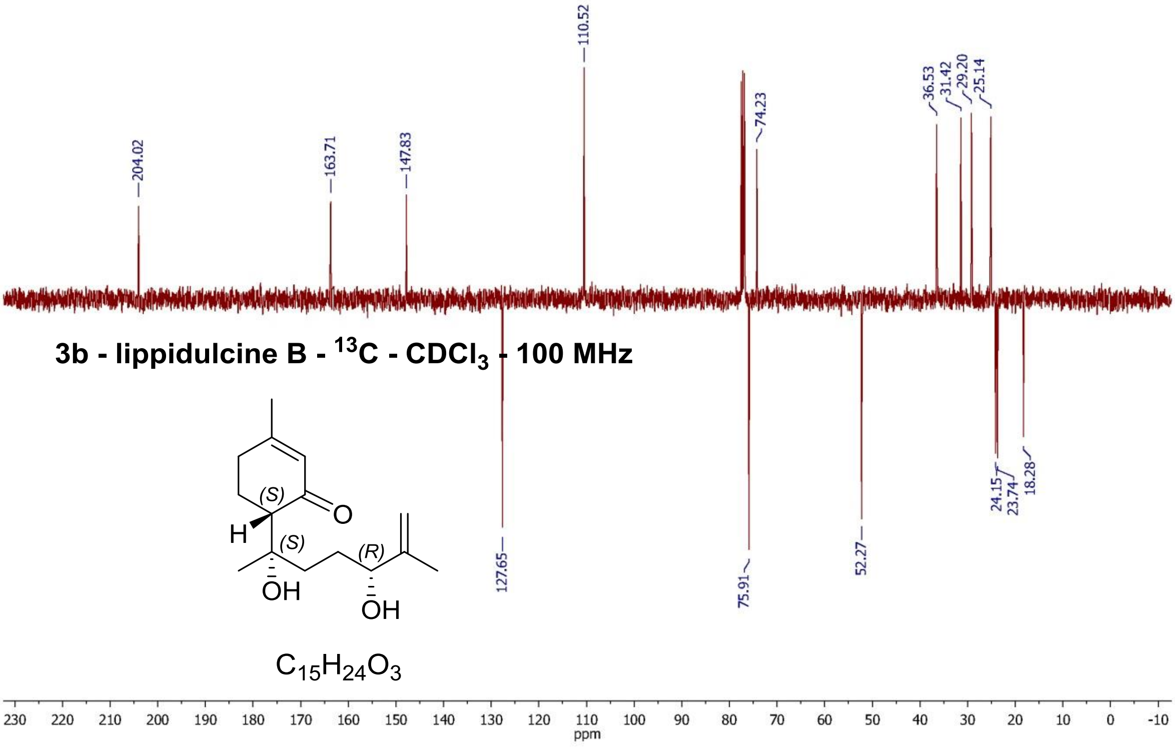

3b - lippidulcine B - 13C - CDCl3 - 100 MHz
(S)
H
(S)
(R)
OH
OH
O
C15H24O3
204.02
163.71
147.83
110.52
74.23
36.53
31.42
29.20
25.14
127.65
75.91
52.27
24.15
23.74
18.28
ppm

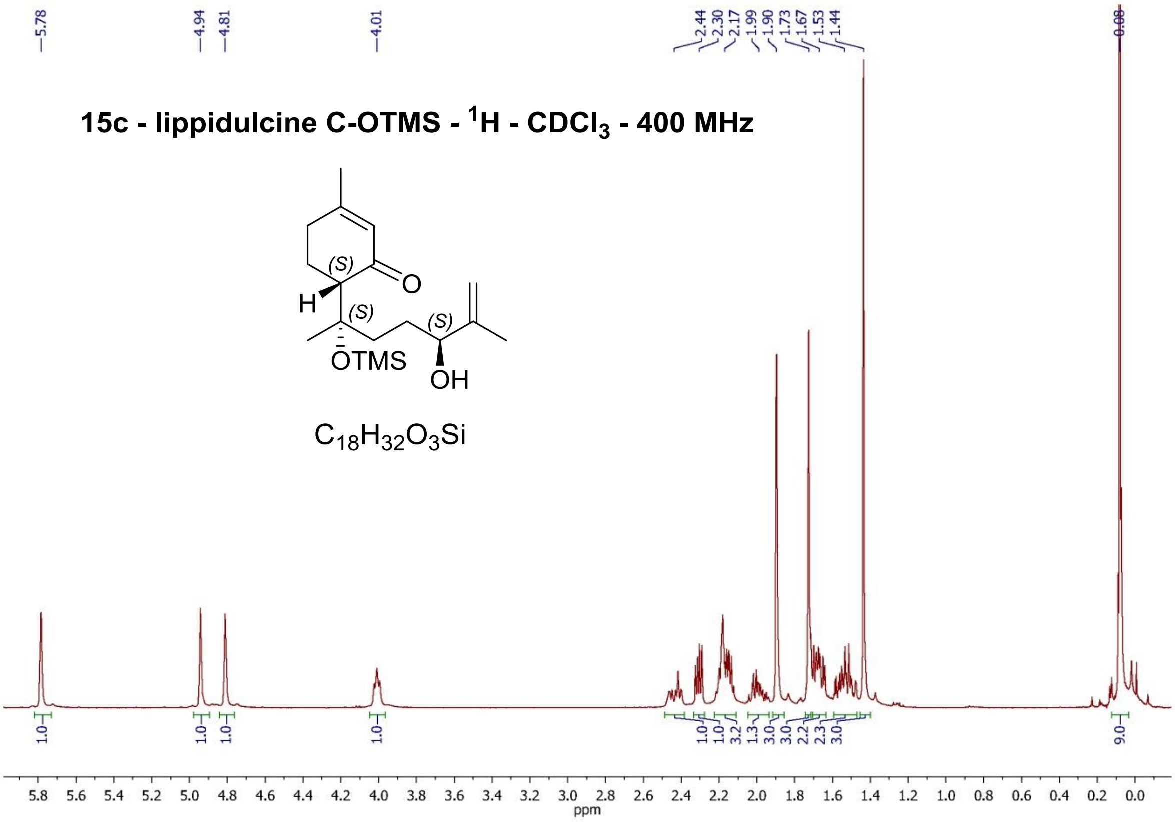
15c - lippidulcine C-OTMS - 1H - CDCl3 - 400 MHz
(S)
(S)
(S)
H
O
OTMS
OH
C18H32O3Si
5.78
4.94
4.81
4.01
2.44
2.30
2.17
1.99
1.90
1.73
1.67
1.53
1.44
0.08
ppm

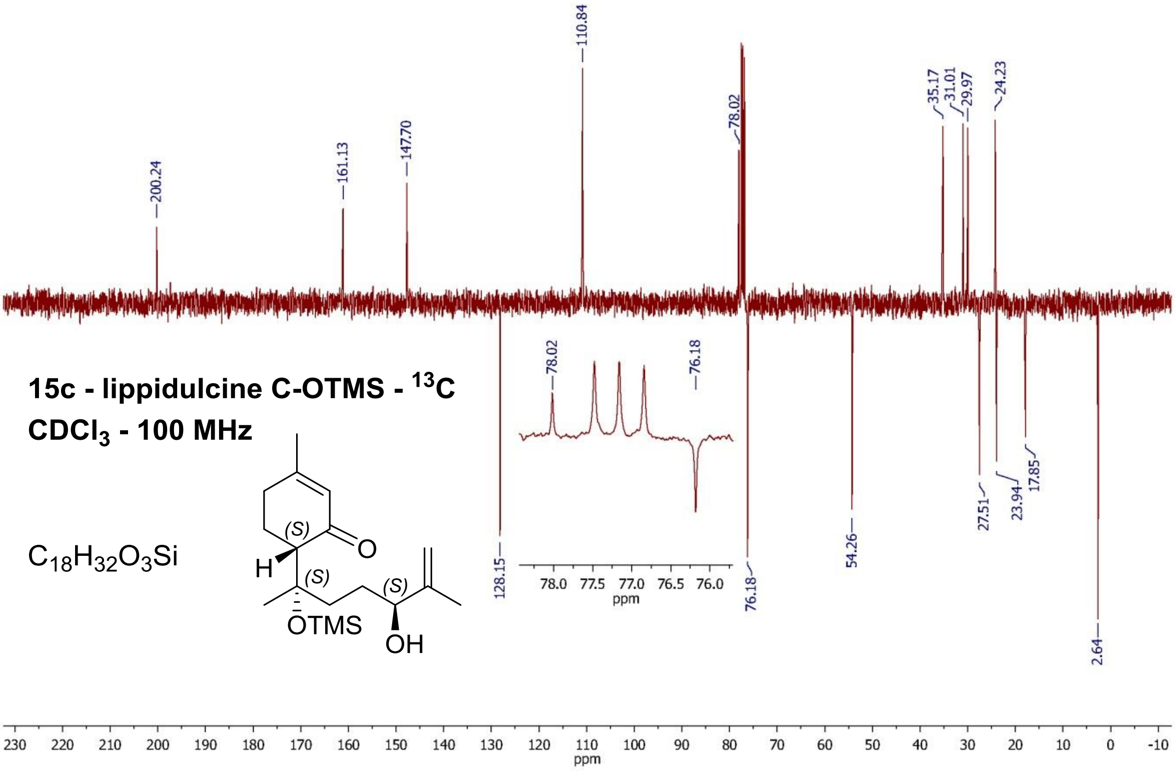
15c - lippidulcine C-OTMS - 13C
CDCl3 - 100 MHz
C18H32O3Si
(S)
(S)
(S)
H
O
OTMS
OH
200.24
161.13
147.70
110.84
78.02
35.17
31.01
29.97
24.23
128.15
76.18
54.26
27.51
23.94
17.85
2.64
ppm

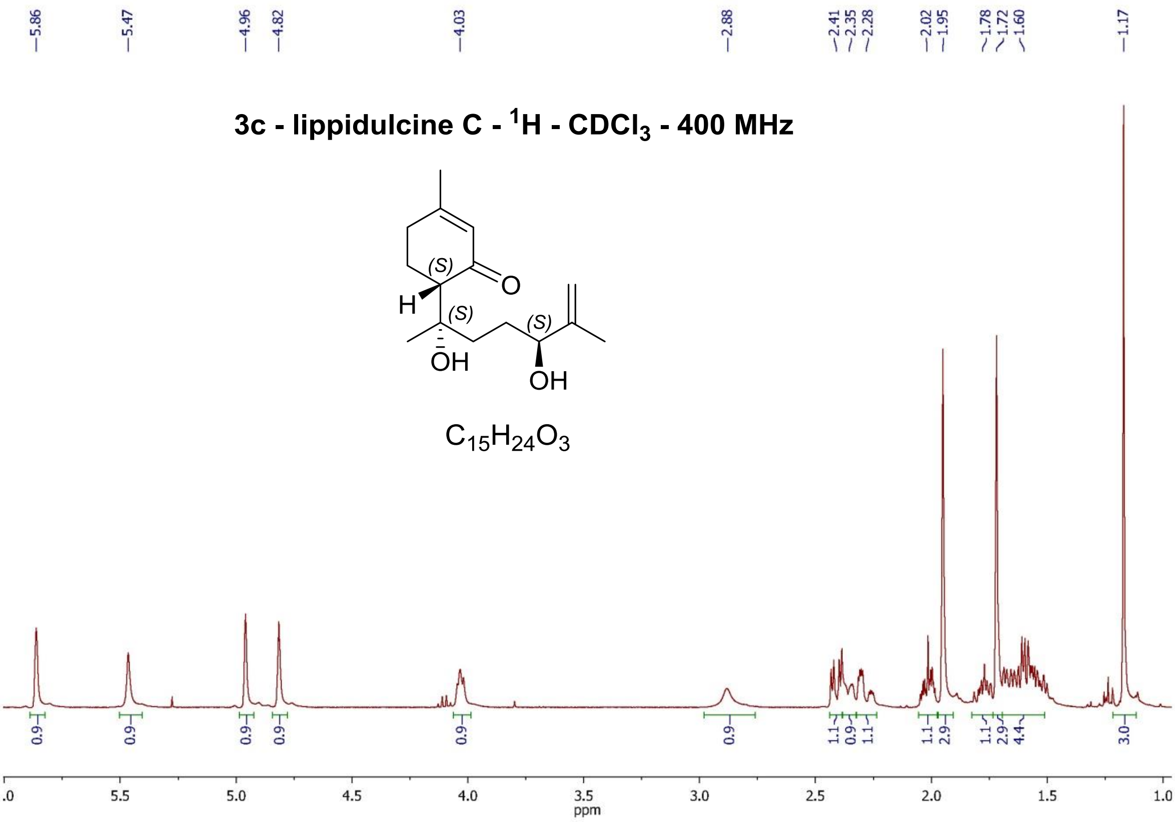
3c - lippidulcine C - ^{1}H - $CDCl_3$ - 400 MHz
(S)
(S)
(S)
H
O
OH
OH
$C_{15}H_{24}O_3$
5.86
5.47
4.96
4.82
4.03
2.88
2.41
2.35
2.28
2.02
1.95
1.78
1.72
1.60
1.17
ppm

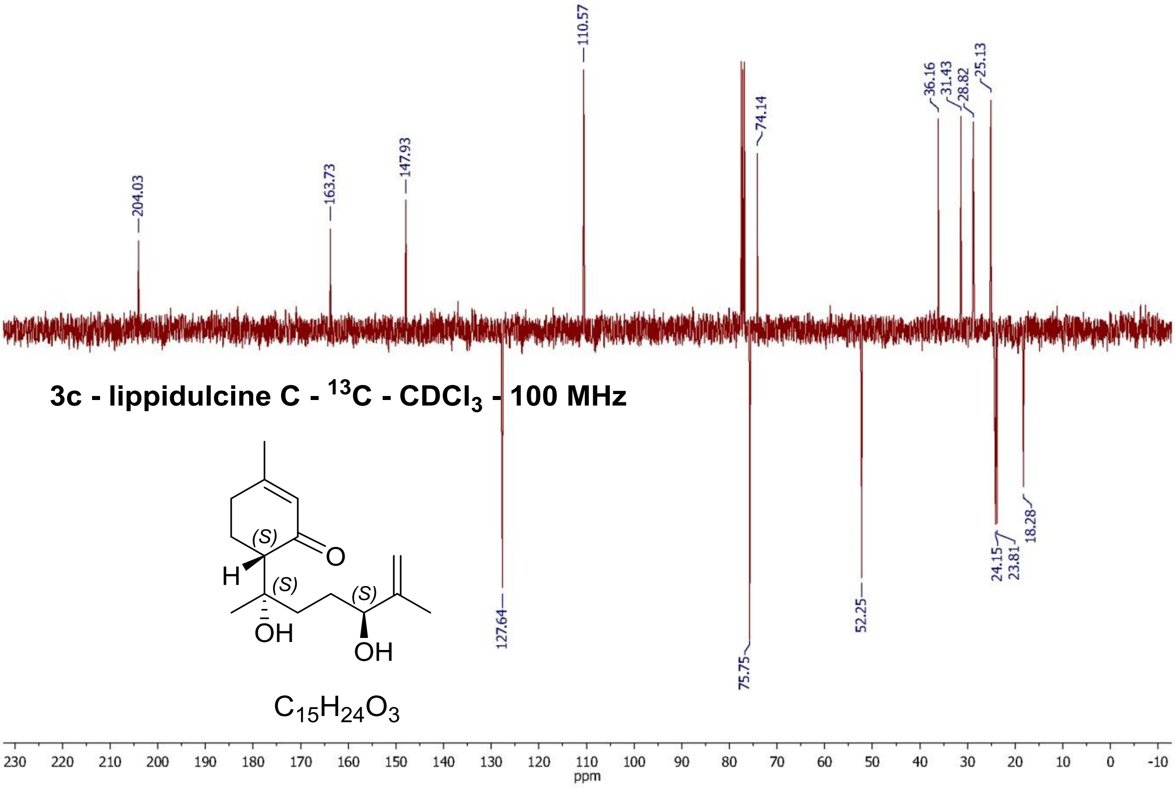
3c - lippidulcine C - ^{13}C - $CDCl_3$ - 100 MHz
(S)
(S)
(S)
H
O
OH
OH
$C_{15}H_{24}O_3$
204.03
163.73
147.93
110.57
74.14
36.16
31.43
28.82
25.13
127.64
75.75
52.25
24.15
23.81
18.28
ppm
